# Photoreceptor nanotubes mediate the *in vivo* exchange of intracellular material

**DOI:** 10.15252/embj.2020107264

**Published:** 2021-09-08

**Authors:** Arturo Ortin‐Martinez, Nicole E Yan, En Leh Samuel Tsai, Lacrimioara Comanita, Akshay Gurdita, Nobuhiko Tachibana, Zhongda C Liu, Suying Lu, Parnian Dolati, Neno T Pokrajac, Ahmed El‐Sehemy, Philip E B Nickerson, Carol Schuurmans, Rod Bremner, Valerie A Wallace

**Affiliations:** ^1^ Donald K. Johnson Eye Institute Krembil Research Institute University Health Network Toronto ON Canada; ^2^ Department of Laboratory Medicine and Pathobiology University of Toronto Toronto ON Canada; ^3^ Lunenfeld Tanenbaum Research Institute Mount Sinai Hospital Sinai Health Systems Toronto ON Canada; ^4^ Department of Biochemistry University of Toronto Toronto ON Canada; ^5^ Sunnybrook Research Institute Toronto ON Canada; ^6^ Department of Ophthalmology and Vision Sciences University of Toronto Toronto ON Canada

**Keywords:** cell therapy, material transfer, nanotubes, photoreceptors, retina, Cell Adhesion, Polarity & Cytoskeleton, Membranes & Trafficking, Neuroscience

## Abstract

Emerging evidence suggests that intracellular molecules and organelles transfer between cells during embryonic development, tissue homeostasis and disease. We and others recently showed that transplanted and host photoreceptors engage in bidirectional transfer of intracellular material in the recipient retina, a process termed material transfer (MT). We used cell transplantation, advanced tissue imaging approaches, genetic and pharmacologic interventions and primary cell culture to characterize and elucidate the mechanism of MT. We show that MT correlates with donor cell persistence and the accumulation of donor‐derived proteins, mitochondria and transcripts in acceptor cells *in vivo*. MT requires cell contact *in vitro* and is associated with the formation of stable microtubule‐containing protrusions, termed photoreceptor nanotubes (^Ph^NTs), that connect donor and host cells *in vivo* and *in vitro*. ^Ph^NTs mediate GFP transfer between connected cells *in vitro*. Furthermore, interfering with ^Ph^NT outgrowth by targeting Rho GTPase‐dependent actin remodelling inhibits MT *in vivo*. Collectively, our observations provide evidence for horizontal exchange of intracellular material via nanotube‐like connections between neurons *in vivo*.

## Graphical abstract

Bidirectional transfer of mRNAs, proteins, and mitochondria between transplanted and host retinal cells involves direct contact and formation of microtubule‐based connections between cells.

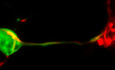

## Introduction

The mammalian retina is a central nervous system (CNS) structure that serves as a tractable system to model neural repair strategies for CNS disease (London *et al*, 2013). Like the rest of the CNS, the mammalian retina has little regenerative capacity; thus, degeneration of photoreceptors, first‐order light‐responsive neurons, results in irreversible vision impairment. Strikingly, photoreceptor transplantation into the adult retina can partially restore vision in pre‐clinical mouse models of photoreceptor dysfunction (MacLaren *et al*, 2006; Lamba *et al*, 2009; Inoue *et al*, 2010; Pearson *et al*, 2012; Santos‐Ferreira *et al*, 2015). However, we and others recently reported that donor neurons rarely integrate into the recipient retinal parenchyma and instead engage in bidirectional transfer of intracellular materials, including cytosolic GFP and Cre recombinase expressed from transgenes, as well as endogenous phototransduction proteins, which move into recipient (acceptor) photoreceptors in a process termed material transfer (MT) (Pearson *et al*, 2016; Santos‐Ferreira *et al*, 2016; Singh *et al*, 2016; Decembrini *et al*, 2017; Ortin‐Martinez *et al*, 2017). These observations suggest that cell transplantation‐mediated rescue of photoreceptor function is indirect and at least partially mediated by the transfer of intracellular material to neurons in the recipient retina. New insights into the mechanics of cytosolic MT between neurons are essential to improve our capacity to design effective cell‐based repair strategies in the CNS and to increase our foundational knowledge of inter‐neuronal communication.

The direct exchange of cytoplasmic material is rapidly emerging as an important channel for intercellular communication in the CNS, facilitating the transfer of cytosolic nucleic acids, lipids, proteins and organelles between neurons and glia (Guescini *et al*, 2012; Davis *et al*, 2014; Thomas *et al*, 2017). Cells have evolved two main mechanisms to exchange labile intracellular material: the secretion of membrane‐enclosed extracellular vesicles (EVs) (Colombo *et al*, 2014) and the formation of nanotubes, which are filopodia‐like structures that act as cytoplasmic bridges between cells to allow cytosolic exchange (Yamashita *et al*, 2018). The role of nanotubes in the exchange of organelles (Wang & Gerdes, 2015) and pathogenic proteins (Victoria & Zurzolo, 2017) is well documented *in vitro*, but evidence that these structures mediate the exchange of intracellular material between neurons *in vivo* is lacking.

Here, we used photoreceptor transplantation to investigate how MT is mediated between neurons *in vivo*. We show that donor and acceptor photoreceptors are connected by nanotube‐like protrusions, termed photoreceptor nanotubes (^Ph^NTs), that mediate the transfer of intracellular proteins and organelles. ^Ph^NTs contain microtubules, are stable after fixation, morphologically diverse and are able to mediate GFP transfer between pairs of connected photoreceptors. The outgrowth and consequently MT through ^Ph^NTs are regulated by Rho GTPase‐dependent actin remodelling. This study is the first to identify nanotube‐like structures as primary conduits for the exchange of intracellular material between photoreceptors *in vivo*.

## Results

### Efficacy of material transfer correlates with donor photoreceptor survival and is modulated by the host retina environment

**Figure 1:**
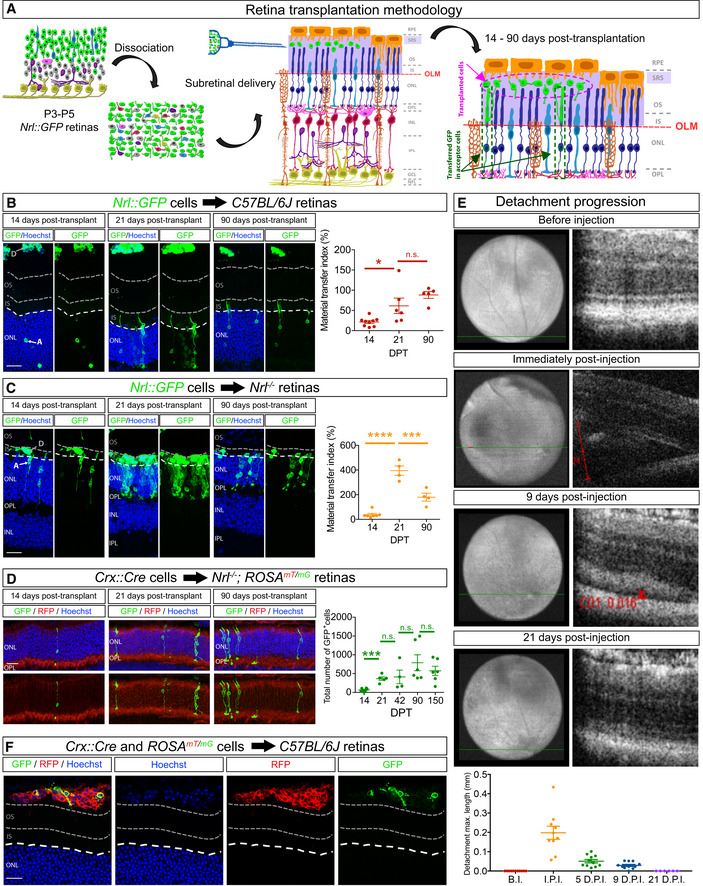
The onset of material transfer coincides with the resolution of transplant‐induced retinal detachment and can occur between donor photoreceptors located in the subretinal space APhotoreceptor transplantation schematic.B, CAnalysis of GFP transfer after transplantation of *Nrl::GFP* donor photoreceptors into wild‐type *C57BL/6J* (B) and *Nrl* ^−/−^ (C) recipient retinas after 14 (wt, *n* = 9; *Nrl* ^−/−^, *n* = 7) and 21 (wt, *n* = 6; *Nrl* ^−/−^, *n* = 4) and 90 (wt, *n* = 5; *Nrl* ^−/−^, *n* = 4) days. Tissue is stained for GFP and nuclei (Hoechst). In this and all following figures, donor cells (D) are located above, and acceptor cells with GFP (A) are located below the outer limiting membrane (white dashed line), which delimits the apical border of the outer nuclear layer (ONL). In wild‐type recipients, donor cells are typically located above the outer segment (OS) region (between the grey dashed lines). In *Nrl* ^−/−^ recipients, donor cells are located in the inner segment (IS) region. Plotted is the ratio of the numbers of GFP^+^ photoreceptors in the ONL (acceptor photoreceptors) and GFP^+^ donor photoreceptors in the subretinal space (SRS) over time.DAnalysis of Cre recombinase transfer after transplantation of *Crx::Cre* donor photoreceptors into *Nrl* ^−/−^ recipients after 14 (*n* = 6), 21 (*n* = 5), 42 (*n* = 4), 90 (*n* = 6) and 150 (*n* = 6) days. The number of acceptor photoreceptors in the recipient retina that activated the *Cre* reporter gene is quantified.EResolution of the transplant‐induced retinal detachment in *C57BL/6J* recipient retinas (*n* = 11), as measured by the maximum detachment length (mm) over time. Fundoscopy (images on the left) was used to locate the region of cell delivery for optical coherence tomgraphy (OCT) imaging (green line) shown in the images on the right. Red lines correspond to the callipers used to determine the maximum length of the detachment at each time point. Before injection (B.I.) (*n* = 11), immediately post‐injection (I.P.I.) (*n* = 10), 5 days post‐injection (5 D.P.I.) (*n* = 11), 9 days post‐injection (9 D.P.I.) (*n* = 11) and 21 days post‐injection (21 D.P.I.) (*n* = 6).FCo‐transplanted P3‐4 *Crx::Cre* and P3‐5 *ROSA^mT/mG^* donor photoreceptors into *C57BL/6J* recipient retinas show Cre reporter gene induction in donor cells located in the SRS 14 days post‐transplantation. Data information: Abbreviations: A, acceptor cells; D, donor cells; GFP, green fluorescent protein; RFP, red fluorescent protein; NFL, nerve fibre layer; GCL, ganglion cell layer; IPL, inner plexiform layer; INL, inner nuclear layer; IS, inner segments; OPL, outer plexiform layer; ONL, outer nuclear layer; OLM, outer limiting membrane; OS, outer segments; SRS, subretinal space; RPE, retinal pigmented epithelium. DPT, days post‐transplant. BI, before injection; IPI, immediately post‐injection; and DPI, days post‐injection. All data are presented as mean ± SEM; n.s. not statistically significant, ****P ≤ 0.0001, ***P < 0.001 and *P < 0.05; one‐way ANOVA with Tukey's *post hoc* multiple comparison test. Scale bars: 50 μm. Source data are available online for this figure.

**Figure EV1:**
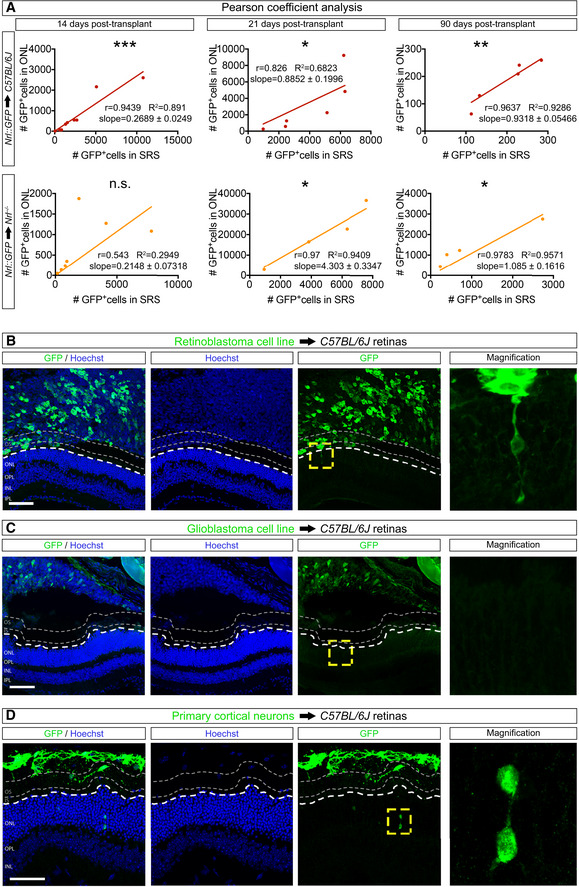
Positive linear correlation between the numbers of GFP^+^ donor and acceptor photoreceptors after transplantation. Inefficient MT in recipient photoreceptors after transplantation of cancer cell lines or primary cortical neurons ASignificant, positive linear correlation between the number of GFP^+^‐donor photoreceptors in the SRS and the number GFP^+^‐acceptor photoreceptors in the recipient retina of *C57BL/6J* (14 days (*n* = 9), 21 days (*n* = 6), 90 days (*n* = 5)) or *Nrl* ^−/−^ (14 days (*n* = 7), 21 days (*n* = 4), 90 days (*n* = 4)). r: linear correlation coefficient; R^2^: coefficient of determination; and slope: slope of the regression line.B, CTransplanted GFP^+^‐WERI‐Rb‐1 (retinoblastoma) (B) and GFP^+^‐BT088 (glioma) (C) cell lines result in inefficient MT.DMT between transplanted primary GFP^+^‐cortical neurons and the recipient retina is also inefficient. Data information: n.s. not statistically significant, ***P < 0.001, **P < 0.01 and *P < 0.05; Pearson correlation coefficient and linear regression analysis. White dashed lines delimit the apical side of the ONL of the recipient retina; grey dashed lines delimit the apical border of the inner segments in the recipient retina. Scale bars: 50 μm. Source data are available online for this figure.

Retinal photoreceptor transplantation involves transplanting GFP^+^‐rod photoreceptor precursor cells from *Nrl::GFP* mice (Akimoto *et al*, 2006), hereafter referred to as *donor photoreceptors*, into the subretinal space (SRS) of the adult mouse retina (Pearson *et al*, 2017, 2016; Santos‐Ferreira *et al*, 2017, 2016; Singh *et al*, 2017, 2016; Decembrini *et al*, 2017; Ortin‐Martinez *et al*, 2017; Fig 1A). Grafted GFP^+^‐rod photoreceptors do not migrate into the recipient retina, and instead, their cell bodies remain sequestered in the SRS. Thus, the accumulation of fluorescent reporter proteins in adjacent photoreceptors in the recipients, hereafter referred to as *acceptor photoreceptors*, occurs through MT. To investigate the kinetics of MT and the relationship between donor and acceptor photoreceptors, we monitored the numbers of GFP^+^‐donor and GFP^+^‐acceptor photoreceptors over time in wild‐type and *Nrl* ^−/−^ recipients, the latter being a mouse strain with enhanced levels of GFP transfer after transplantation (Santos‐Ferreira *et al*, 2015; Decembrini *et al*, 2017; Ortin‐Martinez *et al*, 2017). We found that there was a positive linear correlation between the numbers of GFP^+^‐donor and GFP^+^‐acceptor photoreceptors over time in both types of transplant recipients (Fig  EV1A), indicating that there is a tight correlation between accumulation of GFP in acceptor photoreceptors and donor photoreceptor survival. We normalized the data as the ratio of GFP^+^‐acceptor/GFP^+^‐donor cells, hereafter referred to as MT index, which provided a rigorous metric to evaluate the phenomenon of MT and mitigated inter‐recipient transplant variability. Notably, the MT index in wild‐type recipients increased from 14 days to reach a maximum by 21 days post‐transplant (Fig 1B and C), coinciding with the time when the retinal detachment injury induced by transplant surgery was resolved (Fig 1E). As expected, the MT index was ∼5‐fold greater in *Nrl* ^−/−^ compared with wild‐type recipients (Fig 1C). Notably, because these data are normalized to donor cell number, the increase in MT *Nrl* ^−/−^ recipients is independent of donor photoreceptor survival. Taken together, our results show that MT is, in part, a function of the number of surviving donor photoreceptors, but also that recipient photoreceptors differ in their capacity to support MT from an identical pool of transplanted photoreceptors.

We noted a decline in the absolute numbers of GFP^+^‐acceptor photoreceptors in both types of recipient mice by 90 days post‐transplantation, even though the numbers of GFP^+^‐acceptor and GFP^+^‐donor photoreceptors were still tightly correlated (Fig EV1A). One explanation for this parallel decline in both populations was that acceptor photoreceptors required constant replenishment of material from donor photoreceptors to maintain detectable levels of GFP. If this model were true, then the number of labelled acceptor photoreceptors should not decline in a situation where MT induces constitutive expression of a fluorescent reporter. To test this idea, we transplanted retinal cells from *Crx::Cre* mice (Prasov & Glaser, 2012) in which all photoreceptors express Cre recombinase, into adult *Nrl* ^−/−^ mice carrying a *ROSA^mT/mG^* Cre reporter, and monitored the Cre‐dependent induction of membrane GFP (mG) expression in acceptor photoreceptors (Fig 1D). The number of mG^+^‐acceptor photoreceptors reached a plateau by 21 days and did not decline even after 3 and 5 months post‐transplant (Fig 1D). From these data, we conclude that acceptor photoreceptors that receive transferred material are not selectively less viable and that MT requires constant replenishment of labile material from donor photoreceptors.

### Material transfer is specific to photoreceptors and their precursors

**Figure EV2:**
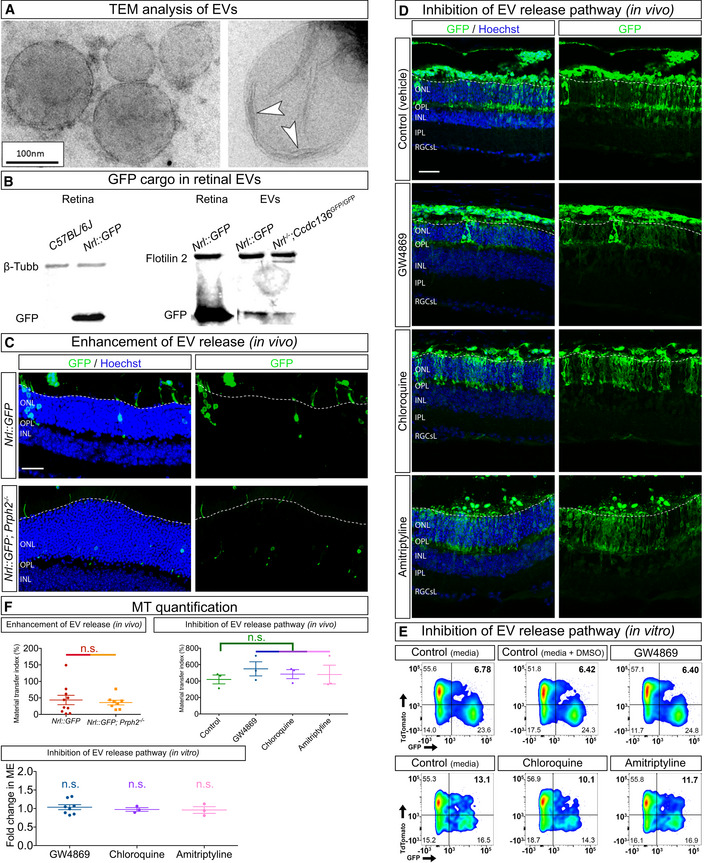
Photoreceptor‐derived extracellular vesicles do not mediate GFP transfer AElectron microscopy images of EVs harvested from photoreceptor cultures. Arrowheads point to the bilipid membrane.BWestern blot analysis of EVs from *Nrl::GFP* and *Nrl* ^−/−^ *; Ccdc136^GFP/GFP^* photoreceptors confirm the presence of GFP in vesicles.C
*Nrl::GFP; Prphr2* ^−/−^ transplanted photoreceptors (*n* = 8) show no differences in MT index compared to *Nrl::GFP* transplanted photoreceptors (*n* = 10).DPharmacological inhibition of different vesicle‐release pathways: GW4869 (exosome secretion blocker); chloroquine (autophagy induction blocker); and amitriptyline (ectosome secretion blocker) show no effect on MT *in vivo* (*n* = 3 per group).EPharmacological inhibition of different vesicle‐release pathways: GW4869 (exosome secretion blocker); chloroquine (autophagy induction blocker); and amitriptyline (ectosome secretion blocker) show no effect on MT *in vitro* (*n* = 8, *n* = 3 and *n* = 3, respectively).FQuantification for C–E. Data information: All data are presented as mean ± SEM; n.s. not statistically significant, one‐way ANOVA with Tukey's *post hoc* multiple comparison test. White dashed lines delimit the apical side of the ONL of the recipient retina. Scale bars: A: 100 nm. C, D: 50 μm. Source data are available online for this figure.

**Figure EV3:**
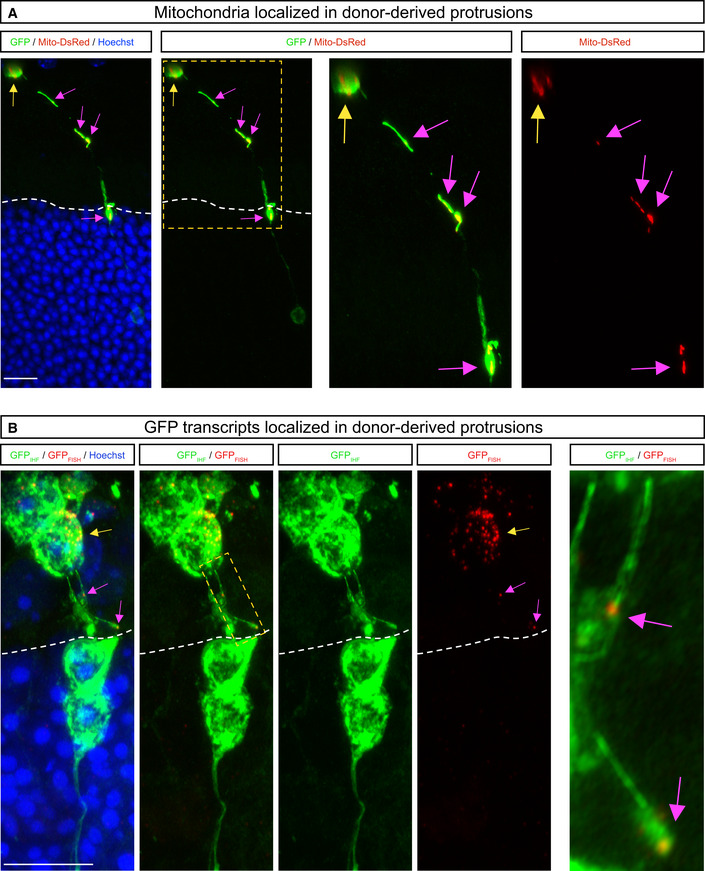
Mitochondria and GFP transcripts are found in cell protrusions A, BHigh magnification confocal image shows Mito‐DsRed^+^ mitochondria (A) and GFP mRNA (B) in a protrusion connected to an acceptor photoreceptor in wild‐type retinas transplanted with *Nrl::GFP* donor photoreceptors. White dashed lines delimit the apical side of the ONL of the recipient retina. Yellow arrows point to Mito‐DsRed^+^ puncta (A) or transcripts (B) in the donor cells. Pink arrows point to Mito‐DsRed^+^ puncta (A) or transcripts (B) in the acceptor cells. Scale bars: 10 μm.

**Figure EV4:**
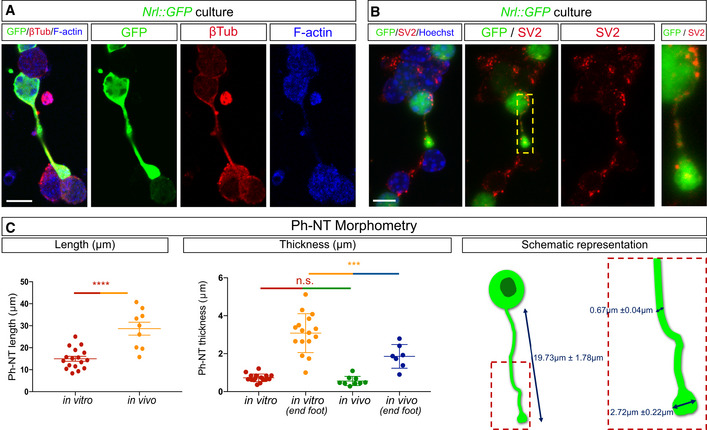
Further characterization of cell protrusions A, Bβ‐tubulin, F‐actin (A), SV2 (B) and GFP staining in photoreceptor co‐cultures of wild‐type and *Nrl::GFP* retinal cells shows *Nrl::GFP* donor photoreceptors connected with acceptor photoreceptors via protrusions that co‐localize with β‐tubulin and F‐actin and SV2.C
*In vitro* (protrusion length (*n* = 17), protrusion end‐foot (*n* = 16) from a total sample (*n* = 9) (biological replicate (*n* = 3) technical replicates (*n* = 3) per each biological replicate) and *in vivo* (protrusion length *n* = 9) protrusion end‐foot (*n* = 7) from wt cells transplanted in wt animals (*n* = 7)) measurements of length and thickness of protrusions. Data information: All data are presented as mean ± SEM; n.s. not statistically significant, ****P ≤ 0.0001 and ***P < 0.001. *t*‐Test or one‐way ANOVA with Tukey's *post hoc* multiple comparison test. Scale bars: D: 50 μm. A, B: 5 μm. Source data are available online for this figure.

**Figure EV5:**
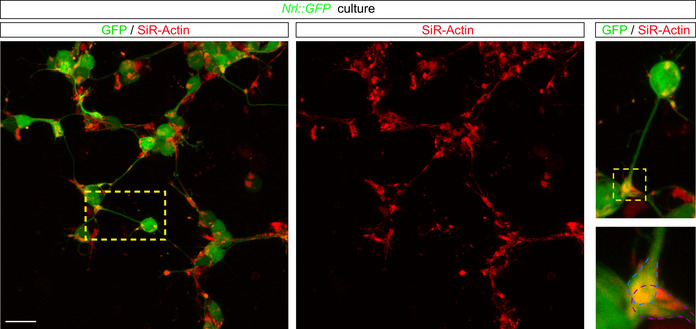
*In vitro* characterization of protrusions Confocal image of live *Nrl::GFP* cultures detailing (right panel) that the terminus of a protrusion at the contact point between two photoreceptors contains actin (SiR‐Actin^+^). Blue and purple dashed lines delimit the end‐foot of two different protrusions contacting the soma of another cell. Scale bar: 10 μm.

While our findings show that accumulation of transferred material in acceptor photoreceptors depends on the presence of donor photoreceptors, it is not clear how selective this process is for photoreceptor donors, as it could simply be that acceptor photoreceptors have a high propensity to accumulate material from any type of cell. To address this issue, we compared the MT index after transplanting GFP^+^‐human tumour cells (Fig EV1, EV2, EV3, EV4, EV5). While engrafted cells were readily detected in the SRS, the incidence of MT was markedly low (Fig EV1B and C). We did similar experiments with embryonic cortical cells and observed only rare examples of GFP accumulation in acceptor photoreceptors (Fig EV1D). Thus, efficient MT appears to be neuron lineage specific, as it is not recapitulated by tumour cells or cortical neurons.

Finally, to investigate how photoreceptor maturation affects MT we also asked whether MT can occur between immature photoreceptors. We transplanted mixed retinal dissociates from P3–5 *Crx::Cre* and *ROSA^mT/mG^* mice to the SRS of wild‐type adult mice and tracked Cre transfer, marked by the induction of mG expression, within the bolus. We detected “recombined” mG^+^ cells in the subretinal cell deposit, indicating that Cre is transferred between transplanted cells (Fig 1F). Since photoreceptor precursors do not fully mature after transplantation (Eberle *et al*, 2012), this result indicates that acceptor photoreceptors do not need to be mature for MT to occur and that this mode of cell–cell communication can occur outside of the neuroepithelial organization of the retina.

### Material transfer is associated with the accumulation of donor‐derived proteins and transcripts in acceptor cells

**Figure 2:**
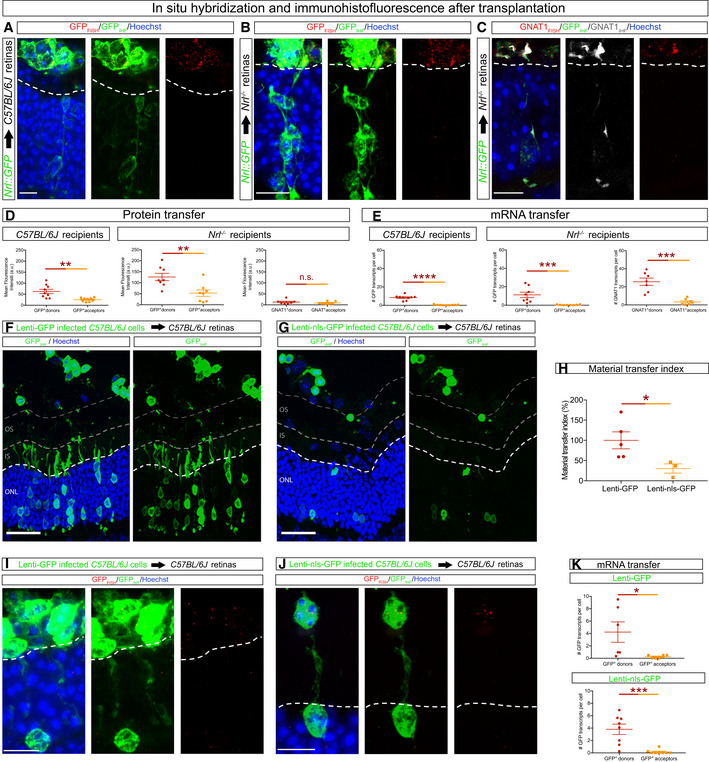
Transfer of donor‐derived proteins and transcripts to host photoreceptors A–CCombined FISH and IHF to detect GFP (A, B) and GNAT1 (C) 21 days post‐transplantation of *Nrl::GFP* photoreceptors into *C57BL/6J* (A) and *Nrl* ^−/−^ (B, C) recipients.D, EDifferences in mean fluorescence intensity of GFP and GNAT1 protein staining (D) and transcripts (E) between donor and acceptor photoreceptors (GFP: wt recipients, *n* = 9 images from three transplanted animals; *Nrl* ^−/−^ recipients *n* = 8 images from four transplanted animals. GNAT1: *Nrl* ^−/−^ recipients, *n* = 7 images from three transplanted animals).F–KFISH and IHF for GFP and GFP transcripts in *C57BL/6J* recipients transplanted with lentiviral‐infected donor cells expressing cytoplasmic GFP (Lenti ‐GFP) (F, I) and nuclear localized GFP (Lenti‐nls‐GFP) (G, J). H, MT index for cytosolic (Lenti‐GFP, *n* = 5) and nuclear localized (Lenti‐nls‐GFP, *n* = 3). K, Quantification of transcripts in donor and acceptor photoreceptors (Lenti‐GFP, *n* = 6 images from three transplanted animals; Lenti‐nls‐GFP, *n* = 8 images from four transplanted animals). Data information: All data are presented as mean ± SEM; n.s. not statistically significant, ****P ≤ 0.0001, ***P < 0.001, **P < 0.01 and *P < 0.05; one‐way ANOVA with Tukey's *post hoc* multiple comparison test. White dashed line delimits the apical border of the outer nuclear layer; grey dashed lines indicate the outer segment (OS) region. IS, inner segments. Scale bars: A–C, I, J: 10 μm. F, G: 50 μm. Source data are available online for this figure.

The accumulation of fluorescent reporter proteins in acceptor photoreceptors could be mediated by protein and/or transcript transfer. We used immunostaining and RNAscope to compare the levels of GFP protein and transcript, respectively, in donor and acceptor cells in transplanted wild‐type and *Nrl* ^−/−^ recipients (Fig 2A and B). GFP fluorescence intensity and transcript levels were approximately 2‐fold and 20‐fold lower, respectively, in acceptor cells relative to donor cells in both types of recipients (Fig 2A–E). To investigate whether there was a similar relationship for the transfer of endogenously expressed proteins, we examined transfer of GNAT1, a rod photoreceptor specific gene, in *Nrl* ^−/−^ recipient photoreceptors, which do not express GNAT1. We found higher levels of GNAT1 protein compared with transcripts in *Nrl* ^−/−^ acceptor photoreceptors (Fig 2C–E). These findings indicate that mRNA can transfer between cells, but they do not distinguish whether the accumulation of donor‐derived proteins in acceptor cells is due to direct protein transfer or translation from transferred mRNA. We reasoned that if transcripts were the primary cargo for transfer, then the accumulation of donor cell‐derived nuclear and cytosolic proteins in acceptor cells should be similar. We used lentiviruses (Appendix Fig S1A) to express similar levels of cytosolic and nuclear localized GFP (nls‐GFP) in transplanted donor cells and found that cytosolic GFP transfer was significantly increased relative to nls‐GFP transfer (Fig 2F–K). Moreover, this difference in the transfer of cytosolic versus nuclear proteins was not associated with significant differences in the transcript levels in acceptor cells, despite similar levels of these transcripts in donor cells (Fig 2H and K). Taken together, these data support a model in which the accumulation of donor‐derived material can be mediated by mRNA as well as direct protein transfer.

### Mitochondria transfer in the context of photoreceptor transplantation

**Figure 3:**
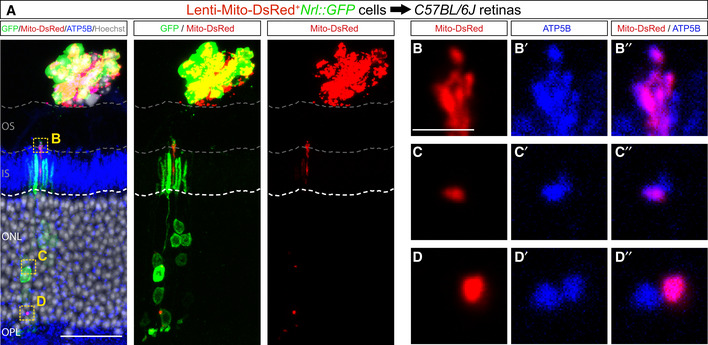
Mitochondria transfer to photoreceptors *in vivo* ADetection of GFP, Mito‐DsRed, mitochondria marker (ATP5B, blue) and nuclei (Hoechst, white) in wild‐type *C57BL/*6J recipient retinas 21 days after transplantation with Mito‐DsRed‐expressing *Nrl::GFP* donor photoreceptors.B–D’’Higher magnification of regions in A (indicated by yellow dashed boxes) showing co‐localization of ATP5B and Mito‐DsRed‐labelled mitochondria in the outer segments (B), soma (C) and the axon (D) of acceptor photoreceptors. Data information: White dashed line delimits the apical border of the outer nuclear layer; grey dashed lines delimit the inner segments and/or outer segments of recipient photoreceptors. Scale bar: A: 50 μm. B–D’’: 5 μm.

To ask whether MT extends to organelles, we investigated mitochondria transfer *in vivo*. We transplanted GFP^+^‐donor photoreceptors from *Nrl::GFP* mice that were infected with lentiviruses to express Mito‐DsRed, a mitochondrial targeting sequence fused to DsRed. At 21 days post‐transplantation into adult retinas, we observed DsRed^+^‐puncta in acceptor photoreceptors (Fig 3A). While we cannot exclude the possibility that this result is a consequence of transcript and/or Mito‐tagged protein transfer, we suggest that it reflects the transfer of donor mitochondria to acceptor cells for two reasons. First, all Mito‐DsRed+ puncta in acceptor cells co‐localized with a mitochondria marker, ATP5B (Fig 3B–D’’). Second, the scenario where the mito‐tagged protein is imported into acceptor cell mitochondria after transfer is unlikely as it would require engagement of the protein unfolding machinery (Pfanner & Truscott, 2002). Thus, taken together, the presence of Mito‐DsRed+ mitochondria in acceptor cells is consistent with the possibility that large cytoplasmic components, such as mitochondria, are also substrates for MT.

### Photoreceptor material transfer is bidirectional and requires cell contact

**Figure 4:**
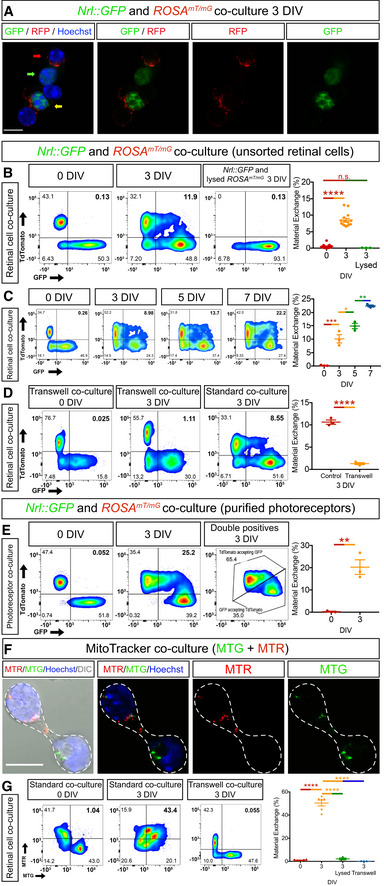
Characterization of material transfer in primary photoreceptor cell culture AImmunofluorescence of *Nrl::GFP* and *ROSA^mT/mG^* retina co‐cultures after 3 days *in vitro* (DIV) shows cells expressing cytoplasmic GFP only (green arrow), membrane RFP only (red arrow) or both (yellow arrow). Nuclei are stained with Hoechst (blue).B–EFlow cytometric analysis and quantification of cytoplasmic and membrane protein exchange in co‐cultures. Material transfer is quantified as the percentage of GFP^+^mT^+^ CD73^+^‐photoreceptors over the total population of fluorescent GFP^+^ and mT^+^ ‐CD73^+^‐photoreceptors in the cultures (0 DIV, *n* = 17; 3 DIV, *n* = 15). *Nrl::GFP* photoreceptors do not accumulate mT after co‐culture with lysed *ROSA^mT/mG^* cells (3 DIV, *n* = 3; 3 DIV lysed, *n* = 3) (B). Material transfer increases over time in co‐cultures (*n* = 3 per time point) (C), does not occur when *Nrl::GFP* and *ROSA^mT/mG^* photoreceptors are separated by a transwell insert (control, *n* = 3; transwell, *n* = 3) (D) and also occurs in cultures of purified photoreceptors (*n* = 3) (E).F, G
*C57BL/6J* cells with mitochondria labelled with MitoTracker‐Green (MTG) or MitoTracker‐Red (MTR) exchange mitochondria after 3 DIV (0 DIV, *n* = 6; 3 DIV, *n* = 6). White dashed line delimitates both cell somas and the protrusion connecting them (F). No exchange is observed when the two photoreceptor populations are separated by a transwell (*n* = 3), nor in co‐cultures of live MTG‐labelled and lysed MTR‐labelled cells (*n* = 3) (G). Data information: Abbreviations: DIV, days *in vitro*; GFP, green fluorescent protein; RFP, red fluorescent protein; and DIC, differential interference contrast microscopy. All data presented as mean ± SEM; n.s. not statistically significant, ****P ≤ 0.0001, ***P < 0.001, **P < 0.01 and *P < 0.05; one‐way ANOVA with Tukey's *post hoc* multiple comparison test and unpaired *t*‐test. Scale bars: 10 μm. Source data are available online for this figure.

To further characterize MT, we established an *in vitro* model where we tracked fluorescent protein exchange in co‐cultures of GFP^+^ (from *Nrl::GFP* mice) and membrane tethered tdTomato^+^ (mT^+^) (from *ROSA^mT/mG^* mice) photoreceptors (Appendix Fig S2A). After 3 days *in vitro*, we detected GFP^+^mT^+^ cells in these co‐cultures (Fig 4A), indicating that transfer of fluorescent proteins did occur *in vitro*. We then used flow cytometry to quantify the frequency of GFP^+^mT^+^ photoreceptors under various conditions (Fig 4B–E and Appendix Fig S2B). We found that MT *in vitro* is cumulative (Fig 4C) and requires live cells, as it was not detected when viable cells were co‐cultured with fluorescent debris from dead cells (Fig 4B, Appendix Fig S2D). Furthermore, MT did not require the presence of other neuronal cell types in the culture, as it occurred in co‐cultures of purified photoreceptors (Fig 4E). We also asked whether mitochondria could be exchanged by co‐culturing retinal cell dissociates labelled with red and green MitoTracker dyes. Using imaging and flow cytometry, we found that cells exchanged MitoTracker‐labelled puncta and that this exchange required live‐labelled cells (Fig 4F and G, Appendix Fig S2C). Based on these data, we conclude that cytosolic and membrane‐associated proteins, as well as mitochondria, can transfer between photoreceptor precursor cells *in vitro*.

Extracellular vesicles can transfer proteins, nucleic acids and organelles between cells (Zappulli *et al*, 2016) and GFP‐labelled donor photoreceptors from *Nrl::GFP* retinal dissociates secreted bilipid‐encased EVs that contained GFP protein (Fig EV2A and B). However, several lines of evidence indicate that EV exchange is not the primary mechanism of MT of proteins and mitochondria. We did not detect MT when photoreceptors expressing fluorescent proteins or labelled with MitoTracker dyes were separated by a transwell, indicating that MT *in vitro* required cell contact (Fig 4G). EVs could still contribute to contact‐dependent MT, particularly if they traffic cargo along protrusions, as has been shown in other contexts (Heusermann *et al*, 2016). However, pharmacologic inhibition of EV secretion had no effect on MT of fluorescent proteins *in vitro* and *in vivo* (Fig EV2, EV3, EV4, EV5). Finally, MT was not increased following transplantation of GFP‐labelled donor photoreceptors isolated from *Nrl::GFP; Prphr2* ^−/−^ mice, which have genetically enhanced EV secretion (Salinas *et al*, 2017) (Fig EV2C). While these observations do not preclude a role for EVs in GFP transfer, they do suggest that uptake of randomly secreted EVs is not the primary mechanism of MT *in vivo* and *in vitro*.

### Donor and acceptor photoreceptors engaging in material transfer are physically connected by cellular protrusions

**Figure 5:**
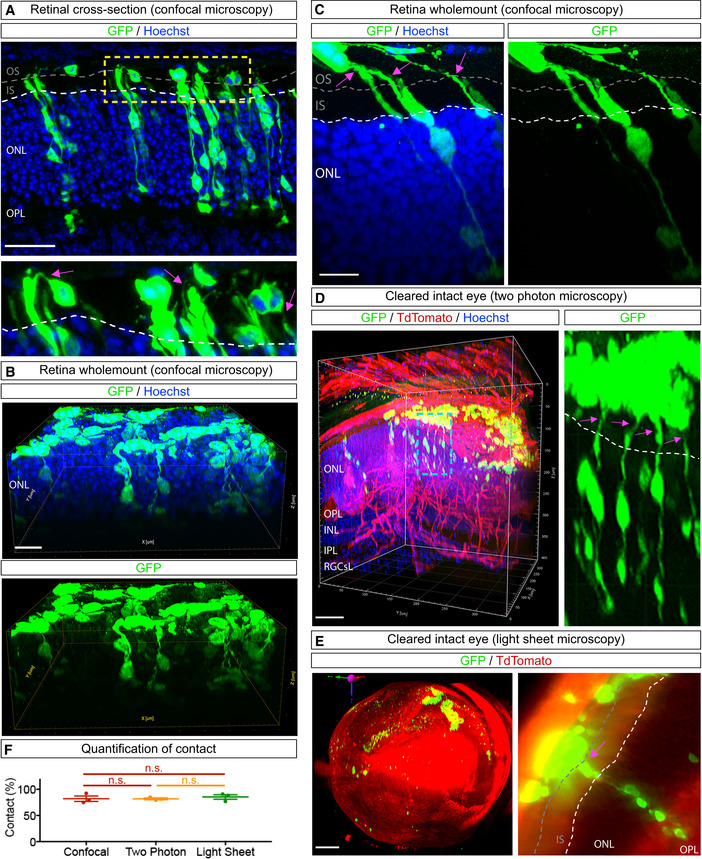
GFP‐labelled acceptor photoreceptors are connected to transplanted donor photoreceptors via cell protrusions AMaximum intensity projection of a confocal micrograph of a cryostat section from a *Nrl* ^−/−^ recipient retina 21 days after transplantation with *Nrl::GFP* donor photoreceptors, showing protrusions (pink arrows) connecting donor and acceptor photoreceptors.B, C3D reconstruction of confocal microscopy images of whole‐mounted retinas from *Nrl* ^−/−^ (B) and *C57BL6/J* (C) recipients 21 days after transplantation with *Nrl::GFP* donor photoreceptors (top layer) shows that donor and GFP^+^ acceptor photoreceptors appear to be attached via protrusions. Higher magnification 3D reconstruction (C) showing protrusions (pink arrows) and GFP labelling in acceptor cells.D, E
*Left:* 3D reconstruction of two‐photon (D) and light sheet (E) microscopy images of intact cleared *ROSA^mT/mG^* eye showing that GFP^+^‐donor and GFP^+^‐acceptor photoreceptors are in the same area. D, *Right:* A high magnification image showing GFP^+^‐donor and GFP^+^‐acceptor photoreceptors connected by protrusions (pink arrows). E, *Right*: In detail, donor and acceptor photoreceptors also appear connected through cell protrusions (pink arrow).FPercentage of GFP^+^‐acceptor photoreceptors connected via a donor cell protrusion across different imaging modalities (*n* = 3 animals per imaging technique). Data information: Abbreviations: RGCsL, retina ganglion cells layer. All data presented as mean ± SEM; n.s. not statistically significant, one‐way ANOVA with Tukey's *post hoc* multiple comparison test. White dashed lines delimit the apical side of the ONL in the recipient retina; grey dashed lines delimit the apical border of recipient photoreceptor inner segments. Scale bars: A, B, D: 50 μm. C: 20 μm. E: 1000 μm. Source data are available online for this figure.

We next examined transplanted retinas for evidence of physical contacts between donor and acceptor photoreceptors. Consistent with previous observations (Ortin‐Martinez *et al*, 2017), we observed thin protrusions that appeared to connect GFP^+^ donor and GFP^+^ acceptor photoreceptors in transplanted retinas (Fig 5A). However, these intercellular contacts were observed infrequently, possibly because the protrusions and cell bodies are in different tissue sections. Therefore, we examined the donor–acceptor photoreceptor interface in retinal whole mounts, an alternative approach that does not involve tissue sectioning. In adult retinas at 21 days post‐transplantation of *Nrl::GFP* photoreceptors brightly labelled donor photoreceptors were retained on the apical surface of retina whole mounts. Donor cells were distinguished from underlying GFP^+^‐acceptor photoreceptors by their more intense GFP labelling and by the location of their soma apical to the outer limiting membrane (OLM) of the recipient photoreceptor layer (Fig 5B, Movie EV1). Higher magnification imaging revealed that GFP^+^‐donor photoreceptors extended protrusions that terminated at the OLM or that contacted GFP^+^‐acceptor photoreceptors (Fig 5C, Movie EV2). These observations suggested that the OLM could be a barrier to the establishment of contact between donor and acceptor photoreceptors, and that not all donor neuron protrusion contacts result in GFP transfer (Fig 5C, Movie EV2).

Using a tissue clearing protocol for whole intact eyes (Gurdita *et al*, 2021), we next imaged single donor photoreceptors in the SRS by two‐photon and light sheet fluorescence microscopy (Fig 5D and E, Movies EV3 and EV4). Consistent with our observations in whole mounts, we observed protrusions connecting GFP^+^‐donor and GFP^+^‐acceptor photoreceptors in intact eyes (Fig 5D and E, Movies EV3 and EV4). Quantification across the three imaging modalities showed that in the majority of cases (∼80%), the apical regions of GFP^+^‐acceptor photoreceptors were connected to donor photoreceptors via a protrusion (Fig 5F), revealing a previously unappreciated aspect of the donor–acceptor neuron interface. GFP^+^‐donor cell protrusions also contained transferable cargo, such as mitochondria and GFP mRNA (Fig EV3). We therefore propose that these protrusions connecting donor and acceptor photoreceptors are relevant to MT, either serving as conduits for MT or serving a permissive adhesive function that allows for MT to occur over a short range.

### Donor photoreceptor protrusions resemble immature neurites and can mediate GFP exchange *in vitro*

**Figure 6:**
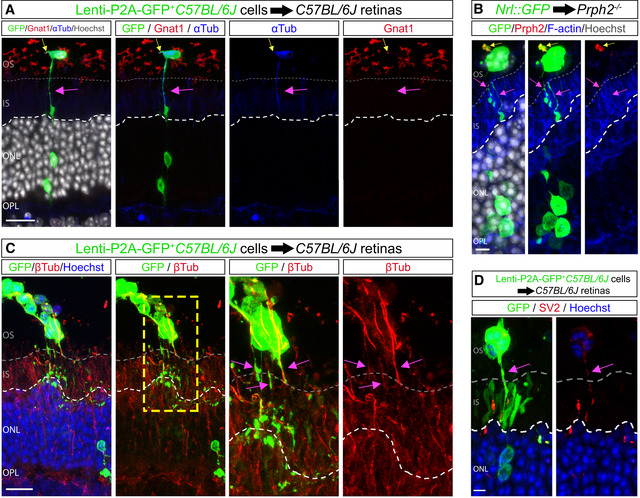
Characterization of protrusions that connect donor and acceptor photoreceptors in transplanted retinas AConfocal image shows a Lenti‐GFP‐infected donor photoreceptor (yellow arrow) connected to an acceptor photoreceptor. The donor protrusion (pink arrow) co‐localizes with α‐tubulin but not the outer segment marker GNAT1.B
*Nrl::GFP* donor photoreceptors transplanted in a *Prphr2* ^−/−^ recipient retina exhibit polarized Prphr2 expression (yellow arrow) on the apical side of the donor photoreceptor. Basal protrusions (pink arrows) indicate donor protrusions that contact the host retina.C, DExpression of β‐tubulin (C), SV2 (D) and GFP in the protrusions connecting Lenti‐GFP‐infected *C57BL/6J* donor photoreceptors with acceptor photoreceptors. Pink arrows point to the protrusions connecting donor and acceptor cells. Data information: White dashed lines delimit the apical side of the ONL of the recipient retina; grey dashed lines delimit the apical border of the inner segments in the recipient retina. Scale bars: A, C: 50 μm. B, D: 10 μm.

Transplanted photoreceptors have been reported to extend protrusions (Warre‐Cornish *et al*, 2014), and these could be related to the apical or basal processes of mature rod photoreceptors, outer segments (OS) and axons, respectively (Sarin *et al*, 2018). While donor cells exhibited polarized staining with OS markers GNAT1 and Peripherin2 (Fig 6A and B), this was frequently oriented towards the retinal pigmented epithelium and not enriched in the protrusions connecting donor and acceptor cells, indicating that they are distinct from OS. Instead, these protrusions were similar to neurites, based on the presence of microtubules (Fig 6A and C), tortuosity and spherule‐like terminals at their distal tips (Fig 6B). However, these protrusions appeared to be distinct from mature photoreceptor axons, as presynaptic markers were not enriched at their termini, rather these were scattered along the length of donor cell protrusions (Fig 6D).

**Figure 7:**
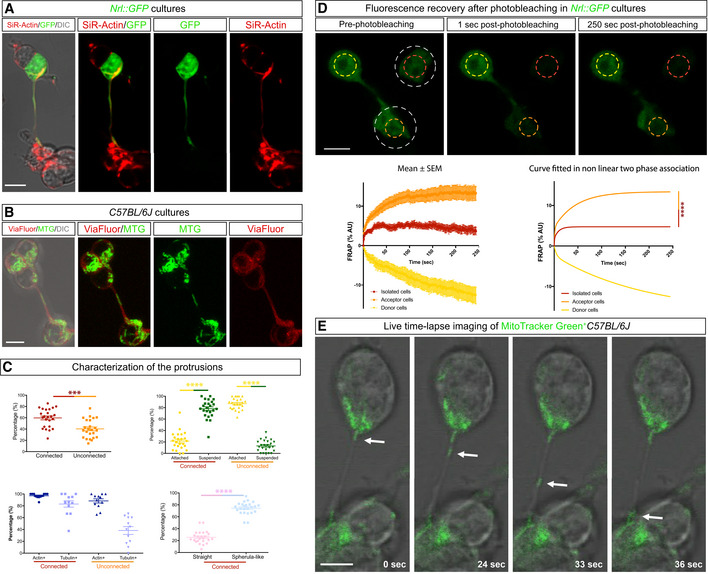
Analysis of photoreceptor protrusions and GFP transfer *in vitro* A, BExamples of live *Nrl::GFP* and *C57BL/6J* retinal cultures showing actin^+^ (SirActin) and GFP^+^ (A) and tubulin^+^ (ViaFluor) and MitoTracker Green^+^ (MTG) protrusions (B) connecting photoreceptors. DIC, differential interference contrast microscopy.CQuantification of photoreceptor protrusions in *Nrl::GFP* retinal cultures (culture wells, *n* = 8, images, *n* = 24, protrusions, *n* = 868). Data are presented as mean ± SEM.DImages from FRAP experiments carried out on protrusion connected and isolated *Nrl::GFP* cultures pre‐ and post‐photobleaching. Area of photobleaching (white dashed circles). Analysed regions (yellow, orange and red dashed circles). Photoreceptor FRAP results shown from multiple experiments (isolated cells, *n* = 14; connected to recipient, *n* = 21; connected donor, *n* = 21). All data presented as percentage, mean ± SEM and fitted to the curve in a non‐linear regression with two‐phase association.ETime lapse imaging showing movement of MitoTracker Green^+^ puncta in photoreceptors connected by a protrusion. White arrows point to MTG^+^ puncta inside of the protrusion during the time lapse imaging. Data information: n.s. not statistically significant, ****P ≤ 0.0001 and ***P < 0.001; unpaired *t*‐test and extra‐sum‐of‐squares *F*‐test (Y0 constrained to 0.0). Scale bars: 5 μm. Source data are available online for this figure.

To investigate the capacity for these protrusions to mediate MT *in vitro*, we first confirmed that they formed *ex vivo*. In fixed (Fig EV4A and B) and live (Fig 7A and B) cultures, we detected microtubule^+^‐protrusions that connected to photoreceptors and contained presynaptic markers (Fig EV4B) and transferrable cargo, including mitochondria and GFP (Fig 7A and B, amd EV4A), along their length. These protrusions were similar in size and morphology to those we identified *in vivo* (Fig EV4C). To further characterize the types of protrusions that form *in vitro*, we analysed live *Nrl::GFP* cultures that were stained with vital actin and tubulin dyes. Protrusions could be grouped into two main categories: those that connected cells, which comprised the majority (∼60%), and those that were unconnected (∼40%). While nearly all protrusions stained with vital actin, the microtubule content was higher in connected (83%) compared with unconnected (38%) protrusions (Figs 7C and EV5). Analyses of fixed and live cultures revealed that the majority of the connected protrusions were suspended above the dish (Fig 7C, Movie EV5) and terminated with a distal swelling or spherule‐like morphology (Figs 7C and EV5). In contrast, the majority of unconnected protrusions were attached to the substrate and terminated with a classical growth cone morphology (Movie EV6). Finally, live imaging analysis revealed that MitoTracker^+^ puncta moved along connected protrusions (Fig 7B and E, and Movie EV7). To investigate whether connected protrusions could transfer material, we analysed the fluorescence recovery after photobleaching (FRAP) of *Nrl::GFP* cultures. Isolated photoreceptors (*n* = 14) recovered a small fraction of the original GFP fluorescence (4%) over the entire imaging interval of 250 s (Fig 7D), possibly due to redistribution of the remaining GFP in the cell. In contrast, photoreceptors that were connected to an adjacent cell via a protrusion (*n* = 21) recovered ∼15% of their fluorescence over the entire imaging interval (Fig 7D). The recovery of GFP in the bleached cell was more rapid than would be expected for *de novo* protein synthesis (Kourtis & Tavernarakis, 2009) and was associated with a significant and reciprocal reduction in GFP intensity in the unbleached connected cell of the pair (*n* = 22) (Fig 7D). Taken together, cultured and transplanted photoreceptors form similar protrusions and *in vitro* analysis reveals that these connected protrusions are capable of trafficking mitochondria and transferring GFP between cells.

Based on these results, we suggest that donor photoreceptors extend microtubule^+^‐protrusions to form stable connections with acceptor photoreceptors *in vivo*. Furthermore, we provide evidence that these protrusions do not resemble outer segments nor mature axons of photoreceptors. Rather, because they are suspended above the substratum of the dish and mediate the transfer of intracellular material between connected photoreceptors, we name them photoreceptor nanotubes (^Ph^NT).

### Rho GTPase actin remodelling regulates ^Ph^NT extension and material transfer

**Figure 8:**
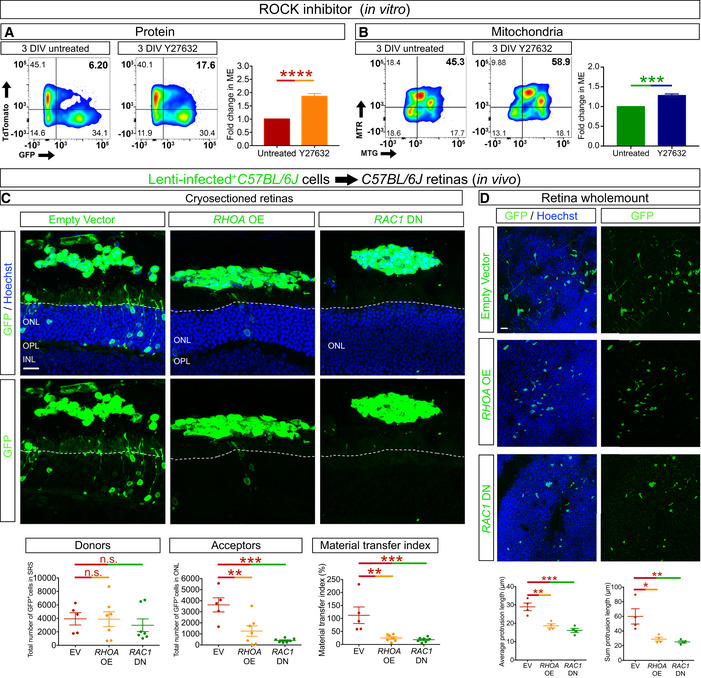
RhoA‐Rac1 plays a role in the extension of photoreceptor protrusions and MT A, BFlow cytometric analysis showing enhancement of material transfer, of protein (biological replicate *n* = 12; technical replicate *n* = 3 per biological replicate) (A) and mitochondria (biological replicate *n* = 3; technical replicate *n* = 3 per biological replicate) (B), in co‐cultures treated with Rho kinase (ROCK) inhibitor Y27632. ME, material exchange.CConfocal microscopy image of cryostat sections of *C57BL/6J* recipient retinas 21 days after transplant of *C57BL/J6* photoreceptors infected with empty vector (*n* = 5), *RHOA* overexpression (*RHOA* OE) (*n* = 7) or *RAC1* dominant negative (*RAC1* DN) (*n* = 7) lentiviruses. Quantification of MT in retinas transplanted with control, RHOA and RAC1 DN lentiviruses.DGFP staining of whole‐mounted retinas after transplantation with control, RHOA and RAC1 DN lentivirus‐infected donor cells (*n* = 4 per group). Quantification shows a significant reduction in the average protrusion length per cell and in the total protrusion length per cell after *RHOA* OE and *RAC1* DN infection compared to the empty vector control. Data information: All data presented as mean ± SEM; n.s. not statistically significant, ****P ≤ 0.0001, ***P < 0.001, **P < 0.01 and *P < 0.05; one‐way ANOVA with Tukey's *post hoc* multiple comparison test and unpaired *t*‐test. White dashed lines delimit the apical side of the ONL of the recipient retina. Scale bars: 50 μm. Source data are available online for this figure.

The formation of membrane protrusions, including neurites, is regulated by the coordinated activity of Rho GTPases, where in general, Rac1 and Cdc42 promote, and Rho inhibits (Faix & Rottner, 2006; Arkwright *et al*, 2010; Ridley, 2011; Chen *et al*, 2012) neurite outgrowth. We have shown previously that inhibiting Rho kinase, a downstream RhoA effector, increases neurite outgrowth in photoreceptor precursors (Tsai *et al*, 2019). Given the similarities between ^Ph^NTs and neurites, we assessed the role of Rho GTPase activity in MT *in vitro* and *in vivo*. Treatment with a Rho kinase inhibitor, Y27632, significantly increased GFP and mitochondria transfer *in vitro* (Fig 8A and B), suggesting a positive role for Rho signalling in MT. To investigate the role of actin remodelling on MT *in vivo*, we infected donor photoreceptors with lentiviruses expressing *RHOA* and dominant negative *RAC1* (*RAC1* DN) (Appendix Fig S3C and D). After confirming that control, *RHOA* and *RAC1* DN lentiviruses induced comparable levels of GFP expression in donor photoreceptors (Appendix Fig S3D) and that *RAC1* DN expression induced growth cone collapse *in vitro* (Appendix Fig S3E), we transplanted these cells into the SRS of wild‐type adult recipients and analysed GFP transfer after 21 days. We confirmed that the presence of GFP in the recipient retina was caused by MT and not integration of the transplanted cells using EdU labelling, which revealed the retention of transplanted donor photoreceptors in the SRS (Appendix Fig S3C). Compared with control lentivirus‐infected cells, *RHOA* and *RAC1* DN expression significantly reduced GFP transfer between donor and acceptor photoreceptors (Fig 8C). This effect was not due to reduced donor photoreceptor viability (Fig 8C) but was associated with a significant reduction in the average and total ^Ph^NT length in *RHOA* and *RAC1* DN expressing donor photoreceptors (Fig 8C and D). We therefore conclude that ^Ph^NT outgrowth in donor photoreceptors is promoted by Rac1 activity and that ^Ph^NT formation is required for MT.

## Discussion

In this report, we investigated the kinetics, cargo types and the cell biological processes that are involved in MT between donor and acceptor photoreceptors *in vivo* and *in vitro*. We find that MT correlates with donor neuron survival *in vivo* and transferred cargo includes protein, mRNA and mitochondria. The sites of GFP exchange between photoreceptors *in vivo* are characterized by the presence of nanotube‐like protrusions, ^Ph^NTs, that connect donor and acceptor cells, and genetic perturbation of the actin cytoskeleton reduces the outgrowth of these protrusions and reduces MT *in vivo*. Moreover, we demonstrate using FRAP that ^Ph^NT protrusions mediate GFP transfer between photoreceptors *in vitro*. Based on these data, we conclude that ^Ph^NTs are conduits for MT *in vivo* and *in vitro*. In a parallel study, Kalargyrou *et al* (2021) describe similar findings, where they show that nanotube‐like protrusions mediate MT between photoreceptors *in vitro* and that Rho GTPase‐dependent cytoskeletal remodelling is required for MT from donor to host photoreceptors *in vivo*. Together these studies make a strong case for protrusion‐based transfer of cytoplasmic contents between photoreceptors.

Nanotubes, referred to originally as tunnelling nanotubes (TNTs), were described as dynamic actin‐containing protrusions that mediate electrical coupling and vesicle transfer between cells (Rustom *et al*, 2004). Subsequently, the definition of nanotubes has expanded to include protrusions with different cytoskeletal content, dimensions and context‐specific functions (Wang *et al*, 2012). TNTs have been described in intact eye structures, including TNTs connecting immune cells in the cornea (Chinnery *et al*, 2008) and inter‐pericyte TNTs in the retinal vasculature that mediate neural vascular coupling (Alarcon‐Martinez *et al*, 2020). However, TNT‐like connections between retinal neurons or glia in the intact retina have not been reported. The transplant‐associated ^Ph^NTs that we describe here share several characteristics with the microtubule^+^ TNTs that connect PC12 cells (Wang & Gerdes, 2015) and cultured primary neurons (Wang *et al*, 2012), including morphology, size, stability after fixation and permissiveness for the exchange of small cytosolic molecules and larger organelles, including mitochondria. However, we cannot rule out an additional role for the classical actin‐only dynamic TNTs in MT in our system. For instance, these types of TNTs could represent an intermediate structure in ^Ph^NT formation or extend from the distal tips of ^Ph^NTs to mediate MT, but as they are fragile, they would not be captured in fixed tissues. What also remains unclear is the mechanism of transfer at the contact sites of these protrusions on acceptor cells. We speculate transfer could involve transient membrane fusions that permit cytoplasmic continuity or the short‐range release and uptake of vesicles. Distinguishing between these mechanisms will require deeper ultrastructural and functional analyses.

The striking parallels between nanotubes and neurites have been noted previously (Nussenzveig, 2019), and several lines of evidence support a neurite‐like identity for the protrusions formed by photoreceptors that we describe here. The photoreceptor precursors used as donor neurons in our *in vivo* and *in vitro* assays are harvested at a stage in development where they extend neurites *in vivo* (Sarin *et al*, 2018) and they exhibit neurite‐extension behaviour in heterotopic environments, including cell culture (Kljavin & Reh, 1991) and after transplantation to the adult retina (Tsai *et al*, 2019). Interestingly, photoreceptors *in vitro* can also form neurites with growth cones and whether these are the precursors to the ^Ph^NT that connect cells remains to be determined. Consistent with this possibility is the demonstration that nanotubes that connect astrocytoma cells *in vivo* exhibit growth cone‐like morphology at their tips (Osswald *et al*, 2015).

Nanotube‐mediated transfer of neural degeneration disease‐causing proteins *in vitro* (Symons & Rusk, 2003) has contributed to the prevailing idea that a similar mechanism could be involved in the connectome‐associated route of disease‐causing protein spread *in vivo* (Ridley, 2006), including the inter‐neuronal spread of misfolded alpha synuclein in Parkinson’s disease and multiple system atrophy and misfolded tau protein in Alzheimer’s disease and frontotemporal dementia (Uemura *et al*, 2020). Our work extends that concept and demonstrates that neuron MT is mediated by a related class of protrusions *in vivo*. Moreover, the signalling mechanisms that control the formation of neurites and membrane protrusions overlap, as both are regulated by Rho GTPase‐dependent actin remodelling (Delage *et al*, 2016; Hanna *et al*, 2017) and non‐canonical Wnt signalling (Sarin *et al*, 2018; Vargas *et al*, 2019). While we found that disrupting RhoA and Rac1 activity inhibits MT, supporting a role for neurite extension in MT, further investigation is needed to examine the role that actin remodelling might play to support cargo trafficking in MT (Symons & Rusk, 2003; Ridley, 2006).

Retinal cell transplantation has been shown to improve visual function in animal models of incomplete photoreceptor degeneration (MacLaren *et al*, 2006; Lamba *et al*, 2009; Tucker *et al*, 2011; Pearson *et al*, 2012; Barber *et al*, 2013; Santos‐Ferreira *et al*, 2015; Wang *et al*, 2016). However, in the case where the host photoreceptor layer is intact, there is no appreciable donor photoreceptor integration; thus, cell replacement is unlikely to mediate functional rescue in this context. MT of proteins that are missing in acceptor photoreceptors has been documented (MacLaren *et al*, 2006; Pearson *et al*, 2012, 2016), which suggests that MT occurs at levels sufficient to complement single gene deficiencies in photoreceptors and restore function (MacLaren *et al*, 2006; Pearson *et al*, 2012). However, MT does not appear to be selective for functional deficiencies, as GNAT1 can transfer to acceptor photoreceptors in *Nrl* ^−/−^ mice (this study), which do not require this protein to function. In addition, we show that several types of cargo can be transferred including cytosolic and nuclear proteins, mRNA and organelles, which raises the question about the selectivity of this process and the function of transferred cargo in acceptor photoreceptors. As shown in this study, MT requires contact between donor and acceptor photoreceptors and thus, if optimized, could represent a therapeutic opportunity for gene complementation and possibly metabolic rescue by mitochondrial transfer. However, successful MT‐based therapies will need to be long‐lasting. Thus, optimizing donor photoreceptor survival, neurite formation and donor and acceptor photoreceptor contact, as well as regulation of cargo selectivity, are important avenues for future investigation.

## Materials and Methods

### Reagents and Tools table

**Table:** 

Reagent/Resource	Reference or Source	Identifier or Catalog Number
**Experimental Models**		
*C57BL/6J* (*M. musculus*)	Jackson Lab	B6.129P2Gpr37tm1Dgen/J. Stock #000664
*Nrl::GFP (M. musculus)*	Akimoto, M. *et al* 2006	
*Nrl* ^−/−^ (*M. musculus*)	Mears, A.J. *et al* 2001	
*ROSA* *^mT/mG^* (*M. musculus*)	Jackson Lab	Gt(ROSA)26Sor^tm4(ACTB‐tdTomato,‐EGFP)Luo^/J. Stock #007576
*Nrl* *^‐/‐^* *; ROSA* *^mT/mG^* (*M. musculus*)	Generated by crossing *Nrl* *^‐/‐^* and *ROSA* *^mT/mG^* (above)	
*Crx::Cre* (*M. musculus*)	Prasov, L. & Glaser, T. 2012	
*Nrl::GFP; Prphr2* *^‐/‐^* (*M. musculus*)	Generated by crossing *Nrl::GFP* and C3A.Cg‐*Pde6b* ^+^ *Prph2* *^Rd2^*/J (Jackson Lab; Stock #001979).	
WERI‐Rb‐1 (*H. sapiens*)		ATCC^®^ HTB‐169™
BT88 (*H. sapiens*)		ATCC^®^ CRL‐3417™
**Recombinant DNA**		
pLenti‐CAG‐P2A‐EGFP (P2A‐GFP) Empty Vector	Derived from pLenti‐CAG‐IRES‐GFP (Addgene #69047). IRES‐GFP was replaced with P2A‐EGFP.	
pLenti‐CAG‐P2A‐EGFP‐nls (P2A‐GFP‐nls) Empty Vector with nls tagged GFP reporter	Derived from pLenti‐CAG‐P2A‐EGFP. Nuclear localization signal (nls) was added at the 3’ end of GFP sequence.	
pLV‐mitoDsRed (mitoDsRed) Mitochondria F1F0‐ATP synthase	Addgene plasmid. A gift from Dr. Pantelis Tsoulfas	Cat #44386
pLenti‐CAG‐RHOA‐P2A‐EGFP (RhoA OE) Human *RHOA* (NM_001664)	Derived from pLenti‐CAG‐P2A‐EGFP. Human RhoA was subcloned into pLenti‐CAG‐P2A‐EGFP in frame with P2A‐EGFP.	
pLenti‐CAG‐RAC1(T17N)‐P2A‐EGFP (RAC1 DN) Human *RAC1‐DN* (NM_006908)	Derived from pLenti‐CAG‐P2A‐EGFP. Human RAC1(T17N) was subcloned into pLenti‐CAG‐P2A‐EGFP in frame with P2A‐EGFP. AC (residues 50‐51) of RAC1 were replaced to CT.	
pMD2.G Enveloping vector	Addgene. A gift from Dr. Didier Trono.	Cat #12259
psPAX2 Packaging vector	Addgene. A gift from Dr. Didier Trono.	Cat #12260
**Antibodies**		
Goat anti‐EGFP (IHF; 1:500) (WB; 1:1000)	Rockland Immunochemicals, Inc. Pottstown, PA, USA	Cat #600‐101‐215
Rabbit anti‐RFP (IHF; 1:500)	Rockland Immunochemicals, Inc. Pottstown, PA, USA	Cat #600‐401‐379
Rabbit anti‐GNAT1 (IHF; 1:200)	Santa Cruz Biotechnology, Inc. Mississauga, ON, Canada	Cat #Sc‐389
Mouse anti‐ATP5B (IHF; 1:500)	Abcam plc. Cambridge, UK	Cat #Ab5432
Mouse anti‐Prph2 (IHF; 1:10 (supernatant))	Gift from Dr. Robert S Molday ref: Hum Mol Genet. 27:295‐306 (2018).	
Mouse anti‐αTubulin (IHF; 1:200)	Santa Cruz Biotechnology, Inc. Mississauga, ON, Canada	Cat #Sc‐53646
Mouse anti‐βTubulin (IHF; 1:100)	Thermo Fisher Scientific, Mississauga, ON, Canada	Cat #MA5‐11740
Mouse anti‐SV2A (IHF; 1:100)	Developmental Studies Hybridoma Bank, University of Iowa, Iowa City, IA, USA	Cat #SV2
Mouse anti‐Bassoon (IHF; 1:5000)	Enzo Life Sciences, Inc., Farmingdale, NY, USA	Cat #ADI‐VAM‐PS003‐D
Rabbit anti‐βTubulin (IHF; 1:100)	Cell Signaling Technologies, Danvers, MA, USA	Cat #2148
Donkey anti‐goat 488 (IHF; 1:500)	Thermo Fisher Scientific, Mississauga, ON, Canada	Cat #A‐11055
Donkey anti‐mouse 555 (IHF; 1:500)	Thermo Fisher Scientific, Mississauga, ON, Canada	Cat #A‐31570
Donkey anti‐mouse 647 (IHF; 1:500)	Thermo Fisher Scientific, Mississauga, ON, Canada	Cat #A‐31571
Donkey anti‐rabbit 555 (IHF; 1:500)	Thermo Fisher Scientific, Mississauga, ON, Canada	Cat #A‐31572
Donkey anti‐rabbit 647 (IHF; 1:500)	Thermo Fisher Scientific, Mississauga, ON, Canada	Cat #A‐31573
Rabbit anti‐βTubulin (WB; 1:1000)	Abcam plc. Cambridge, UK	Cat #Ab6046
Mouse anti‐Flotilin1 (WB; 1:800)	BD Transduction Laboratories^TM^, San Jose, CA, USA	Cat #610821
Mouse anti‐GAPDH (WB; 1:10000)	Calbiochem, Merck Millipore Sigma Aldrich, Oakville, ON, Canada	Cat #CB1001
Rabbit anti‐RhoA (WB; 1:1000) (IHF; 1:500)	Cell Signaling Technologies, Danvers, MA, USA	Cat #2117
Mouse anti‐Rac1 (WB; 1:1000) (IHF; 1:500)	Merck Millipore Sigma Aldrich, Oakville, ON, Canada	Cat #05‐389
Donkey anti‐goat IRDye 800CW (WB; 1:10000)	LI‐COR Biosciences, Lincoln, NE, USA	Cat #926‐32214
Donkey anti‐goat IRDye 680RD (WB; 1:10000)	LI‐COR Biosciences, Lincoln, NE, USA	Cat #926‐68074
Donkey anti‐mouse IRDye 800CW (WB; 1:10000)	LI‐COR Biosciences, Lincoln, NE, USA	Cat #926‐32212
Donkey anti‐mouse IRDye 680RD (WB; 1:10000)	LI‐COR Biosciences, Lincoln, NE, USA	Cat #926‐68072
Donkey anti‐rabbit IRDye 800CW (WB; 1:10000)	LI‐COR Biosciences, Lincoln, NE, USA	Cat #926‐32213
Donkey anti‐rabbit IRDye 680RD (WB; 1:10000)	LI‐COR Biosciences, Lincoln, NE, USA	Cat #926‐68073
**Oligonucleotides and sequence‐based reagents**		
Genotyping primers	This study	Table EV2
**Chemicals, enzymes and other reagents**		
Cyclosporin	Merck Millipore Sigma Aldrich, Oakville, ON, Canada	Cat #C3662‐5MG
GW4869	Merck Millipore Sigma Aldrich, Oakville, ON, Canada	Cat #D1692‐5MG
Chloroquine	Merck Millipore Sigma Aldrich, Oakville, ON, Canada	Cat #PHR1258‐1G
Amitriptyline	Merck Millipore Sigma Aldrich, Oakville, ON, Canada	Cat #A8404‐10G
Y27632 ROCK inhibitor	Cell Signaling Technologies, Danvers, MA, USA	Cat #13624S
5‐ethynyl‐2'‐deoxyuridine	Thermo Fisher Scientific, Mississauga, ON, Canada	Cat #C10340
**Software**		
Imaris 9.0.2	Bitplane Inc. Zurich, Switzerland	
InVivoVue Clinic 1.2 QOP‐60‐05 revision C	Bioptigen, Leica Microsystems NC, Inc (formerly Bioptigen)	
FACSDiva 8.0.1	BD Biosciences, San Jose, CA, USA	
FlowJo v10.7.1	BD Biosciences, San Jose, CA, USA	
**Other**		
LSM 780 confocal microscope	Carl Zeiss Inc. Thornwood, NY, USA	
Leica TCS SP8 multiphoton microscope	Leica Microsystems, Wetzlar, Germany	
Ultramicroscope II light sheet microscope	LaVision BioTec GmbH, Bielefeld, Germany	
Spectral‐domain optical coherence tomography (SD‐OCT)	Bioptigen; Morrisville, NC, USA. Leica Microsystems NC, Inc (formerly Bioptigen)	
FEI Tecnai G2 Spirit transmission electron microscope	TEM; FEI Europe, Eindhoven, The Netherlands	
Aria III	BD Biosciences, Mississauga, ON, Canada	

### Methods and Protocols

#### Animals and genotyping

Animal husbandry was in accordance with the Association for Research in Vision and Ophthalmology (ARVO) Statement for the Use of Animals in Ophthalmic and Vision Research. Animals were maintained under standard laboratory conditions, and all procedures were performed in conformity with the University Health Network Animal Care Committee (protocol 3499.17) and adhered to the guidelines of the Canadian Council on Animal Care. Mouse strains are summarized in Appendix Table S1. Adult (6–10 weeks old) *C57BL/6J*, *Nrl* ^−/−^, *ROSA^mT/mG^* and *Nrl* ^−/−^ *; ROSA^mT/mG^* mice of both sexes were used as transplantation recipients. *C57BL/6J*, *Nrl::GFP*, *Crx::Cre* and *ROSA^mT/mG^* mice at P0‐P5 were used to harvest retinal tissue. Genotyping was performed by extracting genomic DNA from ear clip samples through incubation in 200 μl alkaline lysis buffer (25 mM NaOH S8263‐150ML, Merck Millipore Sigma‐Aldrich, Oakville, ON, Canada) 0.2 mM EDTA pH 8.0 (EDT001.500 BioShop Canada Inc. Burlington, ON, Canada) for 1 h at 95°C. Samples were neutralized with 200 μl neutralization buffer (40 mM Tris–HCl) (15504020, Thermo Fisher Scientific, Mississauga, ON, Canada) and genotyped by polymerase chain reaction (PCR) using primer sets indicated in Appendix Table S2.

#### Cell preparation and culture

##### Cell lines

WERI‐Rb‐1, a human retinoblastoma cancer cell line, was maintained in RPMI‐1640 (350‐000‐CL, Winset Inc., Montreal, QC, Canada) supplemented with 10% heat‐inactivated foetal bovine serum (12483020, Thermo Fisher Scientific, Mississauga, ON, Canada) and penicillin/streptomycin (0.1%) (15140122, Thermo Fisher Scientific, Mississauga, ON, Canada). BT088, a human glioma cancer cell line (Kelly *et al*, 2010), was maintained in Human Neurocult NS‐A proliferation kits (05751, STEMCELL Technologies, Vancouver, BC, Canada) supplemented with epidermal growth factor (hEGF, 20 ng/ml) (E9644‐2MG, Merck Millipore Sigma‐Aldrich, Oakville, ON, Canada), fibroblast growth factor (hFGF2, 20 ng/ml) (233‐FB‐025, R&D Systems Inc. Minneapolis, MN, USA), heparin solution (2 μg/ml) (07980, STEMCELL Technologies, Vancouver, BC, Canada), penicillin/streptomycin (0.1%) and amphotericin B (40 ng/ml) (15290026, Thermo Fisher Scientific, Mississauga, ON, Canada) – SFM‐EF. BT088 cells were passaged every three days with SFM‐EF, dissociated with ACCUTASETM (07920, STEMCELL Technologies, Vancouver, BC, Canada) and resuspended in SFM‐EF.

##### Primary cells

For retinal dissociates, P0‐P2 pups from *C57BL/6J* (for lentivirus infection, see below) or P3‐P5 pups from *Nrl::GFP*, *Nrl* ^−/−^;*Ccdc136* ^GFP/GFP^, *Crx::Cre* and *ROSA^mT/mG^* mice were euthanized by decapitation after a previous exposure to carbon dioxide inhalation for 5 min. Retinas were dissected and collected in CO_2_‐independent media (18045088, Thermo Fisher Scientific, Mississauga, ON, Canada). For cortical cells, pregnant *C57BL/6J* adult mice were euthanized by carbon dioxide inhalation. Embryos (E18) were extracted from the amniotic sac and euthanized by decapitation. Brains were dissected from skull, the lobes were separated in a clean dish, and the meninges were removed. Using a third clean dish, the cortices were dissected and collected in CO_2_‐independent media.

##### Tissue dissociation

Retinal and cortical tissues were dissociated to single cells with papain (LK003150, Worthington Biochemical Corp. Lakewood, NJ, USA) according to the manufacturer’s directions. Cells were then washed in Ca^2+^/Mg^2+^‐free phosphate‐buffered saline (PBS) (D8537‐500ML, Merck Millipore Sigma‐Aldrich, Oakville, ON, Canada) and counted using a haemocytometer after staining with a 0.4% Trypan blue viability stain (15250061, Thermo Fisher Scientific, Mississauga, ON, Canada). Cells were then passed through a cell strainer (40 μm) (CLS431750, Thermo Fisher Scientific, Mississauga, ON, Canada) before being resuspended in retinal explant media (REM) (Appendix Table S3) supplemented with SATOs (Appendix Table S4) for cell culture or in 0.005% DNAse (D5025‐150KU, Merck Millipore Sigma‐Aldrich, Oakville, ON, Canada) in Earl's Balanced Salt Solution (EBSS) (14155063, Thermo Fisher Scientific, Mississauga, ON, Canada) and kept on ice prior to transplantation.

##### Primary retinal cell co‐culture

P3‐5 *Nrl::GFP* and *ROSA^mT/mG^* cells were seeded at a 1:1 ratio to a final density of 2 × 10^5^ cells in 200 μl of REM per well on plastic‐bottomed 96‐well plates (83.3924.500, Sarstedt Inc. Saint‐Léonard, QC) or glass‐bottomed 96‐well plates suitable for confocal imaging (P96G‐1.5‐5‐F, Mat‐Tek Cop. Ashland, MA, USA) and maintained in 5% CO_2_‐buffered incubators at 37°C. Half of the REM was replenished after 3 and 5 days *in vitro* (DIV). To label mitochondria, dissociated retinal cells from *C57BL/6J* mice were labelled with 50 nM MitoTracker™ Green FM (MTG) (M7514, Thermo Fisher Scientific, Mississauga, ON, Canada) or 100 nM MitoTracker™ Red FM (MTR) (M22425, Thermo Fisher Scientific, Mississauga, ON, Canada) for 20 min at 37°C in 2 ml of REM. The cells were then washed in Dulbecco’s modified Eagle media (DMEM) (D5796‐500ML, Merck Millipore Sigma‐Aldrich, Oakville, ON, Canada) three times followed by a final wash in REM. MTG‐ and MTR‐labelled retina cells were then co‐cultured at a 1:1 ratio at a final density of 2 × 10^5^ cells in 200 μl of media per well on plastic‐ or glass‐bottomed 96‐well plates, as described above. For transwell experiments, 1 × 10^6^ of one fluorescent population of retinal dissociates was cultured on 0.4 μm polycarbonate (PC)‐membrane permeable inserts (10769‐208, VWR International, LLC. Mississauga, ON, Canada) and 1 × 10^6^ of a second fluorescent population on plastic‐bottomed 12‐well plates.

#### EdU labelling

To label photoreceptor nuclei, P1 *C57BL/6J* pups received three intraperitoneal injections every 6 h with 5‐ethynyl‐2'‐deoxyuridine (50 mg/kg, 5 mg/ml stock concentration) (Click‐iT^®^ EdU Labelling kit, OCT Thermo Fisher Scientific, Mississauga, ON, Canada) and retinas were harvested at P3. EdU labelling was developed using an Alexa Fluor 647 Click‐iT (azides/alkyne) reaction, as per the manufacturer's instructions, and was confirmed at ≥ 60% of post‐mitotic donor cells.

#### Fluorescence‐activated cell sorting of photoreceptors

Cells were sorted for CD73‐APC (127210, BioLegend, San Diego, CA, USA) photoreceptor surface receptor expression and GFP fluorescence using an Aria III (BD Biosciences, Mississauga, ON, Canada) equipped with 633‐nm and 488‐nm lasers. Post‐sort, cells were collected in 10% bovine serum albumin (BSA) (A7030‐100G, Merck Millipore Sigma‐Aldrich, Oakville, ON, Canada) in Ca^2+^/Mg^2+^‐free PBS. The sort was performed using an 85 μm nozzle at 45 psi and a flow rate that allowed collection at 5000 events per second. The data acquisition, analysis and image preparation were carried out using the instrument software FACSDiva (BD Biosciences, Mississauga, ON, Canada). The gating histograms are shown in Appendix Fig S1B and C. Sorted cells were resuspended at a concentration of 2 × 10^6^ cells/ml in REM (Appendix Tables S3 and S4) for cell culture.

#### Lentiviral infection

Retinal dissociates from P0‐2 mice were resuspended in REM (Appendix Tables S3 and S4) and plated at a concentration of 3 × 10^7^ cells in 10 ml of REM into 10 cm Primaria™ dishes (353801, BD Falcon, BD Biosciences, Oakville, ON, Canada). Lentiviruses were added to the plate at a titre to achieve > 80% infection efficiency. All cultures were maintained in 5% CO_2_‐buffered incubators (50116048, Heracell™ 150i CO_2_, Thermo Fisher Scientific, Asheville, NC, USA) at 37°C. After 2 * *DIV, cells were de‐adhered with Trypsin‐EDTA (0.05% Trypsin with EDTA 4Na) (25300062, Thermo Fisher Scientific, Mississauga, ON, Canada) for 5 min and the enzymatic digestion was stopped with 10% BSA. Cells were washed in Ca^2+^/Mg^2+^ ‐free PBS four times and filtered through a 40‐μm cell strainer. To confirm that GFP expression was not the result of lentivirus carryover in donor cell preparation, for every transplantation experiment we injected 2 μl of the final cell wash to the SRS in three different animals and analysed GFP expression after 21 days. Cells were resuspended at a concentration of 200,000 cells per μl in 0.005% DNAse in EBSS and kept on ice prior to transplantation or cell culture.

#### Lentivirus design, cloning and production

A list of all plasmids and expression vectors used in this study is provided in Appendix Table S5. Synthetic complementary DNAs (cDNAs) of human *RHOA* and dominant negative human *RAC1*, *RAC1(T17N)*, were synthesized using NCBI gene database as references (NM_001664 & NM_006908, respectively) (National Center for Biotechnology Information (NCBI). Bethesda (MD): National Library of Medicine (US), National Center for Biotechnology Information; [1988] – [cited 2017 Apr 06]. Available from: https://www.ncbi.nlm.nih.gov/) and gene synthesis service from Bio Basic Inc. Markham, ON, Canada. *RHOA* and *RAC1(T17N)* cDNAs were subcloned into the pLenti‐CAG‐P2A‐EGFP vector backbone (derived from pLenti‐CAG‐IRES‐GFP, Addgene #69047; Lu *et al*, 2014) in frame with P2A‐EGFP. All constructs were verified by Western blot. Lentiviruses were produced by Lipofectamine3000 (L3000015, Invitrogen Canada Inc. Burlington, ON, Canada) mediated transfection of transfer plasmid with 2^nd^ generation lentiviral packaging system (pMD2.G and psPAX2) into 293FT cells (ATCC; CRL‐1573™). Supernatant was collected 48 h post‐transfection and concentrated by centrifugation at 100,000 *g* for 3 h. The lentivirus was resuspended at approximately 1/150 of its original volume in either PBS (D8662‐500ML, Merck Millipore Sigma‐Aldrich, Oakville, ON, Canada) or DMEM (D5796‐500ML, Merck Millipore Sigma‐Aldrich, Oakville, ON, Canada) and then frozen at −80°C. Virus titre was determined by the frequency of GFP or RFP expression in HEK293T cells.

#### Subretinal injection

Dissociated cells were resuspended at a concentration of 50,000 (human tumour cell lines and cortical primary neurons) or 200,000 (primary retinal cells) cells per μl in 0.005% DNAse in EBSS and kept on ice prior to transplantation. Adult recipient *C57BL/6J* and *Nrl* ^−/−^ mice (6–8 weeks old) were anaesthetized using a mixture of ketamine (100 mg/ml, Ketalean) (8KET004D, Bimeda MTC Animal Health Inc. Cambridge, ON, Canada) at 50 mg/kg and medetomidine (1 mg/ml, Cepetor) (236 1506 0, Modern Veterinary Therapeutics LLC, Miami, FL, USA) at 1 mg/kg in sterile 0.9% NaCl (JB1300, Baxter Corp. Mississauga, ON, Canada) administered intraperitoneally. Pupils were dilated using 1% tropicamide (Mydriacyl) (0065‐0355‐03 Alcon, Mississauga, ON, Canada) drops, followed by application of a 0.2% hypromellose gel (Genteal Tears) (0078‐0429‐57, Alcon, Mississauga, ON, Canada) to maintain proper eye lubrication. For injections, the left eye was gently prolapsed and then immobilized using a customized latex dam to allow free blood circulation. The retina fundus was visualized under a dissection microscope, adjusting the reflexion index, for confirmation of cell deposit. A microscopy‐guided microinjection was performed by a second surgeon located orthogonal to the viewing microscope. A scleral incision was made in the dorsal side, posterior to the limbus using a 30‐gauge needle (305136, VWR International, LLC. Mississauga, ON, Canada). Next, a blunt 32‐gauge needle (7762‐05, Hamilton Company, Montreal, QC, Canada) was inserted tangentially into the SRS and advanced under the guidance of the person visualizing the fundus. Once the needle was located in the SRS, a small incision was made in the cornea to relieve intraocular pressure. 1.0 μl of cell suspension (dose varying between 50,000 and 200,000 donor cells) was injected over 30 s using a Remote Infuse/Withdraw Pump 11 Elite Nanomite Programmable Syringe Pump (70‐4507, Harvard Apparatus, Saint Laurent, QC, Canada) connected to a Compact Mouse and Rat Stereotaxic Instrument, Dual Manipulator (75‐1827, Harvard Apparatus, Saint Laurent, QC, Canada). After a 60 s pause to allow for equilibration of pressure and to prevent efflux, the needle was then slowly retracted, and the animal anaesthesia reversed using an intraperitoneal injection of 1 mg/kg atipamezole (5 mg/ml Revertor) (236 1504 0, Modern Veterinary Therapeutics LLC, Miami, FL, USA). Animals were placed on a heating pad and monitored until fully recovered.

#### Drug administration

All mice transplanted with human cancer cells were treated with oral cyclosporin (C3662‐5MG, Merck Millipore Sigma‐Aldrich, Oakville, ON, Canada) *ad libitum* (50 µg/g/day in drinking water) beginning 3 days before transplantation until the experimental endpoint. To inhibit extracellular vesicle secretion *in vivo* beginning one day before transplantation, animals were given intraperitoneal injections of the following compounds: GW4869 (D1692‐5MG, Merck Millipore Sigma‐Aldrich, Oakville, ON, Canada), a neutral sphingomyelinase inhibitor, 2 mg/kg body weight, every 3 days; chloroquine (PHR1258‐1G, Merck Millipore Sigma‐Aldrich, Oakville, ON, Canada), an autophagy and EV fusion inhibitor, 5 mg/kg body weight, daily; and amitriptyline (A8404‐10G, Merck Millipore Sigma‐Aldrich, Oakville, ON, Canada), an inhibitor of ectosome secretion, 10 mg/kg body weight, daily. As a control, we injected a fourth group of animals with 0.9% NaCl (vehicle) daily. For *in vitro* experiments, culture medium was supplemented with GW4869 (20 μM diluted in dimethyl sulfoxide (DMSO) (276855‐100ML, Merck Millipore Sigma‐Aldrich, Oakville, ON, Canada)), chloroquine (500 nM diluted in REM) and amitriptyline (500 nM diluted in REM). REM and REM‐DMSO (same amount) were used as controls. Controls were run on the same days as their respective experimental treatments. To enhance neurite outgrowth *in vitro*, cultures were supplemented with Y27632 ROCK inhibitor (13624S, Cell Signaling Technologies, Danvers, MA, USA) (100 μM diluted in REM).

#### Optical coherence tomography imaging

Resolution of the retinal detachment induced by the subretinal cell delivery was monitored over time using a spectral‐domain optical coherence tomography (SD‐OCT) device (Bioptigen; Morrisville, NC, USA). Briefly, mice were anaesthetized and analysed using SD‐OCT before, immediately after, and at 5, 9 and 21 days post‐transplantation. Animals were excluded from the study if any complications affecting the opacity of the cornea or lens were observed. Retinal detachment was measured in SD‐OCT images using the area of maximum length between the retina and the retinal pigmented epithelium, using callipers provided directly by the software (InVivoVue Clinic 1.2 QOP‐60‐05 revision C). The measurements correspond to the area of larger detachment in each eye that, consequently, is the area of maximum delivery of the transplanted cells during the injections.

#### Flow cytometric analysis

To prepare co‐cultures for flow cytometric analysis, cells were detached from the wells with 100 μl of TrypLE (12604021, Thermo Fisher Scientific, Mississauga, ON, Canada) for 10 min at 37°C, followed by gentle trituration. Cell suspensions were centrifuged for 5 min at 1300 RPM and resuspended in 2% BSA and 0.005% DNase. To stain photoreceptors, cells were incubated with CD73‐APC (0.05 μg/μl) or CD73‐PE (127206, BioLegend, San Diego, CA, USA) (0.02μg/μl) for 20 min and then incubated with eFluor780 (501129035 Invitrogen, Thermo Fisher Scientific, Mississauga, ON, Canada) viability marker, 1:5 dilution for 10 min at room temperature (RT). Cells were then centrifuged and fixed in 4% paraformaldehyde aqueous solution (PFA) for 10 min followed by 1:1 addition of 10% BSA. Fixed cells were centrifuged and resuspended in Ca^2+^/Mg^2+^‐free PBS and stored at 4°C until analysis. Analysis was performed on BD LSRFortessa X20 (5 laser) and LSRFortessa (4 laser) with high‐throughput sampler option (HTS) using FACSDiva software.

#### Isolation and characterization of extracellular vesicles

Extracellular vesicles were isolated from *Nrl::GFP* retinal dissociates harvested at P4; the retinas from six animals were pooled, dissociated with papain to obtain a single cell suspension and cultured for 24 h in REM at a density of 6 × 10^6^ cells/ml in a total volume of 10 ml, in a controlled environment (37°C incubator, 5% CO_2_). EVs were purified from the culture supernatant using a sequential centrifugation procedure described previously (Thery *et al*, 2006). In brief, the culture supernatant was centrifuged at 300 *g* for 10 min to remove the cells. The cell‐free supernatant was then centrifuged at 2000 *g* for 10 min and then at 10,000 *g* for 30 min to remove dead cells and cell debris, respectively. The resulting supernatant was centrifuged at 100,000 *g* for 8 h at 4°C (Beckman Type 90 Ti). The EV pellet was washed in PBS and subjected to another 100,000 *g* centrifugation for 1 h at 4°C. The supernatant was discarded, and the pellet obtained was resuspended in 50 μl PBS and stored at −80°C.

#### Tissue processing

Mice were euthanized by carbon dioxide inhalation and transcardially perfused with PBS for exsanguination and 4% PFA for fixation. Eyes were then marked with a silver nitrate stick (118‐395, AMG Medical Inc. Mont‐Royal, QC, Canada) on the dorsal region while maintaining the caruncle as an anatomical spatial reference. For cryosectioning, tissues were fixed for an additional 30 min in 4% PFA on ice and then cryoprotected overnight in 30% sucrose (SUC507.1, BioShop Canada Inc. Burlington, ON, Canada) PBS solution at 4°C. Tissues were equilibrated in 50:50 30% sucrose in PBS:OCT (Tissue‐Tek^®^) (4583, Sakura Finetek USA Inc. Maumee, OH, USA) for 1 h and then oriented and embedded in plastic moulds. Tissue blocks were stored at −80°C. Tissue was sectioned at 20 μm thickness onto Superfrost Plus slides (12‐550‐15 Thermo Fisher Scientific, Mississauga, ON, Canada) on a cryostat (CM3050 S, Leica Biosystems, Leica Microsystems Canada Inc. Richmond Hill, ON, Canada) and air‐dried for 1 h before being stored in a slide box with desiccant at −20°C. For retina whole mounts, tissues were flattened by making four radial cuts (the deepest one in the dorsal pole), post‐fixed for an additional hour in 4% PFA and kept in PBS until immunostaining. For tissue clearing, for intact PFA‐fixed eyes were transferred to 4% PFA (157‐4‐100, Electron Microscopy Sciences, Hatfield, PA, USA) for 1 h at RT followed by clearing using a modified CUBIC protocol (Nojima *et al*, 2017) where the incubation times were optimized for the size of the rodent eye. Eyes were incubated in 50% CUBIC‐1/H_2_O for 2 h at 37°C before an overnight incubation in CUBIC‐1 at 37°C followed by a wash in PBS for 2 h at RT. Eyes were then incubated in 50% CUBIC‐2/PBS for 2 h at 37°C before an overnight incubation in CUBIC‐2 at 37°C. Excess CUBIC‐2 was removed using a Kimwipe, and samples were washed briefly in Type FF immersion oil (16212, Cargille Laboratories Inc. Cedar Grove, NJ, USA) for 10 min at RT before imaging. Whole‐mounted retinas were incubated in CUBIC‐1 for 5 min at RT before of a wash for 5 min at RT in PBS followed by a 10‐min incubation in CUBIC‐2 at RT, followed by removal of the excess CUBIC‐2 solution and mounting in Type FF immersion oil.

#### Immunohistochemistry (IHC)

Cell cultures were fixed by adding 4% PFA to the wells for 10 min followed by three washes with PBS. Fixed cultures were blocked in 10% donkey serum (DS) (D9663‐10ML, Merck Millipore Sigma‐Aldrich, Oakville, ON, Canada) in 0.5% Triton‐X (Tx) (X100, T9284‐500ML, Merck Millipore Sigma‐Aldrich, Oakville, ON, Canada) in PBS for 1 h at RT followed by incubation with primary antibodies (Appendix Table S6) in 5% DS and 0.25% Tx in PBS overnight at 4°C. Cultures were washed three times with PBS and incubated with secondary antibodies (Appendix Table S6) in PBS for 1.5 h at RT in a light‐protected box. Cell nuclei were counterstained with Hoechst 33342 (62249, Thermo Fisher Scientific, Mississauga, ON, Canada) diluted in PBS at a concentration of 1:15,000 for 20 min at RT. Fixed cultures were then washed a final time with PBS in a light‐protected box. To stain for F‐actin, 1:40 Alexa Fluor 647 Phalloidin (A22287, Thermo Fisher Scientific, Mississauga, ON, Canada) was included in the secondary antibody incubation step. To live‐image actin or microtubules, cultures were supplemented overnight with SiR‐Actin (100 μM) (CY‐SC001, Cytoskeleton Inc. Denver, CO, USA) or ViaFluor^®^ (50 μM) (70062, Biotium, Fremont, CA, USA). Retina cryosections were permeabilized with PBS 0.5% Tx and then blocked with 2% DS 0.5% Tx in PBS. Slides were incubated with primary antibodies (Appendix Table S6) diluted in 2% DS 0.5% Tx in PBS overnight at 4°C in a light protected humidified box. After three washes with PBS, sections were incubated with fluorescent secondary antibodies diluted in 0.5% Tx in PBS for 2 h at RT and nuclei and F‐actin staining was performed, as described above. Slides were washed and glass coverslips were mounted with DAKO mounting media (S3023, Cedarlane, Burlington, ON, Canada). To stain retina whole mounts, tissue was permeabilized by incubation with 2% Tx in PBS for 1 h at RT and then incubated with primary antibodies (Appendix Table S6) diluted in blocking buffer (PBS, 2% DS, 2%Tx) overnight at 4°C in light protected 1.5‐ml tubes (AD151‐N500, Diamed Lab Supplies Inc. Mississauga, ON, Canada). Retinas were washed three times in PBS 2%Tx and incubated for 3 h at RT with secondary antibodies diluted in PBS 2%Tx. Nuclei were counterstained with fluorescent DNA‐binding dye, Hoechst 33342 (H3570, Life Technologies Thermo Fisher Scientific, Mississauga, ON, Canada) and retinas were mounted in DAKO mounting media, vitreal side down.

#### Fluorescence *in situ* hybridization

Samples were prepared in accordance with the Advanced Cell Diagnostics' (ACD) protocol for RNAscope^®^ Fluorescent Multiplex assay protocol. Samples were pretreated with the RNAscope^®^ Target retrieval (322001, ACD, Newark, CA, USA) at 100°C for 5 min and incubated RNAscope^®^ Protease III (322337, ACD, Newark, CA, USA) for 30 min at 40°C, followed by hybridization and amplification steps according to the RNAscope protocol provided by ACD. Briefly, RNAscope^®^ Probe ‐ EGFP‐O4 (538851, ACD, Newark, CA, USA) was hybridized for 2 h at 40°C in a HybEZ™ II oven (321721, ACD, Newark, CA, USA). Slides were washed with 1X wash buffer two times 2 min each at RT following each amplification step using Amp4 Alt B‐FL detection reagents. The IHF protocol was performed as described above for cryosections.

#### Immunoblotting

Total protein extracts were prepared from frozen adult mouse retinas or cell cultures by manual homogenization in ice‐cold RIPA lysis buffer (50 mM Tris–HCl (10812846001, Merck Millipore Sigma‐Aldrich, Oakville, ON, Canada) pH 7.4, 150 mM NaCl (567440, Merck Millipore Sigma‐Aldrich, Oakville, ON, Canada), 1% NP40 (492016, Merck Millipore Sigma‐Aldrich, Oakville, ON, Canada), 0.1% SDS (428018‐200ML, Merck Millipore Sigma‐Aldrich, Oakville, ON, Canada), 0.5% sodium deoxycholate (30970, Merck Millipore Sigma‐Aldrich, Oakville, ON, Canada), 10 mM NaF (S7920‐100G, Merck Millipore Sigma‐Aldrich, Oakville, ON, Canada), 5 mM sodium citrate (80552, Merck Millipore Sigma‐Aldrich, Oakville, ON, Canada), 1.5 mM MgCl_2_ (M8266‐100G, Merck Millipore Sigma‐Aldrich, Oakville, ON, Canada) and 10 μM ZnCl_2_ (39059‐1ML‐F, Merck Millipore Sigma‐Aldrich, Oakville, ON, Canada)) supplemented with a protease inhibitor cocktail (11836170001, Roche, Merck Millipore Sigma‐Aldrich, Oakville, ON, Canada) and phosphatase inhibitor cocktail (5870S, Cell Signaling Technologies, Danvers, MA, USA). Homogenates were sonicated for 10 s at 4°C, followed by centrifugation at 15,000 *g* for 20 min at 4°C to sediment insoluble material. Protein concentration was determined with Bradford reagent (B6916‐500ML, Merck Millipore Sigma‐Aldrich, Oakville, ON, Canada), and samples were resolved by denaturing SDS–PAGE on 4–20% Tris‐acetate polyacrylamide gels (4561094, Bio‐Rad Laboratories (Canada) Ltd. Mississauga, ON, Canada) and blotted onto PVDF membranes (1620177, Bio‐Rad Laboratories (Canada) Ltd. Mississauga, ON, Canada) by electrophoretic transfer in Tris‐glycine buffer containing 10% methanol (6701‐7‐40, Caledon Laboratories Ltd. Georgetown, ON, Canada) and 0.05% SDS. Membranes were blocked with 5% BSA in Tris‐buffered saline (TBS), followed by an incubation for 1h at RT and overnight at 4°C with primary antibodies, and a subsequent incubation for 2 h at RT with secondary antibodies (Appendix Table S7). Membranes were subjected to three 10‐min washes in TBS following primary and secondary antibody incubation.

#### Imaging

##### Confocal imaging

Cell preparations, cryostat tissue sections and whole‐mounted retinas were imaged using an LSM 780 (Carl Zeiss Inc. Thornwood, NY, USA) confocal microscope. Images were acquired at a minimum of 2,048 × 2,048 pixels resolution at a maximum of 5% laser intensity and a maximum 800 digital gain. 20×, 40× water immersion and 63× oil immersion objectives were used at a 1.0 Airy Unit pinhole size. Comparative images were taken using identical acquisition parameters. Live imaging was performed in a 37°C, 5% CO_2_ equilibrated chamber. Images were acquired at intervals varying from 1 s to 5 min. Intact cleared eyes were imaged using the Ultramicroscope II light sheet microscope (LaVision BioTec). Either an Olympus MVPLAPO 2× dry lens equipped with a LaVision BioTec solvent dipping cap or a Leica HCX APO L20×/0.95 IMM20x solvent‐compatible objective lens connected to an infinity‐corrected zoom body (LaVision BioTec GmbH, Bielefeld, Germany) was dipped into the oil mounting medium (16212, Cargille Laboratories Inc. Cedar Grove, NJ, USA) and focused on the illuminated light sheet plane. A numerical aperture of 0.154 was used with a sheet thickness of 5 µm. Image acquisition parameters were maintained for all comparative images. For two‐photon imaging, a custom 3D printed, two chamber specimen‐holder was designed to allow for cleared tissue samples to be imaged using a Leica HCX PL APO L 20×/1.0 W water immersion lens equipped with a motorized correction collar. Briefly, intact eyes are immersed in imaging oil and immobilized in a lower, sealed specimen chamber that is capped by a #1, 22 × 22 mm coverslip (16004‐094, VWR International, LLC. Mississauga, ON, Canada), which separates the lower and upper chambers. An upper chamber is added that is filled with distilled water for use with the Leica HCX PL APO L 20×/1.0 W water immersion lens. Whole eyes were imaged on a dual beam Leica TCS SP8 multiphoton microscope (Leica Microsystems, Wetzlar, Germany) equipped with a 1,300 nm Chameleon Discovery laser (Coherent, Santa Clara CA, USA). For GFP imaging, the laser was tuned to 960 nm. TdTomato was excited using a fixed‐beam laser at 1,040 nm. Power output for GFP and tdTomato channels was maintained at 45 mW. Emitted light from all fluorophores was captured using an internal HyD SP GaAsP hybrid detector gated to a range of 500–525 nm for GFP, 550–590 for tdTomato. Z‐stack images were acquired with 2× accumulation, 2× averaging, 0.70 µm z‐resolution and a minimum of 960 × 960 xy resolution. Image acquisition parameters were maintained for all comparative images.

##### Electron microscopy

For electron microscopy, a 10‐μl aliquot of freshly isolated exovesicles were loaded onto Formvar carbon‐coated grids and negatively stained with 2% uranyl acetate. The grids were examined with a FEI Tecnai G2 Spirit transmission electron microscope (TEM; FEI Europe, Eindhoven, The Netherlands), and the images were recorded using a Morada CCD camera (Olympus Soft Image Solutions GmbH, Münster, Germany).

##### Fluorescence recovery after photobleaching (FRAP)

To analyse protein dynamics *in vitro*, we performed FRAP analysis in cultures of *Nrl::GFP* retinal dissociates at 2 DIV. Using an LSM 780 (Carl Zeiss Inc. Thornwood, NY, USA) confocal microscope, images were acquired with a 63×/1.40 Oil DIC M27 objective, at a resolution of 512 × 512 and a pixel dwell of 1.27 µs. For each trial, cells were imaged at 1‐s intervals five times to determine the initial fluorescence intensity for each region of interest (ROI). Cells were photobleached using 100% laser power applied in ROIs with 21 interactions over 12 s. Immediately after photobleaching, cells were imaged at 1‐s intervals over a total interval of 244 s. The fluorescence intensity in arbitrary units (a.u.) was collected from ROIs, processed in Microsoft Excel (Office Excel, version 16.35, 2020; Microsoft Corp., Redmond, WA, USA) and analysed using GraphPad Prism version 7.0a (GraphPad Software Inc. La Jolla, CA, USA). To avoid including the distal tips of the protrusions in the measurement of fluorescence recovery, the ROI registered to monitor fluorescence recovery in the photobleached cell was smaller than the ROI used for photobleaching. Intensity values were normalized to the mean pre‐photobleaching fluorescence intensity and the intensity value immediately post‐photobleaching was used to normalize and represent all post‐photobleaching values in terms of fluorescence recovery in each ROI. Statistical analyses were performed with an extra‐sum‐of‐squares F‐test (Y0 constrained to 0.0) after fitting the curve in a non‐linear regression with two‐phase association.

##### Image processing

Two‐photon and light sheet fluorescence microscopy images were post‐processed in Imaris 9.0.2 (Bitplane Inc. Zurich, Switzerland). When appropriate, 3D images were processed using a background subtraction, followed by a normalization algorithm before making adjustments to brightness and contrast in Surpass mode. Semi‐automatic quantification of neurite length was performed using the Filaments module in Imaris 9.0.2 to trace GFP^+^ protrusions. Neurites less than 5 µm in length were excluded from data analysis.

#### Quantification

##### Quantification of Material Transfer index

The quantification of GFP^+^ cells in transplanted retinas was performed, as previously described. Briefly, the number of GFP^+^ cell bodies in the recipient outer nuclear layer (ONL) and the number of GFP^+^ cells in the adjacent SRS were counted. The total number of GFP^+^ cells per eye was determined by extrapolation, based on quantification of every 4^th^ section. Animals were excluded from the analysis if they showed lack of a cell deposit in the SRS or had evidence of a fulminant immune reaction, because macrophage infiltration is associated with reduced GFP exchange (West *et al*, 2010). We observed, by way of Pearson coefficient and linear regression analyses, a strong correlation between the number of GFP^+^ cells in the recipient ONL (corresponding to recipient cells that received transferred GFP) and the number of GFP^+^ cells in the SRS. Thus, we normalized the number of GFP^+^ cells in the ONL to the number of GFP^+^ cells in the SRS and refer to this parameter as material transfer index. In the *Crx::Cre* transplantation into *ROSA^mT/mG^* mice, mTdTomato switches to mGFP when Cre recombinase is transferred from the donor to the recipient. In these experiments, the number of mGFP^+^ cells in the ONL was quantified by counting all GFP^+^ cells where the entire cell was located in the recipient ONL.

##### Quantification using flow cytometry

Retinal cells were separated from debris through FSC‐A vs SSC‐A, and then singlets were discriminated by width vs height for both forward and side scatter. Live eFluor780^−^ cells were selected and further gated for photoreceptors by CD73^+^. Biaxial plots were used to identify the GFP^+^ vs tdTomato^+^ populations or MTG^+^ vs MTR^+^ populations. Fluorescence minus one ‐controls were used to identify gates for each fluorophore. Single fluorescence controls of the same cell type analysed were used to calculate compensation for each individual experiment. At least 1,000 live cells and 600 CD73^+^ cells were analysed for each technical replicate. Data analysis indicates the percentage of double‐positive cells (Q2) from total fluorescent eFluor^−^: CD73^+^ population (Q1+Q2+Q3). Each plotted value represents a triplicate mean for one biological replicate. At least three biological replicates were analysed for each condition. For pharmacological manipulations, the frequency of MT in treated co‐cultures was normalized to untreated control conditions from the same experimental day.

##### Quantification of donor and acceptor photoreceptor connections in vivo

Donor–acceptor photoreceptor connectivity was evaluated from confocal, two‐photon and light sheet microscopy images using Imaris 9.0.2 software. Whole transplanted eyes or whole‐mounted retinas from transplanted eyes were imaged and GFP^+^ acceptor photoreceptors in the recipient ONL were identified. These GFP^+^ photoreceptors were then classified into one of three groups based on the presence of a GFP^+^ protrusion connecting donor and acceptor photoreceptors: (i) contiguous, defined as donor and acceptor connections with a gap of less than 3 µm, (ii) proximal, defined as donor and acceptor connected with a gap of between 3 and 5 µm, and (iii) not contiguous, defined as cells connected by a gap of greater than 5 µm. For graphical representation of the data, we compiled two categories, contiguous and proximal, as positive for contact.

##### Quantification of protrusions in live photoreceptor cultures

A systematic sampling of three images per dish was performed in 8 *Nrl:GFP* culture preparations after 3 DIV. A total of 24 3D‐reconstructed images were analysed on Imaris 9.0.2 (Bitplane Inc., Zurich, Switzerland). Twelve of them were stained with SiR‐Actin (100 μM) (CY‐SC001, Cytoskeleton Inc. Denver, CO, USA) and 12 with ViaFluor^®^ (50 μM) (70062, Biotium, Fremont, CA, USA). We identified a total of 590 and 278 GFP^+^ protrusions extending from photoreceptors in actin and tubulin‐stained cultures, respectively. Each protrusion was visually assessed for cytoskeletal marker positivity and labelled accordingly using the Spots module of the software. Then, by visual inspection, protrusions were manually classified as (i) connected, e.g., connecting two cells, or unconnected; (ii) suspended in the media or attached, e.g., in contact with the bottom of the dish at any point along their length; and (iii) distal tip morphology, e.g., straight or terminating with distal swelling (spherule‐like). All data were collected and organized in Microsoft Excel (Office Excel, version 16.35, 2020; Microsoft Corp., Redmond, WA, USA) and analysed in GraphPad Prism version 7.0a sheets (GraphPad Software Inc. La Jolla, CA, USA).

##### Quantitation of protrusion outgrowth from transplanted donor photoreceptors *in vivo*

To quantify neurite length in transplanted donor photoreceptors, we used the Filaments module in Imaris 9.0.2 (Bitplane Inc., Zurich, Switzerland) to trace GFP^+^ protrusions. Protrusions measuring less than 5 µm in length were excluded from the analysis. Average protrusion length was defined as the sum of the length of all the protrusions normalized to the total number of protrusions. Total protrusion length per cell was defined as the sum length of all the protrusions normalized to the number of cells with a protrusion.

##### Immunoblot densitometry

Densitometric analysis of the protein was performed using ImageJ (National Institutes of Health, Bethesda, Maryland, USA). Briefly, the protein of interest was quantified by normalizing to GAPDH controls and the normalized protein amounts were compared between treatment groups.

##### Statistics and reproducibility

Experiments in this study were performed independently at least two times (*in vivo*) and at least three times (*in vitro*). No inconsistent results were excluded. Flow cytometric analyses of cultured retinal dissociates were performed on at least three independent biological replicates and with at least two technical replicates per condition, and samples were run on the same cytometers to reduce inter‐experimental variability. All the data were collected and organized in Microsoft Excel spreadsheets (Office Excel, version 16.35, 2020; Microsoft Corp., Redmond, WA, USA). All the graphical representation and statistical analysis were performed in GraphPad Prism version 7.0a sheets (GraphPad Software Inc. La Jolla, CA, USA). Data are represented as mean ± SEM. Statistical analyses were performed using a one‐way ANOVA with Tukey's multiple comparison test, extra‐sum‐of‐squares F‐test or unpaired *t*‐test where appropriate. The *P*‐values are represented as n.s. not statistically significant, ****P ≤ 0.0001, ***P < 0.001, **P < 0.01 and *P < 0.05.

## Author contributions

AOM, NEY, ELST, AES, PEN and VAW conceptualized the study. AOM, NEY, ELST, LC, AG, NT, ZCL, SL, PEN, CS, RB and VAW developed methodology. AOM, NEY, ELST, LC, AG, NT, ZCL, PD, NTP, PEN and VAW performed investigations. AOM, NEY, ELST, AG and VAW carried out formal analysis. AOM and VAW wrote the original draft of the manuscript, and PEN, CS, RB and VAW revised and edited. CS, RB and VAW acquired funding and provided resources, and VAW supervised.

## Conflict of interest

The authors declare that they have no conflict of interest.

## Supporting information



Appendix

Expanded View Figures PDF

Source Data for Expanded View and Appendix

Movie EV1

Movie EV2

Movie EV3

Movie EV4

Movie EV5

Movie EV6

Movie EV7

Source Data for Figure 1

Source Data for Figure 2

Source Data for Figure 4

Source Data for Figure 5

Source Data for Figure 7

Source Data for Figure 8

## Data Availability

All data analyses for this study are provided in this manuscript and supplementary information files. This study includes no data deposited in external repositories.
