# Supplementary material for: Photoreceptor nanotubes mediate the in vivo exchange of intracellular material
Source: EMBO J. 2021 Sep 8;40(22):e107264. doi: 10.15252/embj.2020107264 (PMC8591540; doi:10.15252/embj.2020107264)
Supplement: Supplementary file 1 — Appendix [file EMBJ-40-e107264-s009.pdf]

Appendix

Table of contents

Supplementary Figures	Page 2 to page 7
Appendix Figure S1	Page 2 to page 3
Appendix Figure S2	Page 4 to page 5
Appendix Figure S3	Page 6 to page 7
Appendix Table S1	Page 8
Appendix Table S2	Page 8
Appendix Table S3	Page 9
Appendix Table S4	Page 9
Appendix Table S5	Page 10
Appendix Table S6	Page 11 to page 12
Appendix Table S7	Page 12 to page 13
Appendix References	Page 13

Supplementary Figures

Appendix Figure S1

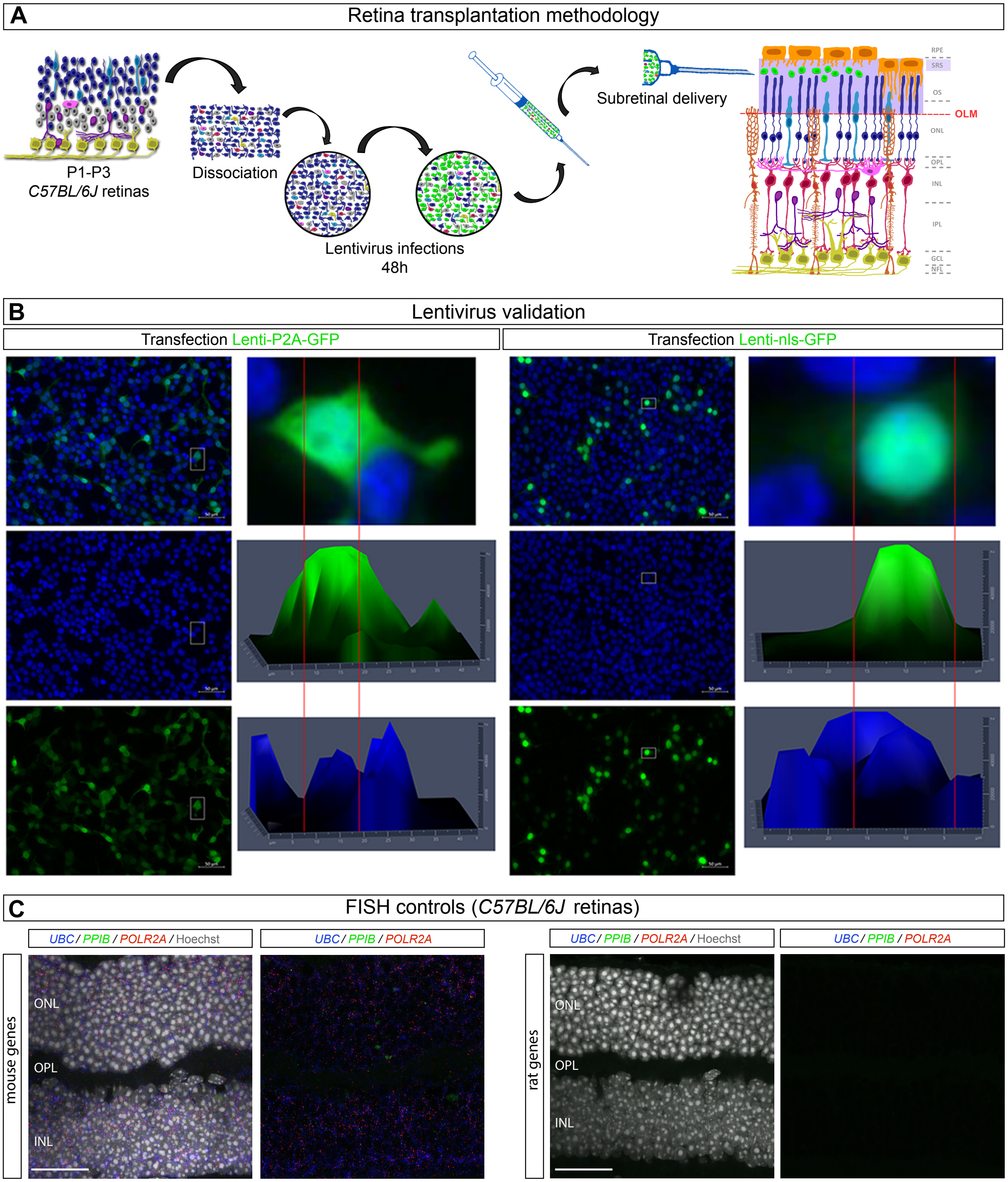

Appendix Figure S1. Validation and experimental controls from Figure 2. A, Schematic showing the general methodology of retinal transplantation after lentiviral infection of photoreceptors. **B**, Validation of the localization of GFP expression in HEK293T cells transfected with CAG-P2A-GFP or CAG-nls-GFP. Histograms of the GFP versus Hoechst fluorescence patterns demonstrate cytoplasmic localization of the Lenti-P2A-GFP transfected cells and nuclear localization of Lenti-nls-GFP transfected cells. **C**, Control of FISH. Scale bars: 50 μm.

Appendix Figure S2

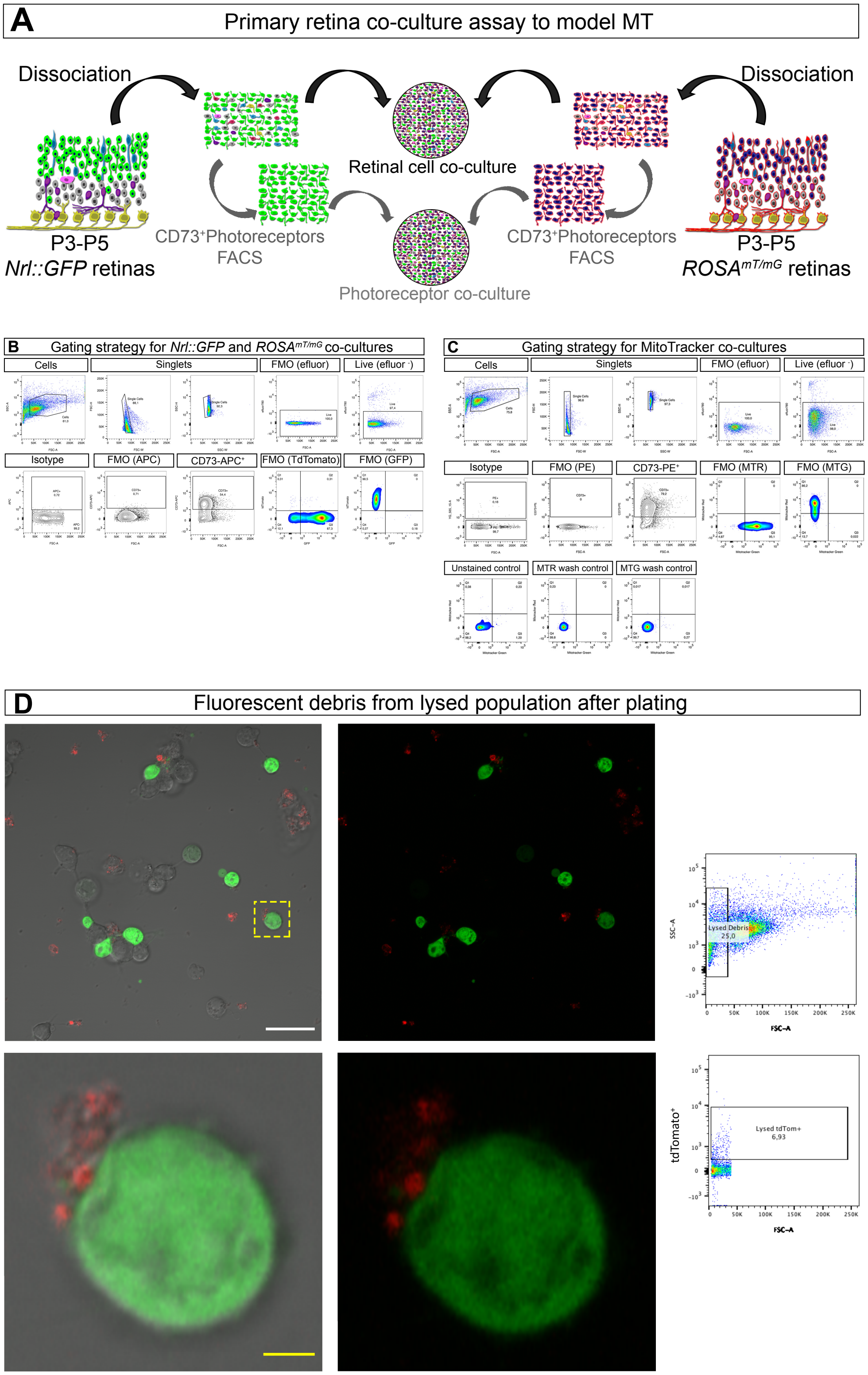

Appendix Figure S2. Schematic representation of the *in vitro* methodology and flow-cytometry gating strategy for the analysis of MT in photoreceptor co-cultures. A, Co-culture assay schematic. **B**, The fluorescence combinations used for this experiment were CD73-APC, eFluor780 viability dye, endogenous GFP from *Nrl::GFP* mice, and endogenous tdTomato from *ROSA*$^{mT/mG}$ mice. Compensation was performed using mouse retinal photoreceptors that were unstained, stained with CD73-APC, stained with eFluor780, and expressing endogenous GFP or tdTomato. Fluorescence minus one -controls were used to define all thresholds. Isotype-APC controls were used to differentiate non-specific background signal from CD73-APC positive signals. The gating tree was set as follows: FSC/SSC showing the distribution of cells based on size and granularity to; Singlets by FSW-W/FSC-H to SSC-W/SSC-H to; Live cell discrimination by eFluor780 negative to; FSC-A/CD73-APC positive for photoreceptor subset to; GFP/tdTomato with biexponential scaling and quadrant gating to analyse proportion of double-labelled cells in co-cultures. A total of 19,658 cells were analysed with 5,175 cells examined in the final gate (Fig. 2). Statistics presented represent the percentage of cells within the specific gate. **C**, Gating strategy for MitoTracker co-cultures. FSC/SSC showing the distribution of cells based on size and granularity to; singlets by FSW-W/FSC-H to SSC-W/SSC-H to; Live cell discrimination by eFluor780 negative to; FSC-A/CD73-PE positive for photoreceptor subset to; MTG/MTR with biexponential scaling and quadrant gating to analyse proportion of double-labelled photoreceptors in co-cultures. **D**, Validation of the presence of fluorescence debris from the lysed population after plating. *Left:*, Live confocal images of P3-5 *Nrl::GFP* retinal dissociates and lysed *ROSA*$^{mT/mG}$ imaged 3 hrs after plating. *Right:*, Flow-cytometry analysis of fluorescence debris 3 days after plating. White scale bar: 20 µm. Yellow scale bar: 2 µm.

Appendix Figure S3

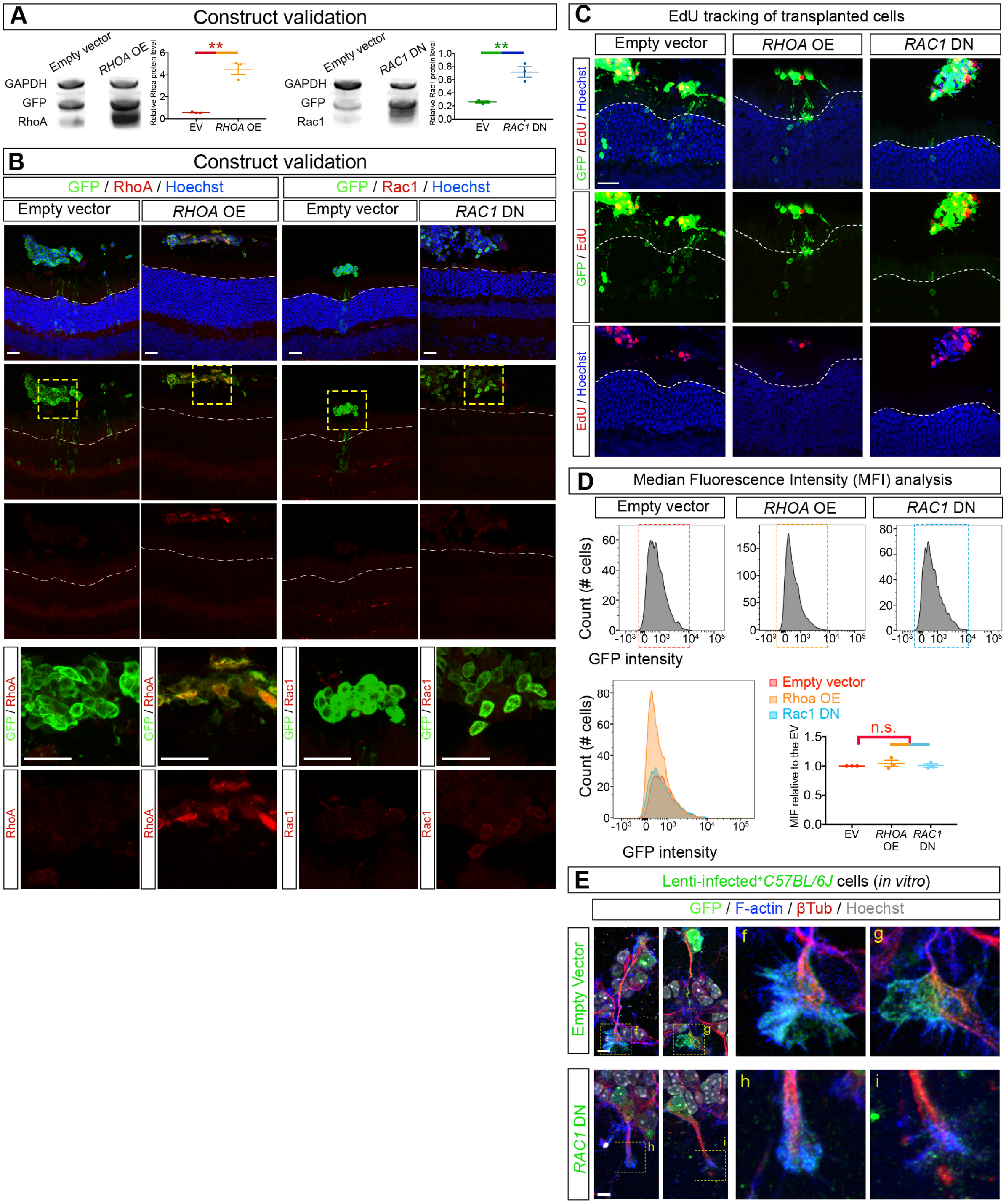

Appendix Figure S3. Validation of constructs and confirmation of GFP transfer after transplantation of *RHOA* OE and dominant negative *RAC1* infected cells. A, *Left:* Immunoblotting of HEK293T transfected cells confirms the presence of GFP and the overexpression of *RHOA*. GAPDH-36KD, GFP-33KD, RhoA-21KD (n=3 per group). *Right:* Immunoblotting of HEK293T transfected cells confirms the presence of GFP and the presence of dominant negative *RAC1*. GAPDH-36KD, GFP-33KD, Rac1-21KD. **B**, Validation of the constructs 21 days after transplantation by immunofluorescence for RHOA and RAC1 in cryostat sections of transplanted eyes with empty vector control or RHOA OE and dominant negative RAC1 infected photoreceptors. **C**, Presence of GFP^+/EdU^+ donor photoreceptors exclusively in the SRS, and only GFP^+/EdU^- photoreceptors in the outer nuclear layer of the recipient retina 21 days after transplantation confirms the lack of donor cell integration into the recipient retina. **D**, Median fluorescence intensity analysis of the GFP in photoreceptors after infection of the empty vector, *RHOA* OE and *RAC1* DN (n=3 per group). **E**, Analysis of growth cone morphology shows a collapse of the growth cone in donor photoreceptors infected with *RAC1* DN compared to the empty vector control. White dashed lines delimit the apical side of the ONL of the recipient retina. All data presented as mean ± SEM; n.s. not statistically significant, **p < 0.01; T-test or one-way ANOVA with Tukey's *post-hoc* multiple comparisons test. Scale bars: 50 µm.

Appendix Table S1. Mouse strains used in this study

Strain	Background	Reference
C57BL/6J	N/A	The Jackson Laboratory Stock #000664
Nrl::GFP	*C57BL/6J*	*(Akimoto et al., 2006)*
$Nrl^{-/-}$	*C57BL/6J*	*(Mears, Kondo et al., 2001)*
$ROSA^{mT/mG}$	*Mixed*	*$Gt(ROSA)26Sor^{tm4(ACTB\text{-}tdTomato,\text{-}EGFP)Luo}$*/J (Jackson Labs Stock #007576)
$Nrl^{-/-}$; $ROSA^{mT/mG}$	*Mixed*	Generated by crossing *$Nrl^{-/-}$* and *$ROSA^{mT/mG}$* (above)
Crx::Cre	*C57BL/6J*	*(Prasov & Glaser, 2012)*
Nrl::GFP; $Prphr2^{-/-}$	*C57BL/6J*	Generated by crossing *Nrl::GFP* and C3A.Cg-*$Pde6b^{+}$ $Prph2^{Rd2}$*/J (Jackson Lab; Stock #001979).

Appendix Table S2. PCR genotyping primers

Target	Primer (5' - 3')
**Nrl wildtype, forward**	GTGTTCCTTGGCTGGAAAGA
**Nrl wildtype, reverse**	CTGTTCACTGTGGGCTTTCA
**Nrl knockout, forward**	TGAATACAGGGACGACACCA
**Nrl knockout, reverse**	GTTCTAATTCCATCAGAAGCTGAC
**mT/mG, common**	CTCTGCTGCCTCCTGGCTTCT
**mT/mG, wildtype reverse**	CGAGGCGGATCACAAGCAATA
**mT/mG, mutant, reverse**	TCAATGGGCGGGGGTCGTT
**Prph2 common, forward**	GGCCCTGTATCCAGTACCAG
**Prph2 wildtype, reverse**	GCATGGGCAACATAATGAGA
**Prph2 mutant, reverse**	GTCTGAAGCTCAACCAGAACA
**Crx BAC, forward**	CTCTGTTCCTGCTTATTGGGG
**Crx BAC, reverse**	GAGTCTGGGACATGTTCAGTT

**Cre, forward**	GCATTTCTGGGGATTGCTTA
**Cre, reverse**	AGAGAAGTGGGGTGGCTTTT

Appendix Table S3. Retinal explant media

Reagent	Cat #, Supplier	Final Concentration
**DMEM, high glucose, GlutaMAX, pyruvate**	10569, Thermo Fisher Scientific, Mississauga, ON, Canada	0.5 ml/ml
**Nutrient Mixture F12 Ham**	N6658, Merck Millipore Sigma Aldrich, Oakville, ON, Canada	0.5 ml/ml
**Sato's Supplement***	See Table M4	10 ul/ml
**Insulin**	I6634, Merck Millipore Sigma Aldrich, Oakville, ON, Canada	10 µg/ml
**N-acetyl-L-cysteine**	A9165, Merck Millipore Sigma Aldrich, Oakville, ON, Canada	60 µg/ml
**Gentamicin**	15710, Thermo Fisher Scientific, Mississauga, ON, Canada	10 µg/ml

Appendix Table S4. Sato's Supplement

Reagent	Cat #, Supplier	Final Concentration
**CO_2 Independent Media**	18045088, Thermo Fisher Scientific, Mississauga, ON, Canada	Solvent
**Apotransferrin**	T1147, Merck Millipore Sigma Aldrich, Oakville, ON, Canada	10 mg/ml
**Bovine Serum Albumin**	A4161, Merck Millipore Sigma Aldrich, Oakville, ON, Canada	10 mg/ml
**Progesterone, 1 mg/mL stock, dissolved in 100% Ethanol**	P8783, Merck Millipore Sigma Aldrich, Oakville, ON, Canada	6 ug/ml
**Putrescine**	P5780, Merck Millipore Sigma Aldrich, Oakville, ON, Canada	1.6 mg/ml
**Sodium Selenite, 4 mg/mL stock, dissolved in 0.1 M NaOH**	S5261, Merck Millipore Sigma Aldrich, Oakville, ON, Canada	4 ug/ml

Appendix Table S5. List of plasmid and expression vectors

Plasmid	Common name	Transgene	Description
**pLenti-CAG-P2A-EGFP**	P2A-GFP	Empty vector	Derived from pLenti-CAG-IRES-GFP. IRES-GFP was replaced with P2A-EGFP.
**pLenti-CAG-P2A-EGFP-nls**	P2A-GFP-nls	Empty vector with nls tagged GFP reporter	Derived from pLenti-CAG-P2A-EGFP. Nuclear localization signal (nls) was added at the 3' end of GFP sequence.
**pLV-Mito-DsRed**	Mito-DsRed	Mitochondria F1F0-ATP synthase	Addgene plasmid # 44386. A gift from Dr. Pantelis Tsoulfas
**pLenti-CAG-RHOA-P2A-EGFP**	RhoA OE	Human *RHOA* (NM_001664)	Derived from pLenti-CAG-P2A-EGFP. Human RhoA was subcloned into pLenti-CAG-P2A-EGFP in frame with P2A-EGFP.
**pLenti-CAG-RAC1(T17N)-P2A-EGFP**	RAC1 DN	Human *RAC1-DN* (NM_006908)	Derived from pLenti-CAG-P2A-EGFP. Human *RAC1(T17N)* was subcloned into pLenti-CAG-P2A-EGFP in frame with P2A-EGFP. AC (residues 50-51) of *RAC1* were replaced to CT.
**pMD2.G**		Enveloping vector	Addgene #12259. A gift from Dr. Didier Trono.
**psPAX2**		Packaging vector	Addgene #12260. A gift from Dr. Didier Trono.

Appendix Table S6. Primary and secondary antibodies used for immunohistochemistry

Antibody	Species	Dilution	Cat #, Supplier
**Primary Antibodies**			
Anti-EGFP	Goat	1:500	600-101-215, Rockland Immunochemicals, Inc. Pottstown, PA, USA.
Anti-RFP	Rabbit	1:500	600-401-379, Rockland Immunochemicals, Inc. Pottstown, PA, USA.
Anti-GNAT1	Rabbit	1:200	Sc-389, Santa Cruz Biotechnology, Inc. Mississauga, ON, Canada.
Anti-ATP5B	Mouse	1:500	Ab5432, Abcam plc. Cambridge, UK.
Anti-Prphr2	Mouse	1:10 (supernatant)	Gif from Dr. Robert S Molday *ref: Hum Mol Genet. 27:295-306 (2018).*
Anti-αTubulin	Mouse	1:200	Sc-53646, Santa Cruz Biotechnology, Inc. Mississauga, ON, Canada.
Anti-βTubulin	Mouse	1:100	MA5-11740, Thermo Fisher Scientific, Mississauga, ON, Canada.
Anti-SV2A	Mouse	1:100	SV2, Developmental Studies Hybridoma Bank, University of Iowa, Iowa City, IA, USA.
Anti-Bassoon	Mouse	1:5000	ADI-VAM-PS003-D, Enzo Life Sciences, Inc., Farmingdale, NY, USA
Anti-α βTubulin	Rabbit	1:100	2148, Cell Signaling Technologies, Danvers, MA, USA

Anti-RhoA	Rabbit	1:500	2117, Cell Signaling Technologies, Danvers, MA, USA.
Anti-Rac1	Mouse	1:500	05-389, Merck Millipore Sigma Aldrich, Oakville, ON, Canada.
**Secondary Antibodies**			
Anti-goat 488	Donkey	1:500	A-11055, Thermo Fisher Scientific, Mississauga, ON, Canada.
Anti-mouse 555	Donkey	1:500	A-31570, Thermo Fisher Scientific, Mississauga, ON, Canada.
Anti-mouse 647	Donkey	1:500	A-31571, Thermo Fisher Scientific, Mississauga, ON, Canada.
Anti-rabbit 555	Donkey	1:500	A-31572, Thermo Fisher Scientific, Mississauga, ON, Canada.
Anti-rabbit 647	Donkey	1:500	A-31573, Thermo Fisher Scientific, Mississauga, ON, Canada.

Appendix Table S7. Antibodies used for western blotting

**Antibody**	**Species**	**Dilution**	**Cat #, Supplier**
**Primary Antibodies**			
Anti-βTubulin	Rabbit	1:1000	Ab6046, Abcam plc. Cambridge, UK.
Anti-EGFP	Goat	1:500 / 1:1000	600-101-215, Rockland Immunochemicals, Inc. Pottstown, PA, USA.
Anti-Flotilin1	Mouse	1:800	610821, BD Transduction Laboratories™, San Jose, CA, USA.
Anti-GAPDH	Mouse	1:10000	CB1001, Calbiochem, Merck Millipore Sigma Aldrich, Oakville, ON, Canada

Anti-RhoA	Rabbit	1:1000	2117, Cell Signaling Technologies, Danvers, MA, USA.
Anti-Rac1	Mouse	1:1000	05-389, Merck Millipore Sigma Aldrich, Oakville, ON, Canada.
**Secondary Antibodies**			
Anti-goat IRDye 800CW	Donkey	1:10000	926-32214, LI-COR Biosciences, Lincoln, NE, USA.
Anti-goat IRDye 680RD	Donkey	1:10000	926-68074, LI-COR Biosciences, Lincoln, NE, USA.
Anti-mouse IRDye 800CW	Donkey	1:10000	926-32212, LI-COR Biosciences, Lincoln, NE, USA.
Anti-mouse IRDye 680RD	Donkey	1:10000	926-68072, LI-COR Biosciences, Lincoln, NE, USA.
Anti-rabbit IRDye 800CW	Donkey	1:10000	926-32213, LI-COR Biosciences, Lincoln, NE, USA.
Anti-rabbit IRDye 680RD	Donkey	1:10000	926-68073, LI-COR Biosciences, Lincoln, NE, USA.

Appendix References

Akimoto M, Cheng H, Zhu D, Brzezinski JA, Khanna R, Filippova E, Oh EC, Jing Y, Linares JL, Brooks M, Zareparsi S, Mears AJ, Hero A, Glaser T, Swaroop A (2006) Targeting of GFP to newborn rods by Nrl promoter and temporal expression profiling of flow-sorted photoreceptors. *Proceedings of the National Academy of Sciences of the United States of America* 103: 3890-5

Mears AJ, Kondo M, Swain PK, Takada Y, Bush RA, Saunders TL, Sieving PA, Swaroop A (2001) Nrl is required for rod photoreceptor development. *Nat Genet* 29: 447-52

Prasov L, Glaser T (2012) Pushing the envelope of retinal ganglion cell genesis: context dependent function of Math5 (Atoh7). *Developmental biology* 368: 214-30
