# Supplementary material for: Photoreceptor nanotubes mediate the in vivo exchange of intracellular material
Source: EMBO J. 2021 Sep 8;40(22):e107264. doi: 10.15252/embj.2020107264 (PMC8591540; doi:10.15252/embj.2020107264)
Supplement: Supplementary file 2 — Expanded View Figures PDF [file EMBJ-40-e107264-s015.pdf]

Expanded View Figures

Figure EV1. Positive linear correlation between the numbers of GFP$^+$ donor and acceptor photoreceptors after transplantation. Inefficient MT in recipient photoreceptors after transplantation of cancer cell lines or primary cortical neurons.

A Significant, positive linear correlation between the number of GFP$^+$-donor photoreceptors in the SRS and the number GFP$^+$-acceptor photoreceptors in the recipient retina of *C57BL/6J* (14 days (n = 9), 21 days (n = 6), 90 days (n = 5)) or *Nrl*$^{-/-}$ (14 days (n = 7), 21 days (n = 4), 90 days (n = 4)). r: linear correlation coefficient; R^2: coefficient of determination; and slope: slope of the regression line.

B, C Transplanted GFP$^+$-WERI-Rb-1 (retinoblastoma) (B) and GFP$^+$-BT088 (glioma) (C) cell lines result in inefficient MT.

D MT between transplanted primary GFP$^+$-cortical neurons and the recipient retina is also inefficient.

Data information: n.s. not statistically significant, ***$P < 0.001$, **$P < 0.01$ and *$P < 0.05$; Pearson correlation coefficient and linear regression analysis. White dashed lines delimit the apical side of the ONL of the recipient retina; grey dashed lines delimit the apical border of the inner segments in the recipient retina. Scale bars: 50 μm.
Source data are available online for this figure.

© 2021 The Authors

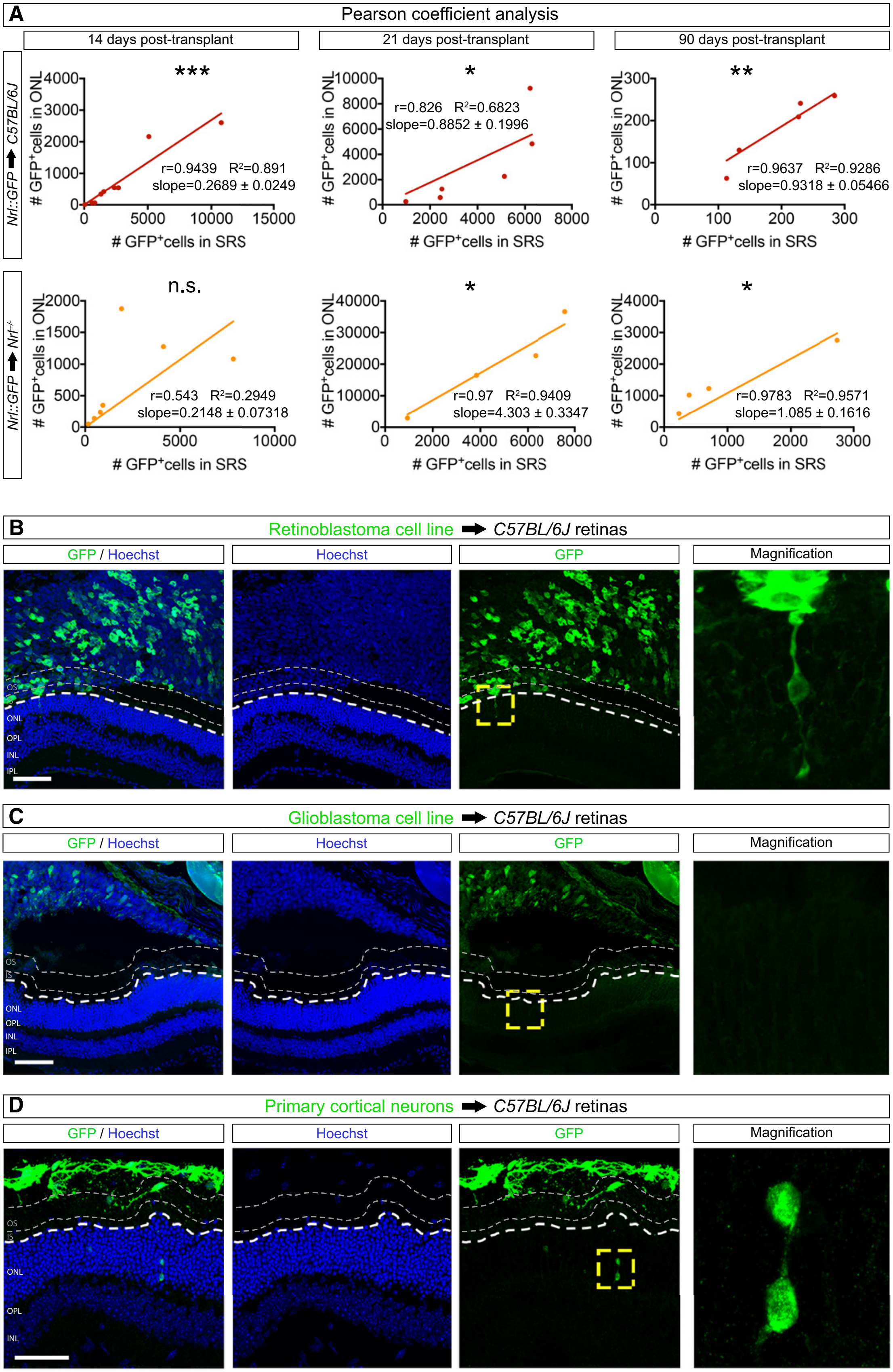
A
Pearson coefficient analysis
14 days post-transplant
21 days post-transplant
90 days post-transplant
Nrl::GFP ➡ C57BL/6J
Nrl::GFP ➡ Nrl-/-
***
r=0.9439 R²=0.891
slope=0.2689 ± 0.0249
*
r=0.826 R²=0.6823
slope=0.8852 ± 0.1996
**
r=0.9637 R²=0.9286
slope=0.9318 ± 0.05466
n.s.
r=0.543 R²=0.2949
slope=0.2148 ± 0.07318
*
r=0.97 R²=0.9409
slope=4.303 ± 0.3347
*
r=0.9783 R²=0.9571
slope=1.085 ± 0.1616
# GFP+cells in ONL
# GFP+cells in SRS
B
Retinoblastoma cell line ➡ C57BL/6J retinas
GFP / Hoechst
Hoechst
GFP
Magnification
OS
IS
ONL
OPL
INL
IPL
C
Glioblastoma cell line ➡ C57BL/6J retinas
GFP / Hoechst
Hoechst
GFP
Magnification
D
Primary cortical neurons ➡ C57BL/6J retinas
GFP / Hoechst
Hoechst
GFP
Magnification

Figure EV1.

Figure EV2. Photoreceptor-derived extracellular vesicles do not mediate GFP transfer.

A Electron microscopy images of EVs harvested from photoreceptor cultures. Arrowheads point to the bilipid membrane.

B Western blot analysis of EVs from *Nrl::GFP* and $Nrl^{-/-}$; $Ccdc136^{GFP/GFP}$ photoreceptors confirm the presence of GFP in vesicles.

C *Nrl::GFP*; $Prphr2^{-/-}$ transplanted photoreceptors ($n = 8$) show no differences in MT index compared to *Nrl::GFP* transplanted photoreceptors ($n = 10$).

D Pharmacological inhibition of different vesicle-release pathways: GW4869 (exosome secretion blocker); chloroquine (autophagy induction blocker); and amitriptyline (ectosome secretion blocker) show no effect on MT *in vivo* ($n = 3$ per group).

E Pharmacological inhibition of different vesicle-release pathways: GW4869 (exosome secretion blocker); chloroquine (autophagy induction blocker); and amitriptyline (ectosome secretion blocker) show no effect on MT *in vitro* ($n = 8$, $n = 3$ and $n = 3$, respectively).

F Quantification for C–E.

Data information: All data are presented as mean ± SEM; n.s. not statistically significant, one-way ANOVA with Tukey's *post hoc* multiple comparison test. White dashed lines delimit the apical side of the ONL of the recipient retina. Scale bars: A: 100 nm. C, D: 50 μm.
Source data are available online for this figure.

 © 2021 The Authors

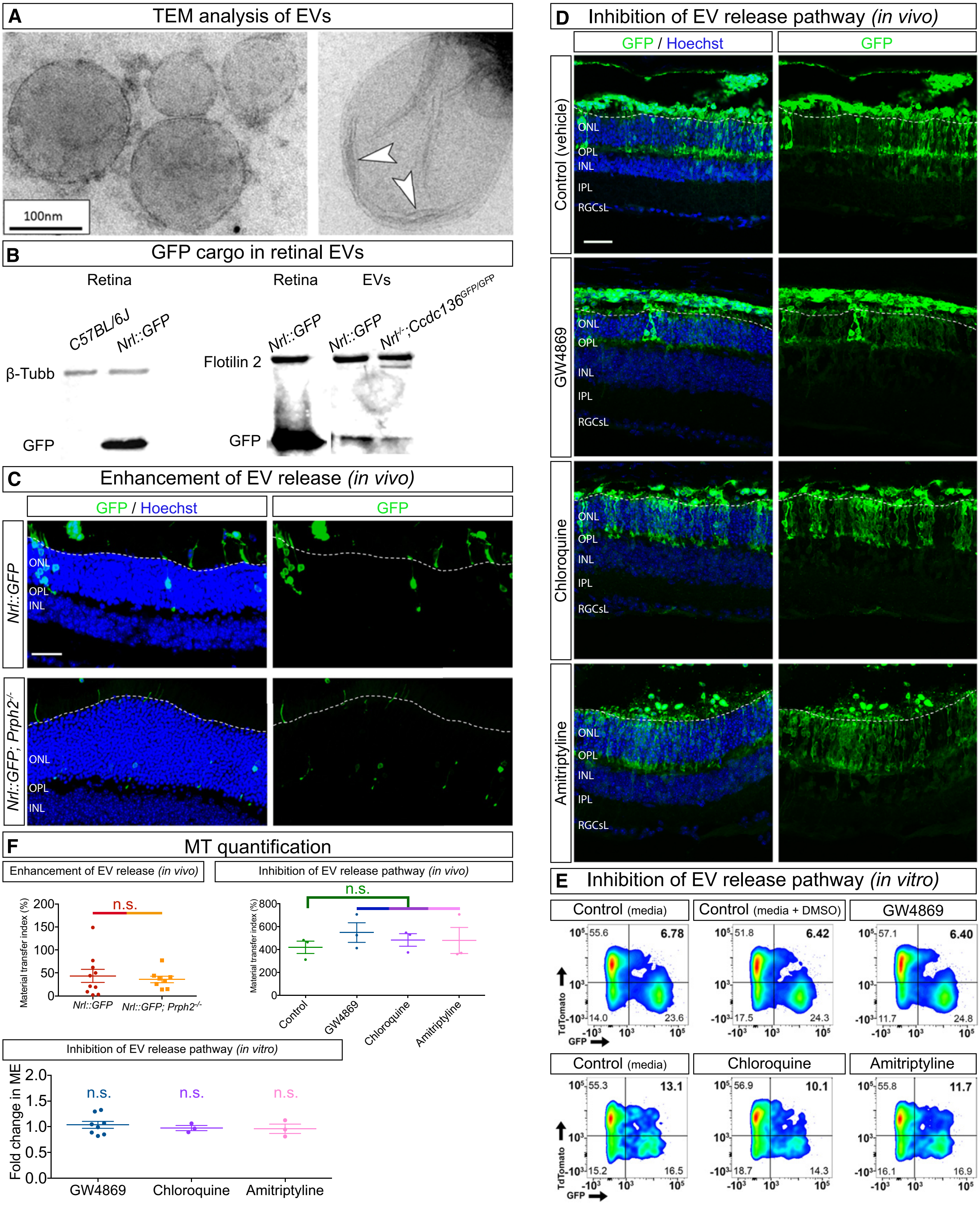
A TEM analysis of EVs
100nm
B GFP cargo in retinal EVs
Retina
Retina
EVs
C57BL/6J
Nrl::GFP
Nrl::GFP
Nrl::GFP
Nrl-/-;Ccdc136GFP/GFP
β-Tubb
GFP
Flotilin 2
GFP
C Enhancement of EV release (in vivo)
GFP / Hoechst
GFP
Nrl::GFP
Nrl::GFP; Prph2-/-
ONL
OPL
INL
D Inhibition of EV release pathway (in vivo)
GFP / Hoechst
GFP
Control (vehicle)
GW4869
Chloroquine
Amitriptyline
ONL
OPL
INL
IPL
RGCsL
E Inhibition of EV release pathway (in vitro)
Control (media)
Control (media + DMSO)
GW4869
Chloroquine
Amitriptyline
TdTomato
GFP
F MT quantification
Enhancement of EV release (in vivo)
Inhibition of EV release pathway (in vivo)
Inhibition of EV release pathway (in vitro)
Material transfer index (%)
n.s.
Control
GW4869
Chloroquine
Amitriptyline
Fold change in ME

Figure EV2.

Figure EV3. Mitochondria and GFP transcripts are found in cell protrusions.

A, B High magnification confocal image shows Mito-DsRed^{+} mitochondria (A) and GFP mRNA (B) in a protrusion connected to an acceptor photoreceptor in wild-type retinas transplanted with *Nrl::GFP* donor photoreceptors. White dashed lines delimit the apical side of the ONL of the recipient retina. Yellow arrows point to Mito-DsRed^{+} puncta (A) or transcripts (B) in the donor cells. Pink arrows point to Mito-DsRed^{+} puncta (A) or transcripts (B) in the acceptor cells. Scale bars: 10 μm.

 © 2021 The Authors

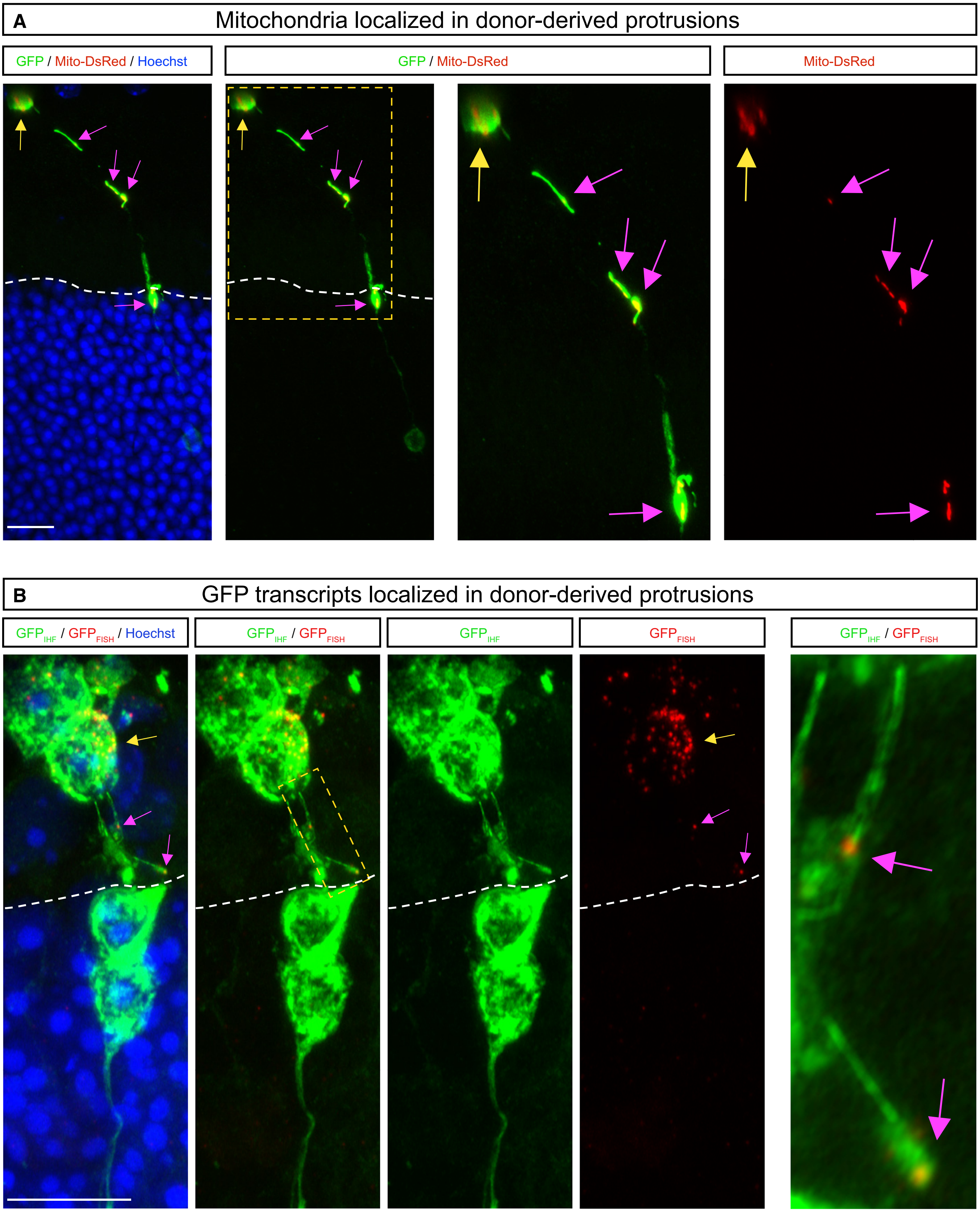
A
Mitochondria localized in donor-derived protrusions
GFP / Mito-DsRed / Hoechst
GFP / Mito-DsRed
Mito-DsRed
B
GFP transcripts localized in donor-derived protrusions
GFP_IHF / GFP_FISH / Hoechst
GFP_IHF / GFP_FISH
GFP_IHF
GFP_FISH
GFP_IHF / GFP_FISH

Figure EV3.

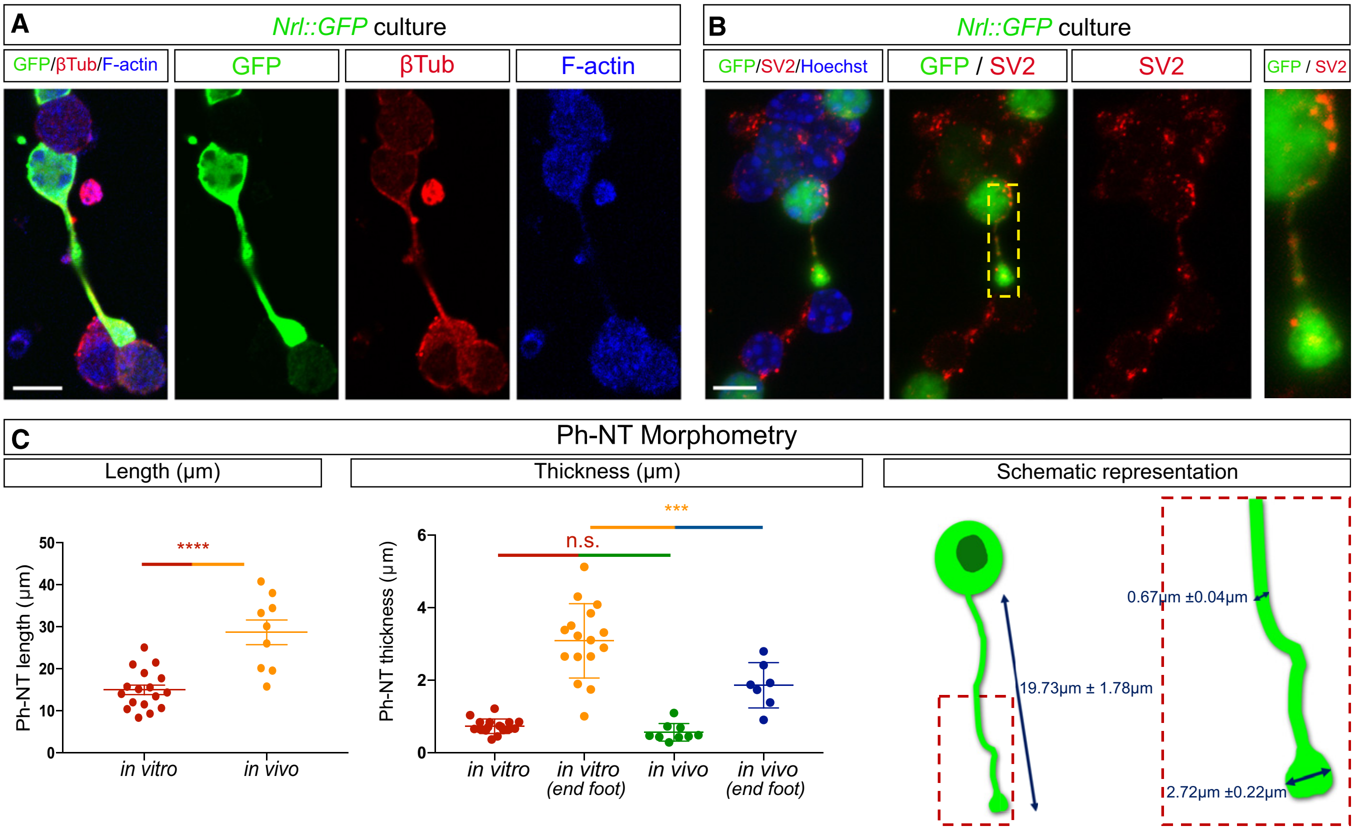

Figure EV4. Further characterization of cell protrusions.

A, B β-tubulin, F-actin (A), SV2 (B) and GFP staining in photoreceptor co-cultures of wild-type and *Nrl::GFP* retinal cells shows *Nrl::GFP* donor photoreceptors connected with acceptor photoreceptors via protrusions that co-localize with β-tubulin and F-actin and SV2.

C *In vitro* (protrusion length (n = 17), protrusion end-foot (n = 16) from a total sample (n = 9) (biological replicate (n = 3) technical replicates (n = 3) per each biological replicate) and *in vivo* (protrusion length n = 9) protrusion end-foot (n = 7) from wt cells transplanted in wt animals (n = 7)) measurements of length and thickness of protrusions.

Data information: All data are presented as mean ± SEM; n.s. not statistically significant, ****$P \leq 0.0001$ and ***$P < 0.001$. *t*-Test or one-way ANOVA with Tukey's *post hoc* multiple comparison test. Scale bars: D: 50 µm. A, B: 5 µm.
Source data are available online for this figure.

 © 2021 The Authors

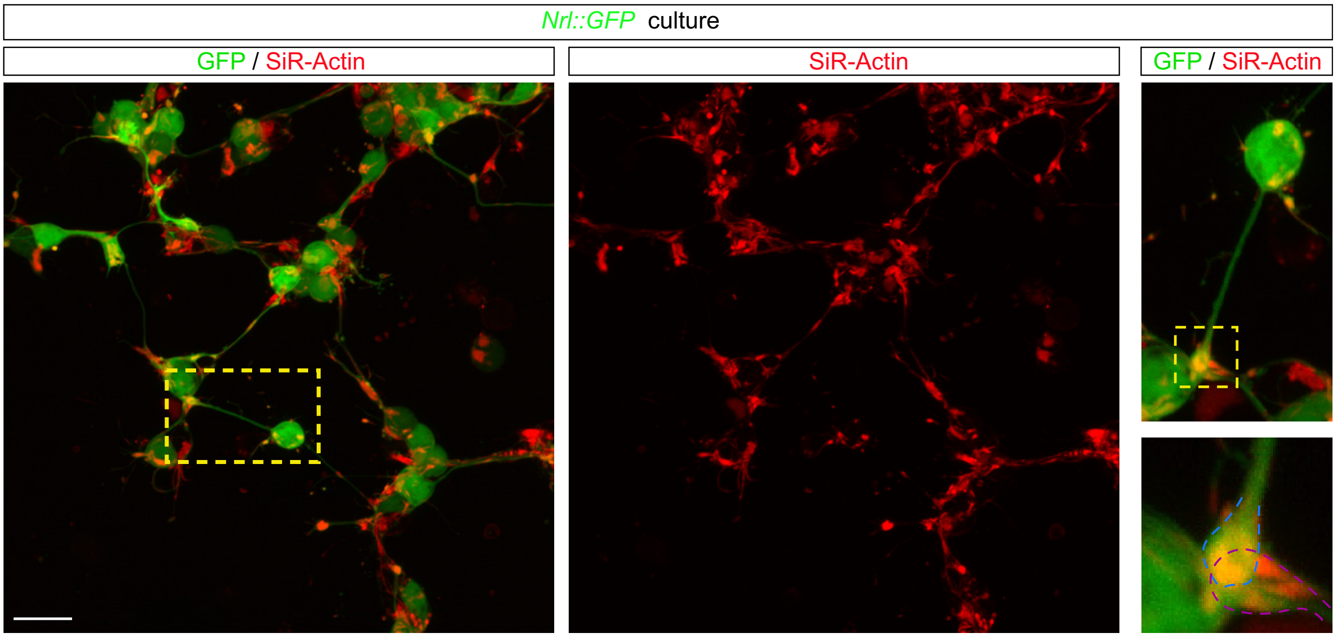

Figure EV5. *In vitro* characterization of protrusions.

Confocal image of live *Nrl::GFP* cultures detailing (right panel) that the terminus of a protrusion at the contact point between two photoreceptors contains actin (SiR-Actin^{+}). Blue and purple dashed lines delimit the end-foot of two different protrusions contacting the soma of another cell. Scale bar: 10 μm.
