# Supplementary material for: Photoreceptor nanotubes mediate the in vivo exchange of intracellular material
Source: EMBO J. 2021 Sep 8;40(22):e107264. doi: 10.15252/embj.2020107264 (PMC8591540; doi:10.15252/embj.2020107264)
Supplement: Supplementary file 4 — Movie EV1 [file EMBJ-40-e107264-s011.zip › Movie EV1/Movie EV1 legend.pdf]

**Movie EV1 (separate file). Corresponding to Figure 5B.** Confocal-acquired, 3D reconstruction of a *Nrl*$^{-/-}$ whole-mounted recipient retina shows that 21 days after transplantation with *Nrl::GFP* donor photoreceptors (top layer), the transplanted photoreceptors are still attached to the recipient retina. Furthermore, we observed the acceptor photoreceptors (GFP$^{+}$ host photoreceptors) connected to the donor photoreceptor through thin protrusions. Dynamic scale bar.
