# Supplementary material for: Photoreceptor nanotubes mediate the in vivo exchange of intracellular material
Source: EMBO J. 2021 Sep 8;40(22):e107264. doi: 10.15252/embj.2020107264 (PMC8591540; doi:10.15252/embj.2020107264)
Supplement: Supplementary file 5 — Movie EV2 [file EMBJ-40-e107264-s007.zip › Movie EV2/Movie EV2 legend.pdf]

Movie EV2 (separate file). Corresponding to Figure 5C. Confocal acquired, 3D reconstruction of a *C57BL6/J* whole-mounted recipient retina shows that 21 days after transplantation with *Nrl::GFP* donor photoreceptors (top layer), the transplanted photoreceptors are connected to acceptor photoreceptors via cell protrusions. GFP labelling is observed in a recipient rod and cone cell. Dynamic scale bar.
