# Supplementary material for: Photoreceptor nanotubes mediate the in vivo exchange of intracellular material
Source: EMBO J. 2021 Sep 8;40(22):e107264. doi: 10.15252/embj.2020107264 (PMC8591540; doi:10.15252/embj.2020107264)
Supplement: Supplementary file 6 — Movie EV3 [file EMBJ-40-e107264-s005.zip › Movie EV3/Movie EV3 legend.pdf]

Movie EV3 (separate file). Corresponding to Figure 5D. 3D reconstruction of a two-photon microscopy image of an intact cleared *C57BL6/J* eye shows an area with a large number of *Nrl::GFP* transplanted donor photoreceptors. GFP^{+} acceptor photoreceptors are also found in the same area. *Right:* A high magnification image showing transplanted and acceptor photoreceptors connected through a cell protrusion. Dynamic scale bar.
