# Supplementary material for: Photoreceptor nanotubes mediate the in vivo exchange of intracellular material
Source: EMBO J. 2021 Sep 8;40(22):e107264. doi: 10.15252/embj.2020107264 (PMC8591540; doi:10.15252/embj.2020107264)
Supplement: Supplementary file 7 — Movie EV4 [file EMBJ-40-e107264-s003.zip › Movie EV4/Movie EV4 legend.pdf]

Movie EV4 (separate file). Corresponding to Figure 5E. 3D reconstruction of a cleared intact *C57BL6/J* eye imaged by light-sheet microscopy shows an area with a large deposit of *Nrl::GFP* transplanted donor photoreceptors. GFP^{+} acceptor photoreceptors are also found in the same area. Dynamic scale bar.
