# Supplementary material for: Photoreceptor nanotubes mediate the in vivo exchange of intracellular material
Source: EMBO J. 2021 Sep 8;40(22):e107264. doi: 10.15252/embj.2020107264 (PMC8591540; doi:10.15252/embj.2020107264)
Supplement: Supplementary file 8 — Movie EV5 [file EMBJ-40-e107264-s006.zip › Movie EV5/Movie EV5 legend.pdf]

Movie EV5 (separate file). Characterization of photoreceptor protrusions that connect two cells. Image analysis of photoreceptor *in vitro* showing that photoreceptors are connected by cell protrusions that are suspended above the substratum. Scale bar: 3 µm
