# Supplementary material for: Photoreceptor nanotubes mediate the in vivo exchange of intracellular material
Source: EMBO J. 2021 Sep 8;40(22):e107264. doi: 10.15252/embj.2020107264 (PMC8591540; doi:10.15252/embj.2020107264)
Supplement: Supplementary file 9 — Movie EV6 [file EMBJ-40-e107264-s010.zip › Movie EV6/Movie EV6 legend.pdf]

**Movie EV6 (separate file). Characterization of the photoreceptor protrusion that do not connect cells.** Image analysis of photoreceptor *in vitro* showing that photoreceptors extend cell protrusions that have a distal growth cone and are attached to the substratum. Scale bar: 20 µm
