# Supplementary material for: Photoreceptor nanotubes mediate the in vivo exchange of intracellular material
Source: EMBO J. 2021 Sep 8;40(22):e107264. doi: 10.15252/embj.2020107264 (PMC8591540; doi:10.15252/embj.2020107264)
Supplement: Supplementary file 10 — Movie EV7 [file EMBJ-40-e107264-s002.zip › Movie EV7/Movie EV7 legend.pdf]

Movie EV7 (separate file). Mitochondria transfer. Live image analysis of cultured photoreceptors showing the bidirectional movement and intercellular transfer of $MitoTracker^{+}$ puncta between photoreceptors connected by a protrusion. Scale bar: 5 µm
