# Supplementary material for: Photoreceptor nanotubes mediate the in vivo exchange of intracellular material
Source: EMBO J. 2021 Sep 8;40(22):e107264. doi: 10.15252/embj.2020107264 (PMC8591540; doi:10.15252/embj.2020107264)
Supplement: Supplementary file 17 [file EMBJ-40-e107264-s008.pdf]

THE EMBO JOURNAL

Photoreceptor nanotubes mediate the in vivo exchange of intracellular material

Arturo Ortin Martinez, Nicole Yan, En Leh Samuel Tsai, Lacrimioara Comanita, Akshay Gurdita, Nobuhiko Tachibana, Zhongda Liu, Suying Lu, Parnian Dolati, Neno Pokrajac, Ahmed El-Sehemy, Philip Nickerson, Carol Schuurmans, Rod Bremner, and Valerie Wallace
DOI: 10.15252/embj.2020107264

Corresponding author: Valerie Wallace (valerie.wallace@uhnresearch.ca)

Review Timeline:

Submission Date:	13th Nov 20
Editorial Decision:	12th Jan 21
Revision Received:	9th Jun 21
Editorial Decision:	9th Jul 21
Revision Received:	15th Jul 21
Accepted:	19th Jul 21

Editor: Ieva Gailite / David del Alamo

Transaction Report:

(Note: With the exception of the correction of typographical or spelling errors that could be a source of ambiguity, letters and reports are not edited. Depending on transfer agreements, referee reports obtained elsewhere may or may not be included in this compilation. Referee reports are anonymous unless the Referee chooses to sign their reports.)

1st Editorial Decision 12th Jan 2021

Dear Dr. Wallace,

Thank you for submitting your manuscript for consideration by the EMBO Journal. I apologise for the protracted review process due to delayed submission of reviewer reports over the holiday period. We have now received three referee reports on your manuscript, which are included below for your information

As you will see from the comments, all reviewers find the study interesting. However, they also indicate a number of important concerns that affect core conclusions of the study, which would have to be addressed before they can support publication of the manuscript, in particular regarding the proposed protein, mRNA and organelle transfer selectivity via the nanotubes (all reviewers), further verification of nanotube identification (referee #1), as well as strengthening of the proposed role of actin modulators and microtubule cytoskeleton (referee #1). Furthermore, reviewers #2 and #3 raise concerns regarding the proposed mode of nanotube-mediated material transfer to the photoreceptor outer segment (reviewers #2 and #3)

Based on the general interest expressed by the reviewers, I would like to invite you to address the comments of all referees in a revised version of your manuscript. Please note that a strong referee support will be required for the acceptance of the revised manuscript.

I should add that it is The EMBO Journal policy to allow only a single major round of revision and that it is therefore important to resolve the main concerns at this stage. I would be happy to discuss the revision in more detail via email or phone/videoconferencing.

We have extended our 'scooping protection policy' beyond the usual 3 month revision timeline to cover the period required for a full revision to address the essential experimental issues. This means that competing manuscripts published during revision period will not negatively impact on our assessment of the conceptual advance presented by your study. Please contact me if you see a paper with related content published elsewhere to discuss the appropriate course of action.

When preparing your letter of response to the referees' comments, please bear in mind that this will form part of the Review Process File, and will therefore be available online to the community. For more details on our Transparent Editorial Process, please visit our website: https://www.embopress.org/page/journal/14602075/authorguide#transparentprocess

Please feel free to contact me if you have any further questions regarding the revision. Thank you for the opportunity to consider your work for publication. I look forward to receiving your revised manuscript.

Yours sincerely,

Ieva Gailite

Ieva Gailite, PhD
Scientific Editor
The EMBO Journal
Meyerhofstrasse 1

D-69117 Heidelberg
Tel: +4962218891309
i.gailite@embojournal.org

When submitting your revised manuscript, please carefully review the instructions below and include the following items:

1) a .docx formatted version of the manuscript text (including legends for main figures, EV figures and tables). Please make sure that the changes are highlighted to be clearly visible.

2) individual production quality figure files as .eps, .tif, .jpg (one file per figure).

3) a .docx formatted letter INCLUDING the reviewers' reports and your detailed point-by-point response to their comments. As part of the EMBO Press transparent editorial process, the point-by-point response is part of the Review Process File (RPF), which will be published alongside your paper.

4) a complete author checklist, which you can download from our author guidelines (https://wol-prod-cdn.literatumonline.com/pb-assets/embo-site/Author Checklist%20-%20EMBO%20J-1561436015657.xlsx). Please insert information in the checklist that is also reflected in the manuscript. The completed author checklist will also be part of the RPF.

5) Please note that all corresponding authors are required to supply an ORCID ID for their name upon submission of a revised manuscript.

6) Before submitting your revision, primary datasets produced in this study need to be deposited in an appropriate public database (see https://www.embopress.org/page/journal/14602075/authorguide#datadeposition). Please remember to provide a reviewer password if the datasets are not yet public. Please note that the Data Availability Section is restricted to new primary data that are part of this study. If no data deposition in external databases is needed for this paper, please then state in this section: This study includes no data deposited in external repositories.
*** Note - All links should resolve to a page where the data can be accessed. ***

7) Our journal encourages inclusion of *data citations in the reference list* to directly cite datasets that were re-used and obtained from public databases. Data citations in the article text are distinct from normal bibliographical citations and should directly link to the database records from which the data can be accessed. In the main text, data citations are formatted as follows: "Data ref: Smith et al, 2001" or "Data ref: NCBI Sequence Read Archive PRJNA342805, 2017". In the Reference list, data citations must be labeled with "[DATASET]". A data reference must provide the database name, accession number/identifiers and a resolvable link to the landing page from which the data can be accessed at the end of the reference. Further instructions are available at .

8) We would also encourage you to include the source data for figure panels that show essential data. Numerical data can be provided as individual .xls or .csv files (including a tab describing the data). For 'blots' or microscopy, uncropped images should be submitted (using a zip archive or a single pdf per main figure if multiple images need to be supplied for one panel). Additional information on source data and instruction on how to label the files are available at .

9) We replaced Supplementary Information with Expanded View (EV) Figures and Tables that are collapsible/expandable online (see examples in https://www.embopress.org/doi/10.15252/embj.201695874). A maximum of 5 EV Figures can be typeset. EV Figures should be cited as 'Figure EV1, Figure EV2" etc. in the text and their respective legends should be included in the main text after the legends of regular figures.

- For the figures that you do NOT wish to display as Expanded View figures, they should be bundled together with their legends in a single PDF file called *Appendix*, which should start with a short Table of Content. Appendix figures should be referred to in the main text as: "Appendix Figure S1, Appendix Figure S2" etc. See detailed instructions regarding expanded view here: .

- Additional Tables/Datasets should be labelled and referred to as Table EV1, Dataset EV1, etc. Legends have to be provided in a separate tab in case of .xls files. Alternatively, the legend can be supplied as a separate text file (README) and zipped together with the Table/Dataset file.

10) When assembling figures, please refer to our figure preparation guideline in order to ensure proper formatting and readability in print as well as on screen:
http://bit.ly/EMBOPressFigurePreparationGuideline

Please remember: Digital image enhancement is acceptable practice, as long as it accurately represents the original data and conforms to community standards. If a figure has been subjected to significant electronic manipulation, this must be noted in the figure legend or in the 'Materials and Methods' section. The editors reserve the right to request original versions of figures and the original images that were used to assemble the figure.

Further information is available in our Guide For Authors:
https://www.embopress.org/page/journal/14602075/authorguide

Please click on the link below to submit the revision. The resubmission deadline is currently set to 12th Apr 2021. Please contact us if you would need an extension.

Link Not Available

Referee #1:

The study by Ortin-Martinez et al. shows that photoreceptors in vivo form microtubule-containing nanotube (NT) protrusions that mediate the transfer of fluorescent protein and mitochondria, from the injected donor photoreceptors to the acceptor photoreceptors in the ONL layer of the retina. In contrast, photoreceptor-derived extracellular vesicles (EVs) has no impact on the transfer of proteins.
One of the weakest points is that the results are organised the way that in vitro and in vivo experiments are mixed within the same section. Due to this, it is not very clear what exactly was shown in vivo. Some of the statements are not supported by in vivo experiments.
For example, the section "Mitochondria transfer in the context of photoreceptor transplantation and co-culture" is concluded by the statement "Thus, using two different mitochondria labelling approaches, we conclude that donor-derived mitochondria can transfer to acceptor photoreceptors in vivo and furthermore, we reveal that this exchange is contact dependent.", which can be

misleading because the necessity of contact for mitochondria transfer was confirmed only in vitro. Likewise, the section "Donor photoreceptor protrusions are the conduits for mitochondria exchange and exhibit structural and morphological characteristics of open-ended nanotubes" is concluded by the statements "Based on these data we suggest that donor photoreceptors extend microtubule+-protrusions to form stable connections with acceptor photoreceptors in vivo. .... Finally, the kinetics of mitochondria transfer along these PhNTs suggests that they are open ended and bridge the cytoplasm between donor and acceptor photoreceptors.", although live experiments on mitochondria transfer were performed only in vitro. Authors do not provide direct evidence on that nanotubes found in vitro and in vivo are the same structures.
Although this is an interesting study that will further solidify the importance of nanotube-like protrusions in direct cell-cell communication and material transfer, the experiments do not always support the conclusion drawn by the authors and many points remain to be addressed.

General comments

1. For all the figures it is difficult to identify the retinal layers in the fluorescence images, even though a white-dashed line is provided to mark the ONL of the recipient retina. Labels should be provided on the side of the fluorescence images to better help the reader discern the relative positioning of the donor cells as compared to the acceptor cells. Throughout all the figures, arrows can be provided that point the reader to representative donor cells and acceptor cells.

2. In all of the figures that show donor and acceptor photoreceptors, the authors should point to donors and acceptors for better distinguishing.

3. In Figure 1, the implanted GFP+ donor cells appear far more proximal to the ONL as compared to B, though in C, very little of the subretinal space is actually shown. In B, the implanted GFP+ donor cells are of the order of 100-150 microns away from the ONL. The presented images in C suggest a far closer contact of the donor cells which could facilitate more GFP transfer in a cell-contact-dependent manner given the far shorter distance to the ONL. Is this distance difference a result of random error during implantation? The authors seem to suggest these grafted donor cells do not migrate (stated on page 5); do they have data to support this in both the WT and Nrl-/- models given this drastic distance difference of the donor cells to the ONL?

4. Figure 1E was not discussed in the result section.

5. On page 7 for the description of Figure 2, the authors immediately start with panel H. The figure should be re-ordered in this case such that the order of the panels in the figure match the discussion of the results in the text.

6. Though the transfer is lower compared to cytosolic GFP, the authors state on page 7 that nuclear-located nls-GFP is also transferred. The authors do not speculate how this transfer is possible for a nuclear resident protein in this case. In this example for nls-GFP I expect the transcript to be transferred to the acceptor and then the acceptor cell starts to express this transcript. The authors' FISH experiments should be done here to confirm this scenario.
7. In regards to Figure 4, the authors fail to address a major discrepancy in their data. Fluorescence images in 4A for Mito-DsRed clearly show little mitochondrial transfer to ONL-resident acceptors cells, while their MitoTracker-based experiments instead suggest a large percentage of transferred mitochondria. It is unclear whether this higher transfer result is actually mediated by transfer of just the MitoTracker dye itself. Cytometry-based measurements must be provided for the Mito-DsRed

case to better quantify mitochondria transfer.
There is a statement: "MitoTracker Red FM, a dye that is trapped in mitochondria at the time of labelling and unincorporated dye cannot be transferred between cells". How can authors support this statement? MTR puncta on the images of panel 4F look evenly distributed, that could happen if the dye had diffused by itself.
8. Figure 4F - do the authors observe red mito puncta also in green acceptor photoreceptors? It is not visible in the images.

9. In Figure 5, the GFP signal is over saturated and it is exceedingly difficult to distinguish between donor cells and the acceptor cells and to discern the morphologies of the connections.

10. My concern is how to properly observe nanotube (NT) structure in cells such as photoreceptors, that are sensory neurons, thus particularly branched (neurites...). For example, in Figure 5A, for structures the authors are pointing at, we cannot be sure these are NTs. Authors should transplant Nrl::GFP labeled with DiI or DiD into the retina and observe a NT that contains a DiI/DiD+ vesicle within it. Otherwise, we cannot be sure from tissue imaging currently available that these structures observed in photoreceptors in the retina are NTs.

11. Figure 5C - could the authors precisely point the protrusions they are referring to? How can the authors claim with certainty that these protrusions at the contact between donor and acceptor photoreceptors are NT-like and not neurite-like?

12. Figure 6 - the authors should reorganize this figure, in particular B part, since it is found over the whole figure and it is not easy to follow what is what in the result section. Additionally, every time the authors refer to a particular NT-like protrusion, as it is not evident from the images, they should point at the protrusion. The authors should clearly address in the result section all the parameters necessary to distinguish between various protrusions found in photoreceptors.

13. A major problem is that the authors cannot conclude with the single-color live imaging data presented in Figure 7 that mitochondria are actually transferred between cells through open-ended connections. In fact, the protrusions the authors present are terminally closed (depicted in EV 7A). This protrusion phenotype is clearly evident in the red channel for Figure 7C where the terminal foot (which is filled with mitochondria) is attached to the bottom right cell. The terminal foot phenotype is further seen in the brightfield image of 7D, where the moving green mitochondria reaches the end of the protrusion and remains exterior to the cell in the bottom left; one does not at all observe the entrance of this mitochondria into the receiving cell. This phenotype is very reminiscent of the inter-pericyte connections observed by Alarcon-Martinez et al. Nature 2020. To properly show cargo transfer, the authors must have cells labelled in different colors to easily distinguish donor cells having labelled cargo from acceptor cell populations. Furthermore, the authors state the "kinetics of mitochondria transfer" suggests the connections are open-ended. This conclusion is not supported by the data. The authors provide no trajectory analysis of organelle movement to definitively show the cargo passing from a donor cell and entering a receiving cell. The data in 7D simply shows the cargo is motile in a connection.

14. Figure 8A and result section - the authors should explain in the result section the rationale behind the usage of ROCK inhibitor. Furthermore, to complete the story of actin cytoskeleton involvement in the formation of open-ended PhNTs, the authors should perform experiments where they assess the role of RhoA and Rac1 on the transfer of e.g. mitochondria.

Additionally, the authors emphasize that NTs they observe are microtubule-based; in contrast, they

only investigate the involvement of actin cytoskeleton. Therefore, the authors should address using at least a drug approach, the involvement of microtubules in the formation of PhNTs and the impact their inhibition has on MT.

15. Figure EV6 is the only one showing the presence of material localised to the connections in vivo. The quality of the image EV6A is not sufficient to provide evidence on that mitochondria are inside of the tube. Moreover, on this image we cannot see the second cell connected by this tube. Finally, there is no data on whether these mitochondria can be transferred to acceptor cell, consequently there is no evidence on that this protrusion is open-ended.

16. Figure EV7A includes thickness measurements of nanotubes in vitro and in vivo. Was the same microscope used for measurements in vivo and in vitro? What was the effective PSF of the microscope, namely did it provide sufficient resolution for such measurements?

17. Movie EV3 shows the presence of "GFP+ transplanted cells" within the "host area". How this corresponds to the fact that transplanted cells do not migrate to the host retina?
-
18. In the coculture experiments there is a control of coculture with dead ROSA mT/mG donor cells. Is the fluorescence of lysed cells preserved at the same level as live cells?

Referee #2:

The manuscript by Ortin-Martinez and colleagues provides a thorough characterization of the nanotubes formed by young photoreceptors, which are either cultured or transplanted subretinally to adult mice. The data are of exceptional quality and conclusions are mostly justified, except from several occasions described below. Overall, this is a very nice study.

Major points:

1. Page 5: "Notably, because these data are normalized to donor cell number, the increase in MT Nrl-/- recipients [in] independent of donor photoreceptor<s> survival." If donor photoreceptor cells die, but the recipients persist, the material transfer efficiency, as calculated here, goes up because this calculation is dependent on survival, as the authors explain in the next sentence: "Taken together, our results show that MT efficiency is, in part, a function of the number of surviving donor photoreceptors..." Thus, in Figures 1B and C, material transfer efficiency is not going up after P21, instead the donor cells are dying. I do not oppose data normalization, but the resulting value should not be called transfer efficiency to avoid any confusion and misunderstanding.
2. The authors insist that the RNA transfer is minimal and even state it in the section title. However, their data show that RNA transfer does take place and the apparent difference from GFP transfer can be easily explained by differences in the sensitivity and linearity of the corresponding measuring techniques. In fact, it is extremely hard to imagine that nanotubes of ~0.7 micron in diameter and allowing the transport of mitochondria can selectively block the transport of RNA but not proteins. Unless the authors provide more compelling evidence that such a mechanism exists, they should refrain from making this conclusion.
3. Page 8: The following argument is not compelling: "The broad apical-basal distribution of transferred mitochondria in acceptor photoreceptors (Fig. 4A) compared with the apically-enriched mitochondria labelling induced by direct lenti-mito-DsRed infection in acceptor cells, argues against

transfer of mito-DsRed protein to acceptor photoreceptors (Fig. EV5A)." Even more importantly, these experiments do not exclude that it was actually mito-DsRed which diffused through the nanotubes before being trapped by the mitochondria in acceptor cells.
4. Figure 4F: "Similarly, we detected MitoTracker-Red+-puncta in acceptor photoreceptors in vivo after donor transplantation (Fig. 4F)." In fact, the red staining pattern does not coincide with mitochondria labeled with mito-DsRed and is not enriched in transferred cells as compare dto surrounding cells. This staining appears random and should not be shown or used as an argument here. I do not think this experiment should be included in this manuscript.

Minor points:
1. The running title has a typo: "trough".
2. Abstract - The term "open-ended" needs to be either defined or not used.
3. Figure 1A cartoons: The nanotubes drawn in these cartoons are connected to outer segments and RPE. However, the actual data (e.g. Fig. 6A and 5C) suggest that they are more likely to be connected to the inner segment/cell body. In fact, it makes more sense that they are connected to the inner segment, as outer segments are highly specialized cilia unlikely to possess machinery required for fusing with these nanotubes. Next, there is no evidence that these nanotubes connect to the RPE, so that small connectors facing the RPE cells may be perceived as misleading. On the other hand, nanotubes between individual transplanted cells are not shown in this cartoon but can be clearly seen in Figure 1F. Finally, the OLM line should be moved down, as OLM is located just above the top nuclei in the photoreceptor layer. The same applies to cartoon used in a supplementary figure.
4. Screen shots in Figure 1E should be cleaned.
5. Page 5 typo: "Notably, MT efficiency in [wiltype] recipients increased from 14 days to reach a maximum..."
6. Page 6: "it could simply be that acceptor photoreceptors have a high propensity to accumulate GFP from any type of cell." I suggest replacing "GFP" with "material" in the context of this sentence.
7. Figure 1F: Why is there no transfer to the photoreceptor layer in this example?
8. Figure 3C: Please show that, after cell lysis, the red fluorescent protein is still fluorescent and therefore could have been detected if transferred into the acceptor cells.
9. Page 8: A word is missing in this sentence: "when green and red MitoTracker-labelled co-cultured were separated by a transwell"
10. Page 9: "In cryosections of Nrl-/- recipient retinas transplanted with Nrl::GFP donor photoreceptors, we observed thin protrusions that appeared to connect GFP+donor and GFP +acceptor photoreceptors (Fig. 5A)..." This observation is not new and needs references to all previous studies.
11. Page 9: "Using a newly established tissue clearing protocol for whole intact eyes (Gurdita et al., in preparation),..." Because this protocol is not even submitted for publication, it cannot be called "established".
12. Page 11: typo: "The [formation membrane] protrusions, including neurites, is regulated..."
13. Page 12: "TNTs that connect immune cells in the cornea (Chinnery, Pearlman et al., 2008) and pericytes in the retinal vasculature that mediate neurovascular coupling (Alarcon-Martinez, Villafranca-Baughman et al., 2020) differ considerably from PhNT since these connect two primary neurons and intracellular cargo can be transferred through them." Please clarify what "these" refers to and explain how they are different.
14. Typo in Figure S6: "localized" is spelled wrong.

Referee #3:

Material transfer (MT) between cells, including neurons by Nanotubes, is established. In this work, the authors probe the mechanism of MT from transplanted photoreceptor progenitors to the recipient cells using multiple approaches. All of them implicate Nanotubes as the likely source of MT, a novel finding in the retina. The physiological significance in the retina, if any, of the open-ended MT, is not clear. The authors claim lineage specificity and the movement of mitochondria bi-directionally. The mechanism by which the mitochondria transfer from the recipient to the transplanted cells is hard to fathom. Yet, the movement of RNA is prohibited. The authors provide no mechanistic insight into the selectivity of the cargo or for the lineage. While the physiological significance of this MT is not known, it may have a therapeutic value in the treatment of retinal disorders. Overall, the manuscript is well-written, with minor typos throughout the document.

Comments:
Fig.1 A shows a scheme where the donor cell transfers materials by NT to the outer segment. Did authors conduct these experiments in prph2 knockout where the OS development is hindered? This will test the requirement for the OS in MT. Based on Fig. 6B, one assumes that OS is not needed, but a quantitative comparison of MT is needed.

Based on the scheme, the assumption then is that the protein transferred by NT to the OS moves to the IS through the CC and other regions of the recipient photoreceptor cell. How do authors envision the transfer of mitochondria in this scenario? Surprisingly, GFP is not observed in the RPE even after the phagocytosis of the GFP+ OS.

The rationale for switching the donor from one experiment to another (C57 to Nrl knockout) without any explanation is unclear. Why is the transfer efficiency lower in 1C vs. 1B at 90 days?

Why is Nrl knockout retina more receptive to MT? What is the proof that donors do not migrate? This is more of an issue when Nrl Knockout is used. I cannot see the donor cells in these experiments.

Based on 1C, one would expect a lot better cre recombination event than what is shown in 1D.

For the control, for example, injection of cortical neurons, why was C57Bl6/J retina used instead of Nrl-/-? Also, the concentration of cell numbers appears to be 4 times less for cortical neurons. Why would there be a need for lineage specificity in transferring materials to the recipient photoreceptor cell?

For cre transduction, it is known that cre recombinase protein can cross the membrane. Alternate mechanisms (other than NT) of cre transfer should be considered.

Fig, 1F, does the transfer require SRS? and if so, why?

The title emphatically states that accumulation is due to protein transfer. While the paragraph describing these results, the conclusions are tempered. "strongly suggests" is more aligned with the experimental results described. Transfer of nls-GFP while lower was still observed. What is the proposed mechanism for the transfer of nls-GFP?

In the in vitro model, why didn't the authors use cortical neurons to show specificity? There is insufficient proof that in vitro system is a surrogate for in vivo transplants. There is no OS in these cultures. There is MT in the in vitro model, but the mechanisms could be different even in the "in vivo" system between Wild-type and Nrl knockout retina.

Fig. 4, Why would Mito-DsRed appear in the nuclear layer? It's a bit confusing when a similar color scheme is used in 4A for nuclei and for ATP5B. In Fig. 4F, since authors know cortical neurons are not conducive for MT, why not use those neurons labeled with MitoTracker Red? This is a comment that can be repeated for most of the "in vitro" experiments conducted to validate the mechanism of MT.

The overexpression of RhoA and RAC1 DN protein in the donor cells (Lenti infected C57bl6/j) needs to be established by ICC or westerns. It appears that the donors with overexpressed tend to aggregate (clump) in the SRS.

Could authors expand on the connectome-associated route of disease-causing protein spread in vivo and provide few examples for the readers?

Minor: Several typos remain. Since the figures were not labeled, it was a difficult manuscript to review.

Point by point response to Reviewers' comments

Referee #1:

The study by Ortin-Martinez et al. shows that photoreceptors in vivo form microtubule-containing nanotube (NT) protrusions that mediate the transfer of fluorescent protein and mitochondria, from the injected donor photoreceptors to the acceptor photoreceptors in the ONL layer of the retina. In contrast, photoreceptor-derived extracellular vesicles (EVs) has no impact on the transfer of proteins. One of the weakest points is that the results are organised the way that in vitro and in vivo experiments are mixed within the same section. Due to this, it is not very clear what exactly was shown in vivo. Some of the statements are not supported by in vivo experiments.

We agree that this is confusing. In response we have revised the manuscript so that the majority of the in vitro and in vivo data are in separate figures:

- *Figures 1, 2, 4 ,5, 6 report in vivo data only*
- *Figures 3 and 7 report in vitro data only.*
- *Figures 8, EV2 and EV4 combine in vivo and in vitro data, which we think is appropriate as they group findings by concept, e.g., the impact of actin remodeling on MT (Fig. 8), role of extracellular vesicles in MT (EV2) and characterization of protrusions (EV4).*

For example, the section "Mitochondria transfer in the context of photoreceptor transplantation and co-culture" is concluded by the statement "Thus, using two different mitochondria labelling approaches, we conclude that donor-derived mitochondria can transfer to acceptor photoreceptors in vivo and furthermore, we reveal that this exchange is contact dependent.", which can be misleading because the necessity of contact for mitochondria transfer was confirmed only in vitro.

We agree with the reviewer and we have revised the results to clearly indicate that the conclusion about cell contact and MT is based on in vitro observations.

Likewise, the section "Donor photoreceptor protrusions are the conduits for mitochondria exchange and exhibit structural and morphological characteristics of open-ended nanotubes" is concluded by the statements "Based on these data we suggest that donor photoreceptors extend microtubule+-protrusions to form stable connections with acceptor photoreceptors in vivo. .... Finally, the kinetics of mitochondria transfer along these PhNTs suggests that they are open ended and bridge the cytoplasm between donor and acceptor photoreceptors.", although live experiments on mitochondria transfer were performed only in vitro. Authors do not provide direct evidence on that nanotubes found in vitro and in vivo are the same structures. Although this is an interesting study that will further solidify the importance of nanotube-like protrusions in direct cell-cell communication and material transfer, the experiments do not always support the conclusion drawn by the authors and many points remain to be addressed.

We agree with the reviewer that we have not directly demonstrated that the connections formed by photoreceptor protrusions are open ended and we have modified our conclusions in the abstract, results and discussion accordingly. In the original submission we provided a comparison of photoreceptor nanotubes in vivo and in vitro (Fig. 6, 7 and Fig. EV4) showing that these structures were similar in length, width, distal morphology and cytoskeletal content. In addition, to address how representative these photoreceptor nanotubes are of the protrusions that can form in vitro, we have also undertaken a comprehensive characterization of in vitro protrusions in live cultures stained with vital actin and tubulin

dyes (see Fig. 7 C, Fig. EV5 and response to comment 13 below). What these analyses show is that the vast majority of protrusions that connect cells in vitro resemble the donor cell protrusions that form in vivo, as they are tubulin+, stable, and end with a distal swelling or spherula-like morphology. After demonstrating the similarity of these protrusions in vivo and in vitro, we used FRAP in vitro to ask if these protrusions mediate transfer. We show that there is GFP recovery in the bleached cell of a pair of protrusion connected photoreceptors in vitro, which suggests that these protrusions can transfer GFP (Fig. 7 D)

General comments

1. For all the figures it is difficult to identify the retinal layers in the fluorescence images, even though a white-dashed line is provided to mark the ONL of the recipient retina. Labels should be provided on the side of the fluorescence images to better help the reader discern the relative positioning of the donor cells as compared to the acceptor cells. Throughout all the figures, arrows can be provided that point the reader to representative donor cells and acceptor cells.

2. In all of the figures that show donor and acceptor photoreceptors, the authors should point to donors and acceptors for better distinguishing.

Response to comments 1 and 2: We have included additional labelling and annotation in the figures and figure legends to indicate the location of donor and acceptor cells and retina layers.

3. In Figure 1, the implanted GFP+ donor cells appear far more proximal to the ONL as compared to B, though in C, very little of the subretinal space is actually shown. In B, the implanted GFP+ donor cells are of the order of 100-150 microns away from the ONL. The presented images in C suggest a far closer contact of the donor cells which could facilitate more GFP transfer in a cell-contact-dependent manner given the far shorter distance to the ONL. Is this distance difference a result of random error during implantation? The authors seem to suggest these grafted donor cells do not migrate (stated on page 5); do they have data to support this in both the WT and Nrl-/- models given this drastic distance difference of the donor cells to the ONL?

We have shown previously that donor cells do not migrate into the ONL in $Nrl^{-/-}$ mice (Ortin-Martinez, Tsai et al., 2017). Moreover, the closer proximity of donor cells to the ONL in $Nrl^{-/-}$ versus wildtype recipients is not a sampling error, as it is observed consistently in the two types of transplant recipients. The objective of this study was to determine whether the correlation between donor and acceptor cell number was consistent across different types of transplant recipients, which we show here (Fig. 1, Fig. EV1). The basis for the enhanced GFP transfer in $Nrl^{-/-}$ recipients is addressed in a separate study (currently under revision) where we show that enhanced GFP transfer in $Nrl^{-/-}$ correlates with closer proximity of donor cells to the host ONL. We speculate that this difference is, in part, because the outer segments of $Nrl^{-/-}$ photoreceptors are shorter than wildtype (Daniele, Lillo et al., 2005), which could permit closer donor-host association after retina reattachment.

4. Figure 1E was not discussed in the result section.

This figure is discussed in the results section as follows: "Notably, MT efficiency in wildtype recipients increased from 14 days to reach a maximum by 21 days post-transplant (Fig. 1 B, C), coinciding with the time when the retinal detachment injury induced by transplant surgery was resolved (Fig. 1 E)."

5. On page 7 for the description of Figure 2, the authors immediately start with panel H. The figure should be re-ordered in this case such that the order of the panels in the figure match the discussion of the results in the text.

The figure is re-organized.

6. Though the transfer is lower compared to cytosolic GFP, the authors state on page 7 that nuclear-located nls-GFP is also transferred. The authors do not speculate how this transfer is possible for a nuclear resident protein in this case. In this example for nls-GFP I expect the transcript to be transferred to the acceptor and then the acceptor cell starts to express this transcript. The authors' FISH experiments should be done here to confirm this scenario.

Using RNAscope we compared GFP transcript distribution in retinas transplanted with donor cells expressing cytosolic and nuclear-localized GFP (Fig. 2 F-K). Transcript levels were similar between both donor cell types; however, we rarely detected any transfer of either type of transcript to acceptor cells. Because of the limitations of the sensitivity of this approach we cannot conclude that transcript transfer never happens, but we can conclude that transfer of nuclear material is not associated with a detectable increase in transcript transfer. We speculate that because nls-GFP is translated in the cytosol, some of it would be available for transfer. However, since nls-GFP would be rapidly imported to the nucleus the relative levels for transfer would be much less compared with cytosolic GFP, which explains why there is less nuclear relative to cytosolic protein transfer.

7. In regards to Figure 4, the authors fail to address a major discrepancy in their data. Fluorescence images in 4A for Mito-DsRed clearly show little mitochondrial transfer to ONL-resident acceptors cells, while their MitoTracker-based experiments instead suggest a large percentage of transferred mitochondria. It is unclear whether this higher transfer result is actually mediated by transfer of just the MitoTracker dye itself. Cytometry-based measurements must be provided for the Mito-DsRed case to better quantify mitochondria transfer. There is a statement: "MitoTracker Red FM, a dye that is trapped in mitochondria at the time of labelling and unincorporated dye cannot be transferred between cells". How can authors support this statement? MTR puncta on the images of panel 4F look evenly distributed, that could happen if the dye had diffused by itself.

8. Figure 4F - do the authors observe red mito puncta also in green acceptor photoreceptors? It is not visible in the images.

Response to points 7 and 8. We thank the reviewer for drawing our attention to the potential confound of MitoTracker dye leakage. While MitoTracker dyes should be retained in healthy mitochondria, in reviewing the literature on this issue, it can indeed be the case that MitoTracker dye can be released from degenerating mitochondria to be taken up by neighbouring cells in culture (Berridge, Herst et al., 2018). To address this issue in our in vivo transplants, we transplanted MitoTracker-labelled WERI tumor cells, a cell type that we have shown does not transfer GFP (Fig. EV1 B). We did observe non-specific MitoTracker

dye labelling in the recipient retina. Thus, it is likely that the MitoTracker transfer from transplanted photoreceptor cells is an artifact of dye transfer. These data are removed from the revised manuscript. To control for non-specific dye transfer between cells in vitro we used low concentrations of dye [as described in (Berridge et al., 2018)] and used a transwell system, which is a standard control to test for non-specific dye uptake from leaking cells (Moschoi, Imbert et al., 2016).

9. In Figure 5, the GFP signal is over saturated and it is exceedingly difficult to distinguish between donor cells and the acceptor cells and to discern the morphologies of the connections.

Donor and acceptor cells are in separate compartments and the border, e.g., the outer limiting membrane (OLM) that separates these compartments is indicated in every image. Additionally, the GFP signal is much stronger in donor cell somas compared to their processes and acceptor cell somas. Thus, visualization of the processes requires oversaturation of the signal in the donor cell soma. The resolution limits of each imaging modality only allow us to discern the morphology of donor-acceptor connections in confocal imaged whole-mount retinas and multi-photon imaged intact cleared eyes.

10. My concern is how to properly observe nanotube (NT) structure in cells such as photoreceptors, that are sensory neurons, thus particularly branched (neurites...). For example, in Figure 5A, for structures the authors are pointing at, we cannot be sure these are NTs. Authors should transplant Nrl::GFP labeled with DiI or DiD into the retina and observe a NT that contains a DiI/DiD+ vesicle within it. Otherwise, we cannot be sure from tissue imaging currently available that these structures observed in photoreceptors in the retina are NTs.

11. Figure 5C - could the authors precisely point the protrusions they are referring to? How can the authors claim with certainty that these protrusions at the contact between donor and acceptor photoreceptors are NT-like and not neurite-like?

Response to points 10 and 11. We have annotated the images in Fig. 5 to point out the donor-derived protrusions. To address the reviewer's request that we localize subcellular vesicles in donor cell protrusions in vivo we assessed co-localization of Mito-DsRed, as this was more feasible than optimizing imaging for DiI/DiO labelling in the timeline for the revisions. While neither approach will distinguish neurites from nanotubes, we reasoned that this data would provide further support that donor-derived protrusions are the conduits for MT. We include improved images in EV5 showing the localization Mito-DsRed+ puncta in a donor cell protrusion that appear to contact the host retina.

We agree with the reviewer that we cannot distinguish whether the donor protrusions are NTs or neurites, and we address this issue in the discussion. The reason we choose to call them ^{Ph}NTs is because of their ability to transfer material, a property that has not been described for "normal" neurites, dendrites or axons. The evidence that ^{Ph}NTs mediate transfer of cytosolic material is the following: acceptor neurons are in contact with donor-derived ^{Ph}NTs, ^{Ph}NTs contain transferrable cargo (e.g. GFP, mitochondria), the presence of similar protrusions connecting photoreceptors and transferring GFP in vitro and, similar to NTs (Abounit, Delage et al., 2015), are suspended above the substrate. Finally, MT correlates with the formation of ^{Ph}NTs, as manipulations that enhance ^{Ph}NT outgrowth promote MT (Fig. 8 A-B) and manipulations that inhibit their outgrowth inhibit MT (Fig. 8 C-D, Appendix Fig. S3).

12. Figure 6 - the authors should reorganize this figure, in particular B part, since it is found over the whole figure and it is not easy to follow what is what in the result section. Additionally, every time the authors refer to a particular NT-like protrusion, as it is not evident from the images, they should point at the protrusion. The authors should clearly address in the result section all the parameters necessary to distinguish between various protrusions found in photoreceptors.

We have reorganized Figure 6 and indicated the protrusions in the panels. Protrusions in vivo were classified on the basis GFP expression and extending from donor cells. Further classification of protrusions in vivo was hampered by donor cell density in the bolus. Because of this limitation we focused our analysis on protrusions that were sufficiently distinct (e.g., extending away from the cell bodies of donor cells) to address cytoskeletal content, apical and basal photoreceptor markers and transferrable cargo.

13. A major problem is that the authors cannot conclude with the single-color live imaging data presented in Figure 7 that mitochondria are actually transferred between cells through open-ended connections. In fact, the protrusions the authors present are terminally closed (depicted in EV5). This protrusion phenotype is clearly evident in the red channel for Figure 7C where the terminal foot (which is filled with mitochondria) is attached to the bottom right cell. The terminal foot phenotype is further seen in the brightfield image of 7D, where the moving green mitochondria reaches the end of the protrusion and remains exterior to the cell in the bottom left; one does not at all observe the entrance of this mitochondria into the receiving cell. This phenotype is very reminiscent of the inter-pericyte connections observed by Alarcon-Martinez et al. Nature 2020. To properly show cargo transfer, the authors must have cells labelled in different colors to easily distinguish donor cells having labelled cargo from acceptor cell populations. Furthermore, the authors state the "kinetics of mitochondria transfer" suggests the connections are open-ended. This conclusion is not supported by the data. The authors provide no trajectory analysis of organelle movement to definitively show the cargo passing from a donor cell and entering a receiving cell. The data in 7D simply shows the cargo is motile in a connection.

We agree with the reviewer that we have overstated the conclusion that the contact sites of protrusions connecting photoreceptors are open-ended and we have removed these statements from the manuscript. The crux of the argument is whether the protrusions that we document in vivo (tubulin+, cargo-containing) allow the passage of cytosolic cargo. One way that we addressed this issue was to characterize protrusions in vitro and to perform FRAP analysis to ask whether connected cells could transfer GFP. We found that the majority of the protrusions that connect photoreceptors are stable, tubulin+, suspended and have terminal swellings where they contact other cells (Fig. 7 C and Fig. EV5). Thus, the majority of the connecting protrusions are as the reviewer points out, terminally closed. Based on FRAP analysis we show that there is approx. 15% recovery of fluorescence in the bleached cell of a pair of connected cells (Fig. 7 D). We do not see this degree of recovery after bleaching isolated cells, suggesting that these closed type protrusions mediate GFP transfer. We acknowledge that we do not know what type of transfer this is and to address this will require more work to determine if there is a degree of cytoplasmic continuity (perhaps by transient membrane fusion), release and uptake of vesicles or even the extension of protrusions at the contact site, for example.

14. Figure 8A and result section - the authors should explain in the result section the rationale behind the usage of ROCK inhibitor. Furthermore, to complete the story of actin cytoskeleton involvement in the formation of open-ended PhNTs, the authors should perform experiments where they assess the role of RhoA and Rac1 on the transfer of e.g., mitochondria.

We thought the reviewer's suggestion to test the effects of RHOA and RAC1 DN on mitochondria transfer was excellent. We have attempted this experiment with two sets of transplantations and unfortunately, we have been beset by technical challenges that preclude having sufficient numbers between groups to draw definitive conclusions, although it does appear RHOA and RAC1 DN reduce Mito-DsRed transfer. We point out that this is a technically challenging experiment that requires double lentivirus infection and a minimum of 5 weeks to carry out. We are disappointed to not provide the in vivo data but to resolve the technical problems and repeat the experiment would result in a further significant delay for resubmission. We hope that the reviewer is understanding in this regard.

We have used an alternative approach to address the question about the role of Rho signalling on mitochondria exchange in vitro. Treatment with ROCK inhibitor has been shown to promote neurite outgrowth in primary neurons (Chen, Hao et al., 2012) and in photoreceptor precursor cells in vitro (Tsai, Ortin-Martinez et al., 2019). Given the resemblance of ^{Ph}NTs to neurites, we reasoned that promoting their outgrowth with ROCK inhibitor could affect material transfer, and we show that this treatment increases GFP transfer in vitro (Fig. 8 A). We explain the rationale for the ROCK inhibitor approach more clearly in the results section. We include new data showing that ROCK inhibitor enhances mitochondria transfer in vitro (Fig. 8 B) further supporting a role for the actin cytoskeleton in the formation of protrusions and mitochondria transfer.

Additionally, the authors emphasize that NTs they observe are microtubule-based; in contrast, they only investigate the involvement of actin cytoskeleton. Therefore, the authors should address using at least a drug approach, the involvement of microtubules in the formation of PhNTs and the impact their inhibition has on MT.

As requested by the reviewer we have investigated the effect of microtubule inhibitors (colchicine, nocodazole) on MT and found that these treatments had no effect on fluorescent protein transfer in vitro (Figure 1 for review, below). There are several explanations for this outcome. Although treatment with these inhibitors did affect the gross morphology of the cultures, including the formation of protrusions connecting cell aggregates, we cannot rule out the possibility that the effectiveness of these drugs wanes over the time frame (3 days) of the assay. Because of toxicity issues we could not re-apply the drugs. It is also possible that there are compensatory mechanisms for MT in the culture system that are microtubule-independent. In future studies it would be important to use genetic approaches to cell-autonomously manipulate microtubule-dependent processes in donor cells, however, these experiments are beyond the scope of the current study.

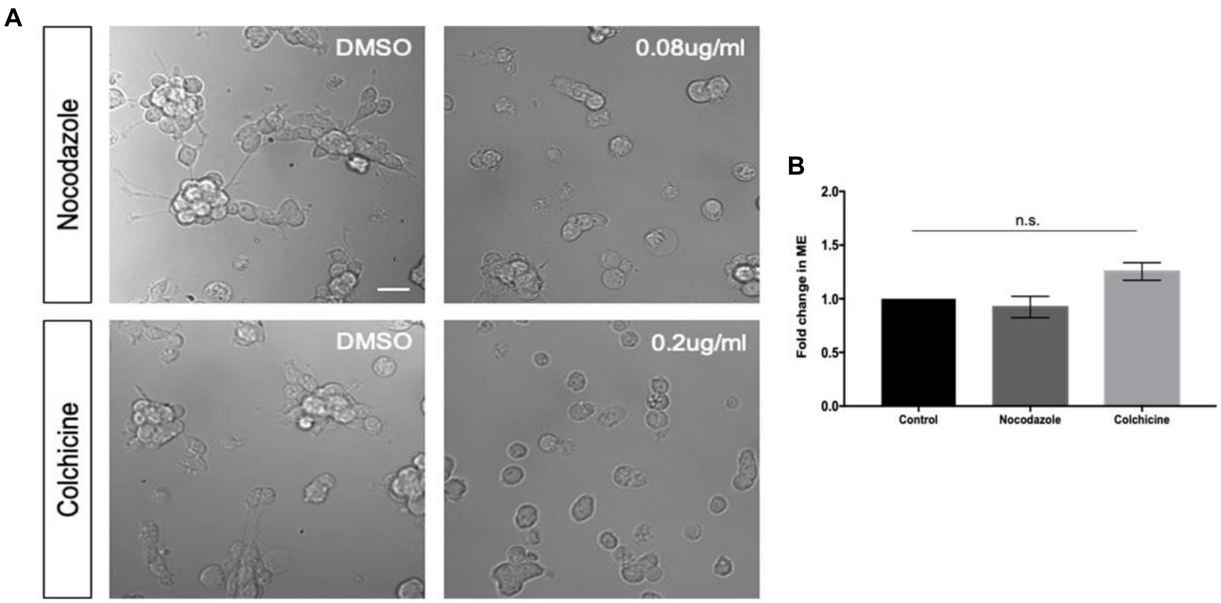

Figure 1. The effect of microtubule disruptors on MT of retinal dissociates in vitro. A. Live confocal images (40x objective) of postnatal day 3-5 C57BL/6J retinal dissociates treated with DMSO, nocodazole, or colchicine, then imaged after 18 hrs treatment. Scale bar: 20μm. **B**. Postnatal day 3-5 Nrl::GFP and ROSAmT/mG retinal co-cultures were treated with 0.08μg/ml nocodazole at the time of plating and 24hr later (n=6) or 0.2μg/ml colchicine at the time of plating (n=3). After three days in vitro, the co-cultures were analyzed by flow cytometry. Quantification of the relative fold change of treated versus DMSO controls showed no differences in protein exchange. All data are presented as mean ± SEM; n.s. not significant, one-way ANOVA followed by Tukey's test multiple comparisons test.

15. Figure EV6 is the only one showing the presence of material localised to the connections in vivo. The quality of the image EV6A is not sufficient to provide evidence on that mitochondria are inside of the tube. Moreover, on this image we cannot see the second cell connected by this tube. Finally, there is no data on whether these mitochondria can be transferred to acceptor cell, consequently there is no evidence on that this protrusion is open-ended.

We provide improved images showing the presence of mitochondria (Mito-DsRed+ puncta) in protrusions that connect donor and acceptor cells in vivo (Fig. EV3 A). We agree with the reviewer that this data does not mean that the connections between donor and acceptor cells are open-ended and we have removed this conclusion from the abstract and results.

16. Figure EV7A includes thickness measurements of nanotubes in vitro and in vivo. Was the same microscope used for measurements in vivo and in vitro? What was the effective PSF of the microscope, namely did it provide sufficient resolution for such measurements?

We appreciate the comment of the reviewer. The PSF for the in vivo measurements in the original submission was sufficient. For the in vitro analyses we repeated the measurements using a PSF of 0.2 and on the same confocal microscope. These data are included in Fig. EV4 C.

17. Movie EV3 shows the presence of "GFP+ transplanted cells" within the "host area". How this corresponds to the fact that transplanted cells do not migrate to the host retina?

Thank you for pointing this out- this was a labelling mistake on our part. Transplanted cells were labelled semi-automatically based on their proximity to the volume reconstruction of the ONL. Cells within the ONL volume were labelled as host PRs while cells outside the ONL volume were labelled as transplanted PRs. The semi-automatically generated ONL volume reconstruction under-represented the true ONL within the image thus mis-labelling some photoreceptors as transplanted photoreceptors. Since the first submission, we have updated our method of generating the ONL volume reconstruction to more accurately resemble the true ONL volume. This was done by manually segmenting the ONL every 100 slices within the image volume before generating the volume reconstruction in Imaris. The 3-D rendering is not corrected and donor and acceptor cells are labelled correctly.

18. In the coculture experiments there is a control of coculture with dead ROSA mT/mG donor cells. Is the fluorescence of lysed cells preserved at the same level as live cells?

We provide images to show the presence of fluorescent debris in co-cultures of live/dead cells (Appendix Fig. S2 D).

Referee #2:

The manuscript by Ortin-Martinez and colleagues provides a thorough characterization of the nanotubes formed by young photoreceptors, which are either cultured or transplanted subretinally to adult mice. The data are of exceptional quality and conclusions are mostly justified, except from several occasions described below. Overall, this is a very nice study.

We thank the reviewer for their positive comments.

Major points:

1. Page 5: "Notably, because these data are normalized to donor cell number, the increase in MT Nrl-/- recipients [in] independent of donor photoreceptor<s> survival." If donor photoreceptor cells die, but the recipients persist, the material transfer efficiency, as calculated here, goes up because this calculation is dependent on survival, as the authors explain in the next sentence: "Taken together, our results show that MT efficiency is, in part, a function of the number of surviving donor photoreceptors..." Thus, in Figures 1B and C, material transfer efficiency is not going up after P21, instead the donor cells are dying. I do not oppose data normalization, but the resulting value should not be called transfer efficiency to avoid any confusion and misunderstanding.

We thank the reviewer for suggesting this interpretation. We agree that the increase in MT in wildtype recipients between P14 and P21 could, in part, reflect persistence of GFP signal in acceptors after donor cell drop out. However, we do not think that this is the explanation for the data in the $Nrl^{-/-}$ recipients because the absolute number of GFP+ acceptor cells is an order of magnitude greater at P21 compared to P14 with a donor cell drop out that is comparable to wildtype recipients. Thus, it is more likely that the increase in MT in $Nrl^{-/-}$ recipients is related to enhanced transfer at the lever of donor and acceptor cells or

enhanced GFP retention in acceptor cells. We agree, however, that we should use a different term and we now call it material transfer index.

2. The authors insist that the RNA transfer is minimal and even state it in the section title. However, their data show that RNA transfer does take place and the apparent difference from GFP transfer can be easily explained by differences in the sensitivity and linearity of the corresponding measuring techniques. In fact, it is extremely hard to imagine that nanotubes of ~0.7 micron in diameter and allowing the transport of mitochondria can selectively block the transport of RNA but not proteins. Unless the authors provide more compelling evidence that such a mechanism exists, they should refrain from making this conclusion.

We agree with the reviewer that we cannot rule out the possibility that transcript transfer contributes to the accumulation of GFP in acceptor cells and we have revised the results section to reflect this point. Our findings indicate that mRNA can transfer between cells but this result does not distinguish whether the accumulation of donor-derived proteins in acceptor cells is due to direct protein transfer or translation from transferred mRNA. In response to Reviewer 1, we asked if differences in transcript transfer could explain the different transfer efficiencies of cytosolic and nuclear-localized GFP (nls-GFP). Transcript levels were comparable between both types of donor cells, while transferred transcripts were nearly undetectable. If transcripts were the primary cargo for transfer, one would expect to see a similar degree of accumulation of cytosolic and nuclear proteins in acceptor cells. Taken together, these observations support a model in which the accumulation of donor-derived material can be a result of mRNA as well as direct protein transfer.

3. Page 8: The following argument is not compelling: "The broad apical-basal distribution of transferred mitochondria in acceptor photoreceptors (Fig. 4A) compared with the apically-enriched mitochondria labelling induced by direct lenti-Mito-DsRed infection in acceptor cells, argues against transfer of mito-DsRed protein to acceptor photoreceptors (Fig. EV5A)." Even more importantly, these experiments do not exclude that it was actually Mito-DsRed which diffused through the nanotubes before being trapped by the mitochondria in acceptor cells.

We agree with the reviewer and we have removed the data on direct lenti-Mito-DsRed infection in adult photoreceptors. Efficient mitochondrial protein import via TOM complex pores requires protein unfolding (Pfanner & Truscott, 2002) and in the case of mito-tagged fluorescent proteins, import is impaired with variants that have rapid folding kinetics (Kashiwagi, Fujioka et al., 2019). Thus, assuming that transferred Mito-DsRed protein would be fully folded, it is unlikely that it would efficiently translocate into the mitochondria of acceptor cells. However, we have modified the interpretation of this experiment to acknowledge the possibility that the Mito-DsRed signal in acceptor cells could be due to transfer of the protein instead of whole mitochondria.

4. Figure 4F: "Similarly, we detected MitoTracker-Red+-puncta in acceptor photoreceptors in vivo after donor transplantation (Fig. 4F)." In fact, the red staining pattern does not coincide with mitochondria labeled with mito-DsRed and is not enriched in transferred cells as compare dto surrounding cells. This staining appears random and should not be shown or used as an argument here. I do not think this experiment should be included in this manuscript.

Please also refer to response to Reviewer 1, Points #7 and #8. We have removed the data on the distribution of MitoTracker signal in transplanted retinas.

Minor points:

1. The running title has a typo: "trough".+

This typo is corrected.

2. Abstract - The term "open-ended" needs to be either defined or not used.

This is statement is removed from the abstract and the results.

3. Figure 1A cartoons: The nanotubes drawn in these cartoons are connected to outer segments and RPE. However, the actual data (e.g. Fig. 6A and 5C) suggest that they are more likely to be connected to the inner segment/cell body. In fact, it makes more sense that they are connected to the inner segment, as outer segments are highly specialized cilia unlikely to possess machinery required for fusing with these nanotubes. Next, there is no evidence that these nanotubes connect to the RPE, so that small connectors facing the RPE cells may be perceived as misleading. On the other hand, nanotubes between individual transplanted cells are not shown in this cartoon but can be clearly seen in Figure 1F. Finally, the OLM line should be moved down, as OLM is located just above the top nuclei in the photoreceptor layer. The same applies to cartoon used in a supplementary figure.

The cartoons are revised to better reflect the experimental setup and observations.

4. Screen shots in Figure 1E should be cleaned.

Unfortunately, these are the best screen shots that could be obtained using the OCT equipment that we had at that time. We have reduced the number of images to 8 from the original 15.

5. Page 5 typo: "Notably, MT efficiency in [wiltype] recipients increased from 14 days to reach a maximum..."

We apologize for the typos-we have gone over the manuscript very carefully to catch and correct them.

6. Page 6: "it could simply be that acceptor photoreceptors have a high propensity to accumulate GFP from any type of cell." I suggest replacing "GFP" with "material" in the context of this sentence. +

We agree and have changed the text.

7. Figure 1F: Why is there no transfer to the photoreceptor layer in this example?

We selected this image because it was a particularly good example of Cre-dependent reporter gene expression in donor cells in the subretinal space. In adjacent sections from the same eye there is transfer of fluorescent protein to the host photoreceptors, as we show here in Figure 2 for review. We should point out that one does not always see transfer in every single section that contains donor cells, which is likely because donor and acceptor cells can be in adjacent sections.

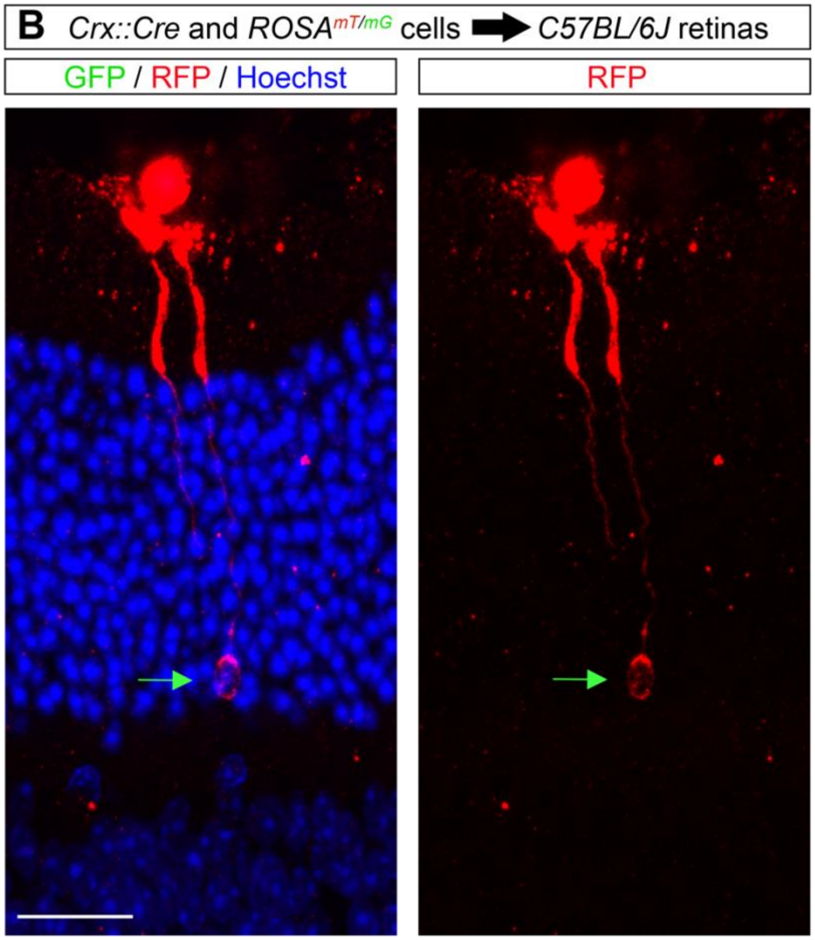

Figure 2. Transfer of TdTomato from transplanted cells to recipient photoreceptor after *Crx::Cre* and *ROSA*$^{mT/mG}$ transplantation in C57BL/6J retinas. Green arrow points the soma of an acceptor photoreceptor. Scale bar: 20 µm

8. Figure 3C: Please show that, after cell lysis, the red fluorescent protein is still fluorescent and therefore could have been detected if transferred into the acceptor cells.

Reviewers 1 and 3 had similar comments. We now include images showing that fluorescent debris is detectable up to 3 days after co-culture (Appendix Fig. S2 D).

9. Page 8: A word is missing in this sentence: "when green and red MitoTracker-labelled co-cultured were separated by a transwell"

This mistake is corrected.

10. Page 9: "In cryosections of Nrl-/- recipient retinas transplanted with Nrl::GFP donor photoreceptors, we observed thin protrusions that appeared to connect GFP+donor and GFP+acceptor photoreceptors (Fig. 5A)..." This observation is not new and needs references to all previous studies.

We thank the reviewer for this suggestion and we reviewed several photoreceptor transplantation publications (including those pre-dating the reports on MT) and we were surprised to only find one paper that shows examples of putative physical contacts between donor and acceptor photoreceptors (Ortin-Martinez et al., 2017) and another describing the extension of protrusions from donor cells (Warre-Cornish, Barber et al., 2014). These papers are now cited appropriately. However, we do want to cite the relevant literature, thus, if the reviewer is could make us aware of other papers with similar observations that we have missed we would be pleased to include them.

11. Page 9: "Using a newly established tissue clearing protocol for whole intact eyes (Gurdita et al., in preparation),..." Because this protocol is not even submitted for publication, it cannot be called "established".

We changed this to "Using a tissue clearing protocol for whole intact eyes (Gurdita et al, iScience, in revision)….

12. Page 11: typo: "The [formation membrane] protrusions, including neurites, is regulated..."

This mistake is corrected.

13. Page 12: "TNTs that connect immune cells in the cornea (Chinnery, Pearlman et al., 2008) and pericytes in the retinal vasculature that mediate neurovascular coupling (Alarcon-Martinez, Villafranca-Baughman et al., 2020) differ considerably from PhNT since these connect two primary neurons and intracellular cargo can be transferred through them." Please clarify what "these" refers to and explain how they are different.

We have explained this better.

14. Typo in Figure S6: "localized" is spelled wrong.

This mistake is corrected.

Referee #3:

Material transfer (MT) between cells, including neurons by Nanotubes, is established. In this work, the authors probe the mechanism of MT from transplanted photoreceptor progenitors to the recipient cells using multiple approaches. All of them implicate Nanotubes as the likely source of MT, a novel finding in

the retina. The physiological significance in the retina, if any, of the open-ended MT, is not clear. The authors claim lineage specificity and the movement of mitochondria bi-directionally. The mechanism by which the mitochondria transfer from the recipient to the transplanted cells is hard to fathom. Yet, the movement of RNA is prohibited. The authors provide no mechanistic insight into the selectivity of the cargo or for the lineage. While the physiological significance of this MT is not known, it may have a therapeutic value in the treatment of retinal disorders. Overall, the manuscript is well-written, with minor typos throughout the document.

We thank the reviewer for their positive comments. We have revised the text to correct the typos.

Comments:
1. Fig.1 A shows a scheme where the donor cell transfers materials by NT to the outer segment. Did authors conduct these experiments in prph2 knockout where the OS development is hindered? This will test the requirement for the OS in MT. Based on Fig. 6B, one assumes that OS is not needed, but a quantitative comparison of MT is needed.

2. Based on the scheme, the assumption then is that the protein transferred by NT to the OS moves to the IS through the CC and other regions of the recipient photoreceptor cell. How do authors envision the transfer of mitochondria in this scenario? Surprisingly, GFP is not observed in the RPE even after the phagocytosis of the GFP+ OS.

Response to comments 1 and 2. The role of OS in MT goes beyond the scope of this study; however, in a separate manuscript currently in revision, we show that MT is increased in Prph2-/- recipients, demonstrating that OS are not required for MT and might possibly impede MT by acting as a physical barrier for donor cell contact with host photoreceptors. In this study, our images suggest that donor cells contact host photoreceptor below the outer segment and we have made this clearer in the schematics in the paper. In the discussion we speculate that transfer could occur via cytoplasmic bridges or endocytic uptake of material released in close proximity to the contact site.

Because the RPE frequently detaches from the retina in tissue sections, we typically crop this layer out in multi-panel images where we want to show donor/host cell interactions. However, we frequently detect GFP in the RPE in the vicinity of transplanted donor cells, but we have not determined whether this GFP uptake is primary (e.g., directly from donor cells) or secondary (e.g., via phagocytosis of host OS), but this is a good question for a future study. Our cell culture studies show that RPE is not required for MT, at least in vitro.

3. The rationale for switching the donor from one experiment to another (C57 to Nrl knockout) without any explanation is unclear. Why is the transfer efficiency lower in 1C vs. 1B at 90 days?

I think that the reviewer is referring to why we use different mouse strains (Wildtype or $Nrl^{-/-}$) as transplant recipients. Previous studies have reported a higher degree of MT in $Nrl^{-/-}$ vs C57BL/6J recipient mice (Decembrini, Martin et al., 2017, Nickerson, Ortin-Martinez et al., 2018, Ortin-Martinez et al., 2017, Waldron, Di Marco et al., 2018). Nonetheless, there are similarities in MT between these recipient strains, including the association between donor and acceptor cell number post-transplant (this study Fig. 1, Fig. EV1) and types of transferred cargo, including GFP and Cre recombinase (Ortin-Martinez et al., 2017, Pearson, Gonzalez-Cordero et al., 2016, Santos-Ferreira, Llonch et al., 2016, Singh, Balmer et al., 2016),

which suggests that at least some features of MT are conserved between both types of recipients. Moreover, GNAT1 transfer, which we evaluate in this study using $Nrl^{-/-}$ recipients (Fig. 2C), has been reported in GNAT1 KO mice (Pearson, Barber et al., 2012, Pearson et al., 2016), providing further support that transferred cargo is to some extent independent of recipient cell genotype. Based on these similarities, we thought it was valid to use $Nrl^{-/-}$ mouse recipients as a sensitized system to evaluate the effects of extracellular vesicle-mediated transfer (Fig. EV2). The other major point that we used $Nrl^{-/-}$ recipients to address is whether the decline in acceptor cells with transferred GFP (Fig. EV1 A) reflects selective degeneration. To address this point in a more sensitive background, we asked if acceptor cells with Cre-induced GFP expression exhibit a similar decline over time (Fig. 1 D).

Regarding differences in transfer efficiency at 90 days is higher in the $Nrl^{-/-}$ (Fig. 1 C) compared with wildtype (Fig. 1 B), which his consistent with the higher level of MT in $Nrl^{-/-}$ recipient.

4. Why is Nrl knockout retina more receptive to MT? What is the proof that donors do not migrate? This is more of an issue when Nrl Knockout is used. I cannot see the donor cells in these experiments.

The mechanism of enhanced MT in $Nrl^{-/-}$ mice is an excellent question, and in a separate manuscript in revision we show that differences in MT correlates with closer proximity of donor cells (observation that reviever#1 made in point#3) to the outer limiting membrane in $Nrl^{-/-}$ recipients compared with wildtype (Gurdita et. al., iScience in revision). We have annotated the images to indicate the position of the donor cells (always apical to the OLM) in $Nrl^{-/-}$ recipients.

5. Based on 1C, one would expect a lot better cre recombination event than what is shown in 1D.

The low level of Cre recombinase transfer (based on reporter gene induction) is consistent with the data in Fig. 2 F-K showing that cytosolic protein transfers more efficiently than nuclear protein.

6. For the control, for example, injection of cortical neurons, why was C57Bl6/J retina used instead of Nrl-/-? Also, the concentration of cell numbers appears to be 4 times less for cortical neurons. Why would there be a need for lineage specificity in transferring materials to the recipient photoreceptor cell?

We used wildtype recipients for this experiment because we wanted to avoid any issues with genetic background affecting the specificity of cell transfer. For example, if we had observed transfer from tumor cells into $Nrl^{-/-}$ recipients we would likely have repeated the experiment in wildtype recipients. We based our cortical neuron transplant procedure on existing transplant protocols for other brain regions. We speculate that the lineage specificity for transfer to host photoreceptors is related to cell adhesion and competence to extend neurites, hence the limited transfer from cortical neurons and no transfer from dividing tumor cells.

7. For cre transduction, it is known that cre recombinase protein can cross the membrane. Alternate mechanisms (other than NT) of cre transfer should be considered.

We agree that it is possible that there could be multiple mechanisms of Cre transfer, which we have not explored here, as it is beyond the scope of this study. Here, we exploited Cre transfer to address different

questions e.g., the persistence of GFP+ acceptor cells (Fig. 1 D) and whether MT occurs between transplanted donor cells (Fig. 1 F).

8. Fig, 1F, does the transfer require SRS? and if so, why?

We have not compared MT between donor cells located in the SRS or in the vitreous (e.g., donor cells that end up in the vitreous in a failed transplant). We speculate that since MT occurs in photoreceptor cultures, the SRS is not required for MT.

9. The title emphatically states that accumulation is due to protein transfer. While the paragraph describing these results, the conclusions are tempered. "strongly suggests" is more aligned with the experimental results described. Transfer of nls-GFP while lower was still observed. What is the proposed mechanism for the transfer of nls-GFP?

We have revised the title to more accurately reflect the data and to leave open the possibility that transcript transfer does occur. Regarding the mechanism of nls-GFP transfer, please refer to our response to Reviewer 1, Point #6. We show that transcript transfer for nuclear and cytosolic GFP is similar (e.g., barely detectable using RNAscope). We speculate that because nuclear proteins are translated in the cytosol there would be some available for transfer but at a lower level of efficiency compared with cytosolic proteins.

10. In the in vitro model, why didn't the authors use cortical neurons to show specificity? There is insufficient proof that in vitro system is a surrogate for in vivo transplants. There is no OS in these cultures. There is MT in the in vitro model, but the mechanisms could be different even in the "in vivo" system between Wild-type and Nrl knockout retina.

We agree with the reviewer that the in vitro assay is not a perfect surrogate for MT in vivo. However, the in vitro assay allows us to test questions that are not feasible in vivo (e.g., transwell assays to test the requirement for cell contact) and to generate hypotheses that can be tested in vivo (e.g., the role of actin cytoskeletal remodelling in MT). Moreover, several lines of evidence indicate that there are many similarities between in vitro and in vivo MT, including the characteristics of the protrusions that connect neurons and the sensitivity of MT to actin remodelling.

In experiments that we did not have the space to include, we did find that there was limited GFP transfer from tumor cells to photoreceptors in vitro, which further validates the in vitro MT assay. If the reviewer feels that it is important to include these data in the manuscript we will do so.

11. Fig. 4, Why would Mito-DsRed appear in the nuclear layer? It's a bit confusing when a similar color scheme is used in 4A for nuclei and for ATP5B. In Fig. 4F, since authors know cortical neurons are not conducive for MT, why not use those neurons labeled with MitoTracker Red? This is a comment that can be repeated for most of the "in vitro" experiments conducted to validate the mechanism of MT.

We have modified Figure 3 (previously Fig. 4). With respect to controls for MTR transfer, this is a clever suggestion and instead of cortical neurons, we transplanted tumor cells labelled with MitoTracker in vivo

and found non-specific MitoTracker labelling in the host retina. This result tells us that we cannot use MitoTracker for this in vivo assay and this experiment has been removed from the manuscript. Regarding the specificity of MitoTracker transfer in vitro please refer to our response to Reviewer 1 Points #7 and #8. Briefly, to control for non-specific dye transfer between cells in vitro we used low concentrations of dye [as described in (Berridge et al., 2018)] and used a transwell system, which is a standard control to test for non-specific dye uptake from leaking cells (Moschoi et al., 2016).

12. The overexpression of RhoA and RAC1 DN protein in the donor cells (Lenti infected C57bl6/j) needs to be established by ICC or westerns. It appears that the donors with overexpressed tend to aggregate (clump) in the SRS.

We have confirmed RHOA and RAC1 DN overexpression in infected donor cells in vivo and these data replace Fig. EV8 B nor in Appendix Fig S3 B . As to whether RHOA or RAC1 DN affect donor cell aggregation, we did not analyse this directly. While it may appear that way in the images shown (which were chosen because they contained similar sized donor cell boluses in the SRS, to more easily compare the impact of these transgenes on MT to host photoreceptors), in the over 30 sections of each retina we analysed for this experiment we did not notice a qualitative difference in donor cell clumping.

13. Could authors expand on the connectome-associated route of disease-causing protein spread in vivo and provide few examples for the readers?

We included additional discussion and references for this topic in the discussion.

14. Minor: Several typos remain. Since the figures were not labeled, it was a difficult manuscript to review.

We have gone over the manuscript very carefully to catch all of the typos.

References

Abounit S, Delage E, Zurzolo C (2015) Identification and Characterization of Tunneling Nanotubes for Intercellular Trafficking. *Curr Protoc Cell Biol* 67: 12 10 1-12 10 21

Berridge MV, Herst PM, Rowe MR, Schneider R, McConnell MJ (2018) Mitochondrial transfer between cells: Methodological constraints in cell culture and animal models. *Anal Biochem* 552: 75-80

Chen J, Hao H, Guo G, Li S, Xiao X (2012) Effect of Rho-kinase pathway on neurite outgrowth of rat hippocampal neurons under atomic force microscopy. *Neural Regen Res* 7: 496-500

Daniele LL, Lillo C, Lyubarsky AL, Nikonov SS, Philp N, Mears AJ, Swaroop A, Williams DS, Pugh EN, Jr. (2005) Cone-like morphological, molecular, and electrophysiological features of the photoreceptors of the Nrl knockout mouse. *Investigative ophthalmology & visual science* 46: 2156-67

Decembrini S, Martin C, Sennlaub F, Chemtob S, Biel M, Samardzija M, Moulin A, Behar-Cohen F, Arsenijevic Y (2017) Cone Genesis Tracing by the Chrnb4-EGFP Mouse Line: Evidences of Cellular Material Fusion after Cone Precursor Transplantation. *Molecular therapy : the journal of the American Society of Gene Therapy* 25: 634-653

Kashiwagi S, Fujioka Y, Satoh AO, Yoshida A, Fujioka M, Nepal P, Tsuzuki A, Aoki O, Paudel S, Sasajima H, Ohba Y (2019) Folding Latency of Fluorescent Proteins Affects the Mitochondrial Localization of Fusion Proteins. *Cell Struct Funct* 44: 183-194

Moschoi R, Imbert V, Nebout M, Chiche J, Mary D, Prebet T, Saland E, Castellano R, Pouyet L, Collette Y, Vey N, Chabannon C, Recher C, Sarry JE, Alcor D, Peyron JF, Griessinger E (2016) Protective mitochondrial transfer from bone marrow stromal cells to acute myeloid leukemic cells during chemotherapy. *Blood* 128: 253-64

Nickerson PEB, Ortin-Martinez A, Wallace VA (2018) Material Exchange in Photoreceptor Transplantation: Updating Our Understanding of Donor/Host Communication and the Future of Cell Engraftment Science. *Frontiers in neural circuits* 12: 17

Ortin-Martinez A, Tsai EL, Nickerson PE, Bergeret M, Lu Y, Smiley S, Comanita L, Wallace VA (2017) A Reinterpretation of Cell Transplantation: GFP Transfer From Donor to Host Photoreceptors. *Stem cells (Dayton, Ohio)* 35: 932-939

Pearson RA, Barber AC, Rizzi M, Hippert C, Xue T, West EL, Duran Y, Smith AJ, Chuang JZ, Azam SA, Luhmann UF, Benucci A, Sung CH, Bainbridge JW, Carandini M, Yau KW, Sowden JC, Ali RR (2012) Restoration of vision after transplantation of photoreceptors. *Nature* 485: 99-103

Pearson RA, Gonzalez-Cordero A, West EL, Ribeiro JR, Aghaizu N, Goh D, Sampson RD, Georgiadis A, Waldron PV, Duran Y, Naeem A, Kloc M, Cristante E, Kruczek K, Warre-Cornish K, Sowden JC, Smith AJ, Ali RR (2016) Donor and host photoreceptors engage in material transfer following transplantation of post-mitotic photoreceptor precursors. *Nature communications* 7: 13029

Pfanner N, Truscott KN (2002) Powering mitochondrial protein import. *Nat Struct Biol* 9: 234-6

Santos-Ferreira T, Llonch S, Borsch O, Postel K, Haas J, Ader M (2016) Retinal transplantation of photoreceptors results in donor-host cytoplasmic exchange. *Nature communications* 7: 13028

Singh MS, Balmer J, Barnard AR, Aslam SA, Moralli D, Green CM, Barnea-Cramer A, Duncan I, MacLaren RE (2016) Transplanted photoreceptor precursors transfer proteins to host photoreceptors by a mechanism of cytoplasmic fusion. *Nature communications* 7: 13537

Tsai ELS, Ortin-Martinez A, Gurdita A, Comanita L, Yan N, Smiley S, Delplace V, Shoichet MS, Nickerson PEB, Wallace VA (2019) Modeling of Photoreceptor Donor-Host Interaction Following Transplantation Reveals a Role for Crx, Muller Glia, and Rho/ROCK Signaling in Neurite Outgrowth. *Stem cells (Dayton, Ohio)* 37: 529-541

Waldron PV, Di Marco F, Kruczek K, Ribeiro J, Graca AB, Hippert C, Aghaizu ND, Kalargyrou AA, Barber AC, Grimaldi G, Duran Y, Blackford SJI, Kloc M, Goh D, Zabala Aldunate E, Sampson RD, Bainbridge JWB, Smith AJ, Gonzalez-Cordero A, Sowden JC et al. (2018) Transplanted Donor- or Stem Cell-Derived Cone Photoreceptors Can Both Integrate and Undergo Material Transfer in an Environment-Dependent Manner. *Stem cell reports* 10: 406-421

Warre-Cornish K, Barber AC, Sowden JC, Ali RR, Pearson RA (2014) Migration, integration and maturation of photoreceptor precursors following transplantation in the mouse retina. *Stem Cells Dev* 23: 941-54

1st Revision - Editorial Decision 9th Jul 2021

Dear Prof. Wallace,

Thank you for submitting a revised version of your manuscript. Your revised study has now been seen by one of the original referees which finds that all the important concerns raised have been addressed. S/he howeverpoints out to a couple of minor modifications that you will need to include in the final text (see below).

In addition, please address the following editorial points:

1. References should be formatted so that a maximum of 10 author names are listed, adding "et al." after the tenth author.
2. EV movie files legends need to be provided together with the movies in a zip file.
3. Appendix should be a single file with a Table of Contents as the first page. Nomenclature should be changed to "Appendix Figure S1", "Appendix Table S1", etc.
4. Source data must be uploaded as a single file per figure.
5. The synopsis figure should be 550 pixels wide and should be in jpeg or png format.

Please let me know if you have any further questions regarding any of these points. Thank you again for giving us the chance to consider your manuscript for The EMBO Journal. I look forward to receiving the final version.

Yours sincerely,

David del Alamo
Editor
The EMBO Journal

Further information is available in our Guide For Authors:
https://www.embopress.org/page/journal/14602075/authorguide

Please click on the link below to submit the revision online:

Link Not Available

Referee #2:

The authors have made an outstanding job in addressing our critical points and suggestions. We only have one remaining request related our original major critique #3 in regard to figure 4. We appreciate the author's response, however because it cannot be excluded that mRNA can be transferred between cells, it has to be stated that mito-DsRed can be transferred by any of three mechanisms: 1) the transfer of whole mitochondria 2) the transfer of mRNA and 3) the transfer of mito-DsRed protein. Otherwise we would like to congratulate the authors on their very impressive study.

The authors performed the requested editorial changes.

2nd Revision - Editorial Decision 19th Jul 2021

Dear Prof. Wallace,

I am pleased to inform you that your manuscript has been accepted for publication in the EMBO Journal.

Congratulations on a very nice study.

Yours sincerely,

David del Alamo
Editor
The EMBO Journal

Please note that it is EMBO Journal policy for the transcript of the editorial process (containing referee reports and your response letter) to be published as an online supplement to each paper. If you do NOT want this, you will need to inform the Editorial Office via email immediately. More information is available here:
https://www.embopress.org/page/journal/14602075/authorguide#transparentprocess

Your manuscript will be processed for publication in the journal by EMBO Press. Manuscripts in the PDF and electronic editions of The EMBO Journal will be copy edited, and you will be provided with page proofs prior to publication. Please note that supplementary information is not included in the proofs.

Please note that you will be contacted by Wiley Author Services to complete licensing and payment information. The 'Page Charges Authorization Form' is available here:
https://www.embopress.org/pb-assets/embo-site/tej_apc.pdf

Should you be planning a Press Release on your article, please get in contact with embojournal@wiley.com as early as possible, in order to coordinate publication and release dates.

EMBO PRESS

YOU MUST COMPLETE ALL CELLS WITH A PINK BACKGROUND ↓

PLEASE NOTE THAT THIS CHECKLIST WILL BE PUBLISHED ALONGSIDE YOUR PAPER

Corresponding Author Name: Valerie A. Wallace

Journal Submitted to: EMBO J

Manuscript Number: EMBOJ-2020-107264

Reporting Checklist For Life Sciences Articles (Rev. June 2017)

This checklist is used to ensure good reporting standards and to improve the reproducibility of published results. These guidelines are consistent with the Principles and Guidelines for Reporting Preclinical Research issued by the NIH in 2014. Please follow the journal's authorship guidelines in preparing your manuscript.

A- Figures

1. Data

The data shown in figures should satisfy the following conditions:

- the data were obtained and processed according to the field's best practice and are presented to reflect the results of the experiments in an accurate and unbiased manner.
- figure panels include only data points, measurements or observations that can be compared to each other in a scientifically meaningful way.
- graphs include clearly labeled error bars for independent experiments and sample sizes. Unless justified, error bars should not be shown for technical replicates.
- if n< 5, the individual data points from each experiment should be plotted and any statistical test employed should be justified
- Source Data should be included to report the data underlying graphs. Please follow the guidelines set out in the author ship guidelines on Data Presentation.

2. Captions

Each figure caption should contain the following information, for each panel where they are relevant:

- a specification of the experimental system investigated (eg cell line, species name).
- the assay(s) and method(s) used to carry out the reported observations and measurements
- an explicit mention of the biological and chemical entity(ies) that are being measured.
- an explicit mention of the biological and chemical entity(ies) that are altered/varied/perturbed in a controlled manner.

- the exact sample size (n) for each experimental group/condition, given as a number, not a range;
- a description of the sample collection allowing the reader to understand whether the samples represent technical or biological replicates (including how many animals, litters, cultures, etc.).
- a statement of how many times the experiment shown was independently replicated in the laboratory.
- definitions of statistical methods and measures:
  - common tests, such as t-test (please specify whether paired vs. unpaired), simple χ2 tests, Wilcoxon and Mann-Whitney tests, can be unambiguously identified by name only, but more complex techniques should be described in the methods section;
  - are tests one-sided or two-sided?
  - are there adjustments for multiple comparisons?
  - exact statistical test results, e.g., P values = x but not P values < x;
  - definition of 'center values' as median or average;
  - definition of error bars as s.d. or s.e.m.

Any descriptions too long for the figure legend should be included in the methods section and/or with the source data.

USEFUL LINKS FOR COMPLETING THIS FORM

http://www.antibodypedia.com	Antibodypedia
http://1degreebio.org	1DegreeBio
http://www.equator-network.org/reporting-guidelines/improving-bioscience-research-repor	ARRIVE Guidelines
http://grants.nih.gov/grants/olaw/olaw.htm	NIH Guidelines in animal use
http://www.mrc.ac.uk/Ourresearch/Ethicsresearchguidance/Useofanimals/index.htm	MRC Guidelines on animal use
http://ClinicalTrials.gov	Clinical Trial registration
http://www.consort-statement.org	CONSORT Flow Diagram
http://www.consort-statement.org/checklists/view/32-consort/66-title	CONSORT Check List
http://www.equator-network.org/reporting-guidelines/reporting-recommendations-for-tumo	REMARK Reporting Guidelines (marker prognostic studies)
http://datadryad.org	Dryad
http://figshare.com	Figshare
http://www.ncbi.nlm.nih.gov/gap	dbGAP
http://www.ebi.ac.uk/ega	EGA
http://biomodels.net/	Biomodels Database
http://biomodels.net/miriam/	MIRIAM Guidelines
http://jjj.biochem.sun.ac.za	JWS Online
http://oba.od.nih.gov/biosecurity/biosecurity_documents.html	Biosecurity Documents from NIH
http://www.selectagents.gov/	List of Select Agents

In the pink boxes below, please ensure that the answers to the following questions are reported in the manuscript itself. Every question should be answered. If the question is not relevant to your research, please write NA (non applicable). We encourage you to include a specific subsection in the methods section for statistics, reagents, animal models and human subjects.

B- Statistics and general methods

Please fill out these boxes ↓ (Do not worry if you cannot see all your text once you press return)

1.a. How was the sample size chosen to ensure adequate power to detect a pre-specified effect size?	Sample size was determined by power calculation to achieve a 80% power and to detect a statistically significant difference at alpha = 0.05. All estimations was done at https://www.sample-size.net/. Ref: Kohn MA, Senyak J. Sample Size Calculators [website]. UCSF CTSI. 19 August 2020. Available at https://www.sample-size.net/
1.b. For animal studies, include a statement about sample size estimate even if no statistical methods were used.	Sample size was determined by power calculation to achieve a 80% power and to detect a statistically significant difference at alpha = 0.05. All estimations was done at https://www.sample-size.net/. Ref: Kohn MA, Senyak J. Sample Size Calculators [website]. UCSF CTSI. 19 August 2020. Available at https://www.sample-size.net/
2. Describe inclusion/exclusion criteria if samples or animals were excluded from the analysis. Were the criteria pre-established?	No inconsistent results were excluded. The exclusion criteria for this study were pre-established. The criteria for exclusion of animals are detailed below in the randomization box.<br>For quantification of protrusions outgrowth, protrusions measuring less than 5 μm in length were excluded from the analysis.
3. Were any steps taken to minimize the effects of subjective bias when allocating animals/samples to treatment (e.g. randomization procedure)? If yes, please describe.	Sample size was determinate by power calculation before experimental procedures. The inclusion and exclusion criteria for this study were pre-established. The authors performed analysis and quantifications in a blind manner. All the statistic tests chosen for comparation between groups were appropriate.
For animal studies, include a statement about randomization even if no randomization was used.	For all transplantation experiments, animals were excluded from the analysis if they showed lack of a cell deposit in the SRS or had evidence of a fulminant immune reaction, because macrophage infiltration is associated with reduced of exchange.<br>In the SD-OCT analysis of the retinal detachment after transplantation, animals were excluded from the study if any complications affecting the opacity of the cornea or lens were observed.
4.a. Were any steps taken to minimize the effects of subjective bias during group allocation or/and when assessing results (e.g. blinding of the investigator)? If yes please describe.	Sample size was determinate by power calculation before experimental procedures. The inclusion and exclusion criteria for this study were pre-established. The authors performed analysis and quantifications in a blind manner. All the statistic tests chosen for comparation between groups were appropriate.
4.b. For animal studies, include a statement about blinding even if no blinding was done	Data analysis prior statistic analysis was carried out with concealment of experimental manipulation or treatment using uniquely coded sample. For statistic analysis data was regrouped in each experimental group for comparisons.
5. For every figure, are statistical tests justified as appropriate?	Yes, they are.
Do the data meet the assumptions of the tests (e.g., normal distribution)? Describe any methods used to assess it.	Experiments in this study were performed independently at least two times (in vivo) and at least three times (in vitro). No inconsistent results were excluded. Flow cytometric analyses of cultured retinal dissociates was performed on at least three independent biological replicates and with at least 2 technical replicates per condition and samples were run on the same cytometers to reduce inter-experimental variability. All the data was collected and organized in Excel's spreadsheet (Office Excel, version 16.35, 2020; Microsoft Corp., Redmond, WA, USA). All the graphical representation and statistical analysis were performed in GraphPad Prism version 7.0a sheets (GraphPad Software Inc. La Jolla, CA, USA). All data in the manuscript is represented as mean ± SEM. Statistical analyses were performed using a one-way ANOVA with Tukey's multiple comparisons test or an unpaired T-test was used were appropriate. The P values are represented as: n.s. no statistically significant, ****p ≤ 0.0001, ***p < 0.001, **p < 0.01, *p < 0.05.
Is there an estimate of variation within each group of data?	All data in the manuscript is represented as mean ± SEM. Statistical analyses were performed using a one-way ANOVA with Tukey's multiple comparisons test or an unpaired T-test was used were appropriate. The P values are represented as: n.s. no statistically significant, ****p ≤ 0.0001, ***p < 0.001, **p < 0.01, *p < 0.05.
Is the variance similar between the groups that are being statistically compared?	For every ANOVA test analysis we additionally performed a Brown-Forstythe test to iverify the equialty of group variances.

C- Reagents

6. To show that antibodies were profiled for use in the system under study (assay and species), provide a citation, catalog number and/or clone number, supplementary information or reference to an antibody validation profile. e.g., Antibodypedia (see link list at top right), 1DegreeBio (see link list at top right).	Primary and secondary antibodies used for immunohistochemistry<br>Antibody / Species / Dilution / Cat #, Supplier<br>Primary Antibodies<br>Anti-EGFP / Goat / 1:500 / 600-101-215, Rockland Immunochemicals, Inc. Pottstown, PA, USA.<br>Anti-RFP / Rabbit / 1:500 / 600-401-379, Rockland Immunochemicals, Inc. Pottstown, PA, USA.<br>Anti-GNAT1 / Rabbit / 1:200 / Sc-389, Santa Cruz Biotechnology, Inc. Mississauga, ON, Canada.<br>Anti-ATP5B / Mouse / 1:500 / Ab5432, Abcam plc. Cambridge, UK.<br>Anti-Prphr2 / Mouse / 1:10 (supernatant) / Gif from Dr. Robert S Molday ref: Hum Mol Genet. 27:295-306 (2018).<br>Anti-αTubulin / Mouse / 1:200 / Sc-53646, Santa Cruz Biotechnology, Inc. Mississauga, ON, Canada.<br>Anti-βTubulin / Mouse / 1:100 / MA5-11732, Thermo Fisher Scientific, Mississauga, ON, Canada.<br>Anti-SV2A / Mouse / 1:100 / SV2, Developmental Studies Hybridoma Bank, University of Iowa, Iowa City, IA, USA.<br>Anti-Bassoon / Mouse / 1:5000 / ADI-VAM-PS003-D, Enzo Life Sciences, Inc., Farmingdale, NY, USA<br>Anti-α βTubulin / Rabbit / 1:100 / 2148, Cell Signaling Technologies, Danvers, MA, USA<br>Secondary Antibodies<br>Anti-goat 488 / Donkey / 1:500 / A-11055, Thermo Fisher Scientific, Mississauga, ON, Canada.

	Anti-mouse 555 / Donkey / 1:500 / A-31570, Thermo Fisher Scientific, Mississauga, ON, Canada.<br>Anti-mouse 647 / Donkey / 1:500 / A-31571, Thermo Fisher Scientific, Mississauga, ON, Canada.<br>Anti-rabbit 555 / Donkey / 1:500 / A-31572, Thermo Fisher Scientific, Mississauga, ON, Canada.<br>Anti-rabbit 647 / Donkey / 1:500 / A-31573, Thermo Fisher Scientific, Mississauga, ON, Canada.<br><br>Antibodies used for western blotting<br>Antibody / Species / Dilution / Cat #, Supplier<br>Primary Antibodies<br>Anti-βTubulin / Rabbit / 1:1000 / Ab6046, Abcam plc. Cambridge, UK.<br>Anti-EGFP / Goat / 1:500 / 1:1000 600-101-215, Rockland Immunochemicals, Inc. Pottstown, PA, USA.<br>Anti-Flotilin1 / Mouse / 1:800 / 610821, BD Transduction LaboratoriesTM, San Jose, CA, USA.<br>Anti-GAPDH / Mouse / 1:10000 / CB1001, Calbiochem, Merck Millipore Sigma Aldrich, Oakville, ON, Canada<br>Anti-RhoA / Rabbit / 1:1000 / 2117, Cell Signaling Technologies, Danvers, MA, USA.<br>Anti-Rac1 / Mouse / 1:1000 / 05-389, Merck Millipore Sigma Aldrich, Oakville, ON, Canada.<br>Secondary Antibodies<br>Anti-goat IRDye 800CW / Donkey / 1:10000 / 926-32214, LI-COR Biosciences, Lincoln, NE, USA.<br>Anti-goat IRDye 680RD / Donkey / 1:10000 / 926-68074, LI-COR Biosciences, Lincoln, NE, USA.<br>Anti-mouse IRDye 800CW / Donkey / 1:10000 / 926-32212, LI-COR Biosciences, Lincoln, NE, USA.<br>Anti-mouse IRDye 680RD / Donkey / 1:10000 / 926-68072, LI-COR Biosciences, Lincoln, NE, USA.<br>Anti-rabbit IRDye 800CW / Donkey / 1:10000 / 926-32213, LI-COR Biosciences, Lincoln, NE, USA.<br>Anti-rabbit IRDye 680RD / Donkey / 1:10000 / 926-68073, LI-COR Biosciences, Lincoln, NE, USA.<br><br>Antibodies used for flow cytometry<br>Antibody / Species / Dilution / Cat #, Supplier<br>Anti-mouse CD73-APC / Rat / 0.05ug/ul / 127210, BioLegend, San Diego, CA, USA<br>Anti-mouse CD73-PE / Rat / 0.02ug/ul / 127206, BioLegend, San Diego, CA, USA<br><br>The following antibodies used in this study have been extensively validate by us and others as per the publications:<br>Anti-EGFP:<br>Ortin-Martinez A, Tsai EL, Nickerson PE, Bergeret M, Lu Y, Smiley S, Comanita L, Wallace VA. A Reinterpretation of Cell Transplantation: GFP Transfer From Donor to Host Photoreceptors. Stem Cells. 2017 Apr;35(4):932-939. doi: 10.1002/stem.2552. Epub 2017 Jan 19. PMID: 27977075.<br>Anti-RFP:<br>Ortin-Martinez A, Tsai EL, Nickerson PE, Bergeret M, Lu Y, Smiley S, Comanita L, Wallace VA. A
7. Identify the source of cell lines and report if they were recently authenticated (e.g., by STR profiling) and tested for mycoplasma contamination.	WERI-Rb-1 (ATCC® HTB-169™) were obtained from Dr. Bremner Lab; STR profiling were performed (Oncogene. 2020; 39(31): 5338–5357; PMID: 32572160).<br>BT88 (ATCC® CRL-3417™) The BT088 cell line was authenticated using STR profiling using a 16 panel Identifiler panel in Dr.Carol Schuurmans Lab.<br>WERI-Rb-1 were routinely screened for mycoplasma by PCR and it was negative.<br>BT088 cell line was not tested for mycoplasma contamination.

* for all hyperlinks, please see the table at the top right of the document

D- Animal Models

8. Report species, strain, gender, age of animals and genetic modification status where applicable. Please detail housing and husbandry conditions and the source of animals.	Mice were housed at the ARC facilities in the Krembil Research Institute in temperature- and light-controlled rooms with a 12 h:12 h light-dark cycle and had food and water ad libitum.<br>Strain // Background // Reference<br>C57BL/6J // N/A // The Jackson Laboratory Stock #000664<br>Nrl::GFP // C57BL/6J // Akimoto, M. et al. Targeting of GFP to newborn rods by Nrl promoter and temporal expression profiling of flow-sorted photoreceptors. Proceedings of the National Academy of Sciences of the United States of America 103, 3890-3895 (2006).<br>Nrl-/- // C57BL/6J // Mears, A.J. et al. Nrl is required for rod photoreceptor development. Nat Genet 29, 447-452 (2001).<br>ROSAmT/mG // Mixed // Gt(ROSA)26Sortm4(ACTB-tdTomato,-EGFP)Luo/J (Jackson Labs Stock #007576)<br>Nrl-/-; ROSAmT/mG // Mixed // Generated by crossing Nrl-/-and ROSAmT/mG (above)<br>Crx::Cre // C57BL/6J // Prasov, L. & Glaser, T. Pushing the envelope of retinal ganglion cell genesis: context dependent function of Math5 (Atoh7). Developmental biology 368, 214-230 (2012).<br>Nrl::GFP; Prphr2-/- // C57BL/6J // Generated by crossing Nrl::GFP and C3A.Cg-Pde6b+ Prph2Rd2/J (Jackson Labs Stock #001979).
9. For experiments involving live vertebrates, include a statement of compliance with ethical regulations and identify the committee(s) approving the experiments.	Animal husbandry was in accordance with the Association for Research in Vision and Ophthalmology (ARVO) Statement for the Use of Animals in Ophthalmic and Vision Research. Animals were maintained under standard laboratory conditions and all procedures were performed in conformity with the University Health Network Animal Care Committee (protocol 3499.17) and adhered to the guidelines of the Canadian Council on Animal Care.
10. We recommend consulting the ARRIVE guidelines (see link list at top right) (PLoS Biol. 8(6), e1000412, 2010) to ensure that other relevant aspects of animal studies are adequately reported. See author guidelines, under 'Reporting Guidelines'. See also: NIH (see link list at top right) and MRC (see link list at top right) recommendations. Please confirm compliance.	Compliance comfirmed

E- Human Subjects

11. Identify the committee(s) approving the study protocol.	N/A
12. Include a statement confirming that informed consent was obtained from all subjects and that the experiments conformed to the principles set out in the WMA Declaration of Helsinki and the Department of Health and Human Services Belmont Report.	N/A
13. For publication of patient photos, include a statement confirming that consent to publish was obtained.	N/A
14. Report any restrictions on the availability (and/or on the use) of human data or samples.	N/A
15. Report the clinical trial registration number (at ClinicalTrials.gov or equivalent), where applicable.	N/A
16. For phase II and III randomized controlled trials, please refer to the CONSORT flow diagram (see link list at top right) and submit the CONSORT checklist (see link list at top right) with your submission. See author guidelines, under 'Reporting Guidelines'. Please confirm you have submitted this list.	N/A
17. For tumor marker prognostic studies, we recommend that you follow the REMARK reporting guidelines (see link list at top right). See author guidelines, under 'Reporting Guidelines'. Please confirm you have followed these guidelines.	N/A

F- Data Accessibility

18: Provide a "Data Availability" section at the end of the Materials & Methods, listing the accession codes for data generated in this study and deposited in a public database (e.g. RNA-Seq data: Gene Expression Omnibus GSE39462, Proteomics data: PRIDE PXD000208 etc.) Please refer to our author guidelines for 'Data Deposition'.<br><br>Data deposition in a public repository is mandatory for:<br>a. Protein, DNA and RNA sequences<br>b. Macromolecular structures<br>c. Crystallographic data for small molecules<br>d. Functional genomics data<br>e. Proteomics and molecular interactions	All data analyzed in this study are provided in this manuscript and supplementary information files. There are no restrictions from the authors on data availability to the publisher and referees, including all raw data.
19. Deposition is strongly recommended for any datasets that are central and integral to the study; please consider the journal's data policy. If no structured public repository exists for a given data type, we encourage the provision of datasets in the manuscript as a Supplementary Document (see author guidelines under 'Expanded View' or in unstructured repositories such as Dryad (see link list at top right) or Figshare (see link list at top right).	This study includes no data deposited in external repositories
20. Access to human clinical and genomic datasets should be provided with as few restrictions as possible while respecting ethical obligations to the patients and relevant medical and legal issues. If practically possible and compatible with the individual consent agreement used in the study, such data should be deposited in one of the major public access-controlled repositories such as dbGAP (see link list at top right) or EGA (see link list at top right).	N/A
21. Computational models that are central and integral to a study should be shared without restrictions and provided in a machine-readable form. The relevant accession numbers or links should be provided. When possible, standardized format (SBML, CellML) should be used instead of scripts (e.g. MATLAB). Authors are strongly encouraged to follow the MIRIAM guidelines (see link list at top right) and deposit their model in a public database such as Biomodels (see link list at top right) or JWS Online (see link list at top right). If computer source code is provided with the paper, it should be deposited in a public repository or included in supplementary information.	N/A

G- Dual use research of concern

22. Could your study fall under dual use research restrictions? Please check biosecurity documents (see link list at top right) and list of select agents and toxins (APHIS/CDC) (see link list at top right). According to our biosecurity guidelines, provide a statement only if it could.	N/A
